# Wiskott-Aldrich syndrome protein forms nuclear condensates and regulates alternative splicing

**DOI:** 10.1038/s41467-022-31220-8

**Published:** 2022-06-25

**Authors:** Baolei Yuan, Xuan Zhou, Keiichiro Suzuki, Gerardo Ramos-Mandujano, Mengge Wang, Muhammad Tehseen, Lorena V. Cortés-Medina, James J. Moresco, Sarah Dunn, Reyna Hernandez-Benitez, Tomoaki Hishida, Na Young Kim, Manal M. Andijani, Chongwei Bi, Manching Ku, Yuta Takahashi, Jinna Xu, Jinsong Qiu, Ling Huang, Christopher Benner, Emi Aizawa, Jing Qu, Guang-Hui Liu, Zhongwei Li, Fei Yi, Yanal Ghosheh, Changwei Shao, Maxim Shokhirev, Patrizia Comoli, Francesco Frassoni, John R. Yates, Xiang-Dong Fu, Concepcion Rodriguez Esteban, Samir Hamdan, Juan Carlos Izpisua Belmonte, Mo Li

**Affiliations:** 1 Bioscience Program, Biological and Environmental Science and Engineering Division, King Abdullah University of Science and Technology (KAUST), Thuwal, 23955-6900 Kingdom of Saudi Arabia; 2 Gene Expression Laboratory, Salk Institute for Biological Studies, 10010 North Torrey Pines Road, La Jolla, CA 92037 USA; 3 Institute for Advanced Co-Creation Studies, Graduate School of Engineering Science, Osaka University, Osaka, Japan; 4 Department of Cell Biology, Scripps Research Institute, La Jolla, CA 92037 USA; 5 The Waitt Advanced Biophotonics Core Facility, Salk Institute for Biological Studies, 10010 North Torrey Pines Road, La Jolla, CA 92037 USA; 6 Altos Labs, Inc. 5510 Morehouse Drive, Suite 300, San Diego, CA 92121 USA; 7 Laboratory of Biological Chemistry, School of Pharmaceutical Sciences, Wakayama Medical University, 25-1 Shitibancho, Wakayama, Wakayama 640-8156 Japan; 8 Next-generation sequencing core, Salk Institute for Biological Studies, 10010 North Torrey Pines Road, La Jolla, CA 92037 USA; 9 Life Science Center, Tsukuba Advanced Research Alliance, University of Tsukuba, 1-1-1 Tennoudai, Tsukuba, Ibaraki 305-8577 Japan; 10 Department of Cellular & Molecular Medicine, University of California at San Diego, La Jolla, CA 92093 USA; 11 Integrative Genomics and Bioinformatics Core, Salk Institute for Biological Studies, 10010 North Torrey Pines Road, La Jolla, CA 92037 USA; 12 State Key Laboratory of Membrane Biology, Institute of Zoology, Chinese Academy of Sciences, Beijing, 100101 China; 13 University of Southern California, 1333 San Pablo Street, MMR 618, Los Angeles, CA 90033 USA; 14 Ambys Medicines, 131 Oyster Point Blvd. Suite 200, South San Francisco, CA 94080 USA; 15 Pediatric Hematology/Oncology and Cell Factory, Fondazione IRCCS Policlinico San Matteo, Pavia, Italy; 16 Department of Research Laboratories and Director of Center for Stem Cell and Cell Therapy, Instituto G. Gaslini Children Hospital Scientific Institute, 16147 Genova, Italy

**Keywords:** Biological techniques, Stem cells

## Abstract

The diverse functions of WASP, the deficiency of which causes Wiskott-Aldrich syndrome (WAS), remain poorly defined. We generated three isogenic WAS models using patient induced pluripotent stem cells and genome editing. These models recapitulated WAS phenotypes and revealed that WASP deficiency causes an upregulation of numerous RNA splicing factors and widespread altered splicing. Loss of WASP binding to splicing factor gene promoters frequently leads to aberrant epigenetic activation. WASP interacts with dozens of nuclear speckle constituents and constrains SRSF2 mobility. Using an optogenetic system, we showed that WASP forms phase-separated condensates that encompasses SRSF2, nascent RNA and active Pol II. The role of WASP in gene body condensates is corroborated by ChIPseq and RIPseq. Together our data reveal that WASP is a nexus regulator of RNA splicing that controls the transcription of splicing factors epigenetically and the dynamics of the splicing machinery through liquid-liquid phase separation.

## Abstract (web summary)

Wiskott-Aldrich syndrome is caused by mutations in WASP, but the underlying mechanisms remain to be explored. Here the authors reveal that WASP deficiency results in aberrant RNA splicing, and that WASP regulates the transcription of splicing factor genes and co-transcriptional RNA splicing via a phase-separation process that involves splicing factors and nascent RNA.

## Introduction

Wiskott–Aldrich syndrome protein (WASP) is the founding member of a family of actin nucleation factors that also include N-WASP (neural WASP), SCAR/WAVE, WHAMM, and WASH^1,2^. WASP family proteins respond to various cellular signals to promote actin polymerization via the Arp2/3 complex^2^. Mutations in WASP cause Wiskott–Aldrich syndrome (WAS), an X-linked primary immunodeficiency that manifests in microthrombocytopenia, eczema, recurrent infections, autoimmunity and predisposition to malignancy^3^. Some but not all disease phenotypes can be linked to the actin nucleation function of WASP^1,4^. It has been revealed recently that WASP family proteins function in the nucleus. For example, *Drosophila* WASH regulates nuclear organization, especially in the heterochromatin compartment^5^. WASP also functions in transcriptional regulation during differentiation of T helper 1 (TH1) cells^4,6,7^ and homology-directed repair^8^. Despite these advances, the multifaceted functions of WASP remain poorly understood.

Precursor mRNA splicing, a process that removes introns from the primary transcript to generate the mature mRNA, contributes to the expression of above 95% of human genes^9^. It is also a complex and fine-tuned process that involves a large number of splicing factors (SFs). Mutations or dysregulated expression of RNA SFs could result in altered RNA splicing that is associated with cancer^10^. Previous research found increased intron retention and decreased productive splicing of mRNA of IFNG and TBX21 in WASP-deficient T_H_1 cells, which correlated with R-loop accumulation at these loci^11^. However, it remains unclear if WASP directly participates in RNA splicing.

Cells perform many biochemical processes including transcription, chromatin organization, RNA splicing, and receptor activation and signaling in diverse membraneless cellular compartments, referred to as condensates, which assemble through liquid–liquid phase separation (LLPS)^12^. N-WASP (encoded by the *WASL* gene)—a ubiquitously expressed WASP family protein–forms two-dimensional micron-scale phase-separated signaling clusters on the plasma membrane through multivalent interactions with the adhesion receptor Nephrin and its cytoplasmic adaptor Nck^13^. The N-WASP-Nephrin-Nck condensates control actin assembly by the Arp2/3 complex^14^. Using an optogenetic tool^15^, a recent study showed that N-WASP can form phase-separated liquid droplets with RNA Pol II and nuclear actin^16^. WASP shares 50% homology with N-WASP. It remains unknown if WASP participates in LLPS and exerts its function in condensates, and, more importantly, what (if any) mechanistic links there are between condensates and the pathogenesis of WAS. In addition, as LLPS is the organizing principle of many nuclear compartments^17^, it could provide a framework to study the nuclear functions of WASP, which remain underexplored.

Research in WAS faces several challenges. Murine models of WAS recapitulate some aspects of WAS, but they lack the presentation of microthrombocytopenia, eczema, and hematological malignancies, yet show consistent development of chronic colitis that is rarely seen in patients^18^. Primary patient cells for research are scarce, owing to the rarity of WAS. Cell lines can be used to study WAS pathogenesis, but the findings in transformed cell lines must be verified in a normal cellular context. Mutations in the *WAS* gene affect all hematopoietic lineages except for erythrocytes^19^. Patient-derived induced pluripotent stem cells (iPSCs) can differentiate into any cell type, therefore constituting an attractive way to model diseases^20–22^. Two WAS models based on patient-specific iPSCs have been reported^23,24^. However, these models lacked an isogenic wild-type control, nor did they provide new mechanistic insights into the disease. Similarly, isogenic pluripotent stem cell (PSC) models of inborn blood disorders can be generated de novo from wild-type cells using genome editing technologies^25^, which afford greater flexibility in modeling a wide range of mutations and avoid confounding factors associated with patient cells (e.g., mutational burden in Fanconi anemia patient cells^25^).

**Fig. 1:**
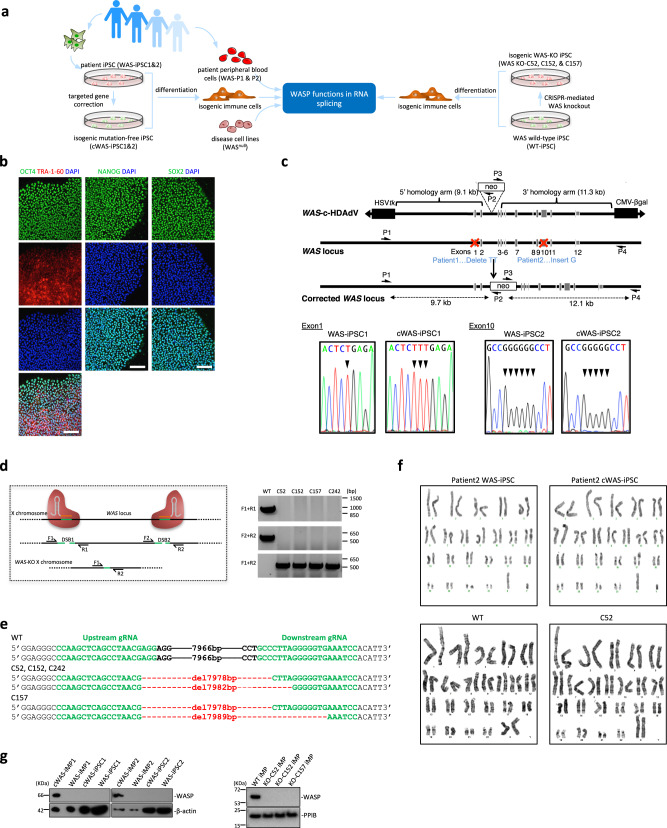
Generation of isogenic iPSC models of WAS. **a** Schematic diagram of the overall study design (created with smart.servier.com). **b** Representative immunofluorescence images of pluripotency markers OCT4, Tra-1-60, NANOG, and SOX2 in WAS-iPSC. DNA was stained with DAPI. Bar, 100 mm. The experiment was repeated 4 times independently. **c** Schematic molecular representation of *WAS* gene correction with gene-correction vector (*WAS*-c-HDAdV). The primers for PCR verification of gene targeting are shown as arrows (P1, P2, P3, and P4). HSV*tk* stands for herpes simplex virus thymidine kinase gene cassette used for negative selection; neo stands for neomycin-resistant gene cassette used for positive selection; CMV-βgal: β-gal expression cassette for determination of HDAdV titer; Red crosses mark the mutations c.107_108del in exon 1 and c.1271dupG in exon 10 present in Patient 1 and Patient 2, respectively. **d** The schematic of the CRISPR design, and representative PCR genotyping results of four iPSC clones with the entire *WAS* gene deleted. DBS: double-strand break, the experiment was repeated three times independently. bp base pair. **e** Sanger sequencing of the PCR product of F1 + R2 shows the CRISPR-induced deletion breakpoints in four KO iPSC clones. **f** Representative karyotyping analysis revealed normal karyotypes in randomly selected clones of WAS-iPSC lines, either before or after gene editing. **g** Western blot analysis of WASP protein in iMPs and iPSCs. Beta-actin or PPIB was used as a loading control. kDa: kilodalton (applicable to all western blot images).

Here, we generate two isogenic models of WAS based on either patient-specific iPSCs followed by precise gene correction of disease-causing mutations or on *WAS* knockout iPSCs generated de novo from a wild-type iPSC line by CRISPR-mediated gene deletion (Fig. 1a). Combined with efficient hematopoietic differentiation these models recapitulate WAS phenotypes and revealed roles of WASP in alternative splicing. Pathogenic mutations or deletion of the *WAS* gene disrupt this process. Briefly, we find that RNA SFs such as SRSF2 were overexpressed in WASP-deficient cells. Knocking down SRSF2 expression in WASP-deficient cells partially rescues altered RNA splicing. Mechanistically, genome-wide WASP binding is enriched at the promoter of RNA SFs. These SFs show a gain of the H3K27ac modification in WAS mutants, suggesting a mechanism of transcriptional regulation. We prove that WASP can form LLPS condensates in cells using the Cry2-based optoDroplet system. A subset of WASP condensates cluster with actively transcribing Pol II, SRSF2, and nascent RNA, suggesting a direct involvement of WASP in RNA splicing. Imaging and biochemical evidence show that WASP physically interacts with SFs and RNA. Fluorescence recovery after photobleaching (FRAP) experiments show that WASP normally acts to constrain the mobility of SRSF2. Taken together, our findings provide mechanistic insights into the role of WASP in regulating RNA splicing.

## Results

### Generation of isogenic WAS-iPSC models

We derived integration-free iPSCs from fibroblasts of two patients with WAS as previously described (Supplementary Fig. S1a–c)^26,27^. Patient 1 had a clinical score 4 on the Ochs scale^28,29^, and a nonsense mutation (c.107_108del, p.F36*). Patient 2 (clinical score unknown) carried a previously unreported mutation (c.1271dupG, p.L425fs). The patient cells showed an expected reprogramming efficiency (Patient 1, 0.097%, Patient 2, 0.08%, and normal range, 0.023–0.2%^25,26^). The established WAS-iPSC lines expressed markers of pluripotency, differentiated into all three germ layers (Fig. 1b and Supplementary Fig. S1d), retained the original *WAS* mutation (Supplementary Fig. S1e), and demonstrated a normal karyotype (Fig. 1f and Supplementary Fig. S1g).

To generate an isogenic wild-type control of WAS-iPSC, we performed targeted gene correction by homologous recombination using a helper-dependent adenoviral vector (HDAdV)–*WAS-*c*-*HDAdV^30–33^ (Fig. 1c). The efficiency of gene correction was 5–50% (Supplementary Fig. S1f). The corrected WAS-iPSCs (named cWAS-iPSCs) had a normal karyotype, retained pluripotency, and were free of random vector integration (Fig. 1f and Supplementary Fig. S1g, h).

The genotype–phenotype relationship in WAS is complex. There are over 200 mutations that lead to hypomorphic levels or complete loss of WASP, and it is impossible to predict clinical severity from the genotype^6,34^. To avoid phenotype ambiguity due to mutational background and ascertain the true loss-of-function phenotype, we deleted the entire *WAS* gene in a well-characterized wild-type iPSC line^26,33^ using the CRISPR-CAS9 system (Fig. 1d, e). WAS knockout had no appreciable effect on pluripotency and karyotype (Fig. 1f and Supplementary Fig. S1g, i). These WAS wild-type iPSCs (WT-iPSCs) and isogenic knockout iPSCs (KO-iPSCs, three independent clones with two different breakpoints, see Fig. 1e) constitute an independent bona fide null WAS-iPSC model that complements the patient iPSC models.

### Isogenic WAS-iPSC models recapitulate disease phenotypes

**Fig. 2:**
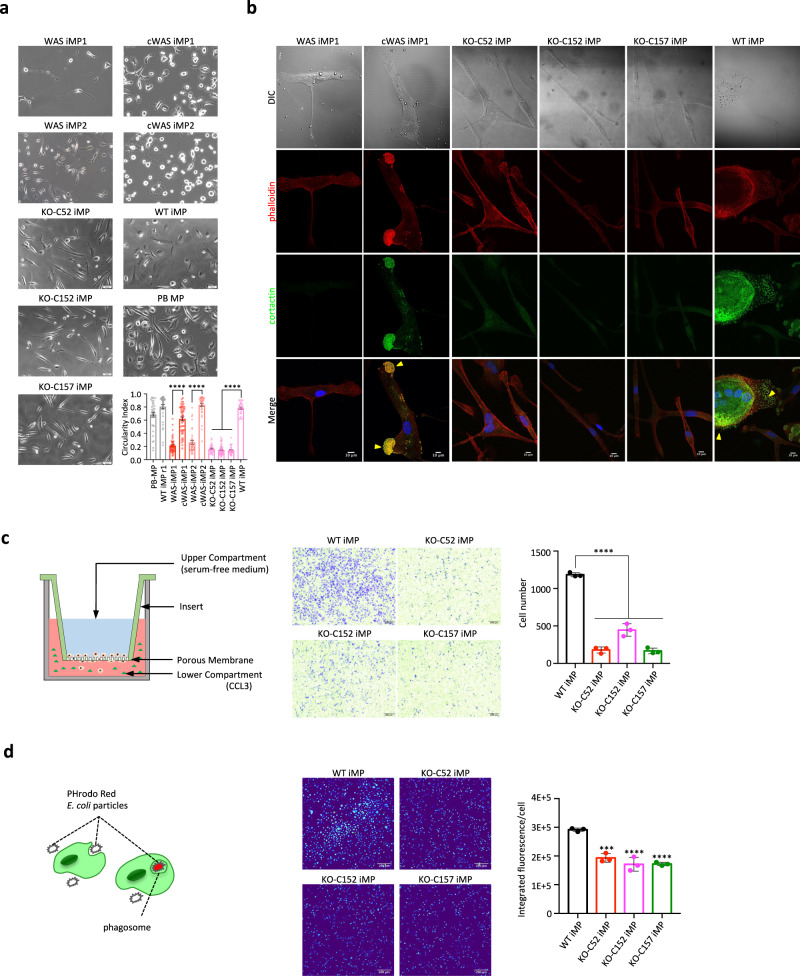
Recapitulation of WAS phenotypes using isogenic WAS-iPSC models. **a** Representative phase-contrast microscopy images of macrophages and quantitative analysis of their circularity. PB-MP: macrophages derived from normal peripheral blood. Analyzed cell *n* = 36 (PB-MP), 36 (WT iMP r1), 50 (WAS-iMP1), 50 (cWAS-iMP1), 36 (WAS-iMP2), 36 (cWAS-iMP2), 30 (KO-C52 iMP), 47 (KO-C152 iMP), 30 (KO-C157 iMP), and 28 (WT iMP). Data are shown as mean ± SEM, Two-sided Student’s *t* test, ****P < 0.0001. Scale bar = 50 μm. **b** Representative immunofluorescence images of podosomes (yellow arrowheads) in macrophages. Podosomes are marked by F-actin and cortactin (encoded by the *CTTN* gene). DNA was stained with DAPI. DIC differential interference contrast. Scale bar = 10 μm. The experiment was repeated three times independently. **c** Transwell migration assay. Left: schematic of the principle of the transwell migration assay. Each upper compartment was seeded with the same number of WT iMPs and KO-iMPs (three clones, three replicates). The images show macrophages that migrated to the underside of the porous membrane and were stained by crystal violet. Rightmost: quantitation of cell number in the images. Biological replicate *n* = 3. Individual dots represent cell number per sample, and the bars show mean ± SD, ****P = 0.0001 (one-way ANOVA). Scale bar = 200 μm. **d** Quantitative measurement of phagocytosis. Left: schematic of the principle of the phagocytosis assay using the pH-sensitive fluorescence dye pHrodo Red-conjugated *E. coli* particles. Right: fluorescence images and the quantification of pHrodo Red fluorescence in the same number of WT iMPs and KO-iMPs (three clones, three replicates). rightmost: quantitation of pHrodo Red fluorescence in each cell. Biological replicate *n* = 3, cell *n* = 2271 (WT iMP), 1653 (KO-C52 iMP), 1452 (KO-C152 iMP), 1180 (KO-C157 iMP). Data are shown as mean ± SD, ***P = 0.0001, ****P < 0.0001 (one-way ANOVA). Scale bar = 200 μm.

WAS-iPSCs expressed markers of the three germ layers upon spontaneous differentiation (Supplementary Fig. S2a). The expression of the *WAS* gene was reduced at the mRNA level in both patients, which was rescued by gene correction (Supplementary Fig. S2b, c). Both WAS-iPSCs and KO-iPSCs could be differentiated into CD34^+^ and CD43^+^ hematopoietic progenitor cells that form typical hematopoietic colonies (Supplementary Fig. S2d, e). We then differentiated KO-iPSCs into macrophages and WAS-iPSCs into macrophages and dendritic cells (DCs) to examine if they could recapitulate phenotypes observed in patients. Undifferentiated WAS-iPSCs and macrophages from both WAS-iPSCs or KO-iPSCs had no detectable WASP protein expression as compared to the isogenic controls (Fig. 1g). DCs (WAS-iDCs) derived from WAS-iPSCs, and macrophages derived from WAS-iPSCs and KO-iPSCs (WAS-iMPs and KO-WAS-iMPs) expressed typical lineage markers, consistent with clinical observations (Supplementary Fig. S2f–h)^19,35^. WAS-iMPs and KO-iMPs had an abnormal elongated morphology that is consistent with known defects in substrate interaction and spreading, as seen in patient macrophages (Fig. 2a)^35,36^. Podosome defects of WAS^36^ were highly penetrant in both patient WAS-iMPs and all three clones of KO-iMPs (Fig. 2b). Defective CD43 expression observed in patients^37–40^ was recapitulated in WAS-iMPs and KO-iMPs (Supplementary Fig. S2i). In addition, chemotaxis of WAS macrophages is known to be impaired^41,42^, which was recapitulated in KO-iMPs and WAS-iMPs (Fig. 2c and Supplementary Fig. S2j, k). Phagocytosis of *E. coli* particles was significantly impaired but not completely abolished in WAS-iMPs and KO-iMPs (Fig. 2d and Supplementary Fig. S2l), which was documented in patient monocytes and macrophages^43,44^. Importantly, all these phenotypes were corrected or absent in cWAS-iPSC or WT-iPSC-derived cells (Fig. 2 and Supplementary Fig. S2a–l). WASP and N-WASP share the highest homology among the members in WAS family^45^. N-WASP expression was similar at both the mRNA and protein levels between the WT iMPs and three KO-iMPs (Supplementary Fig. S2m), suggesting that N-WASP cannot account for the above-mentioned phenotypes. Together, these data from five iPSC lines in three independent genetic backgrounds strongly show that isogenic WAS models faithfully recapitulate known disease phenotypes and are suitable for studying pathogenic mechanisms of WAS.

### Splicing factors are overexpressed in WAS

**Fig. 3:**
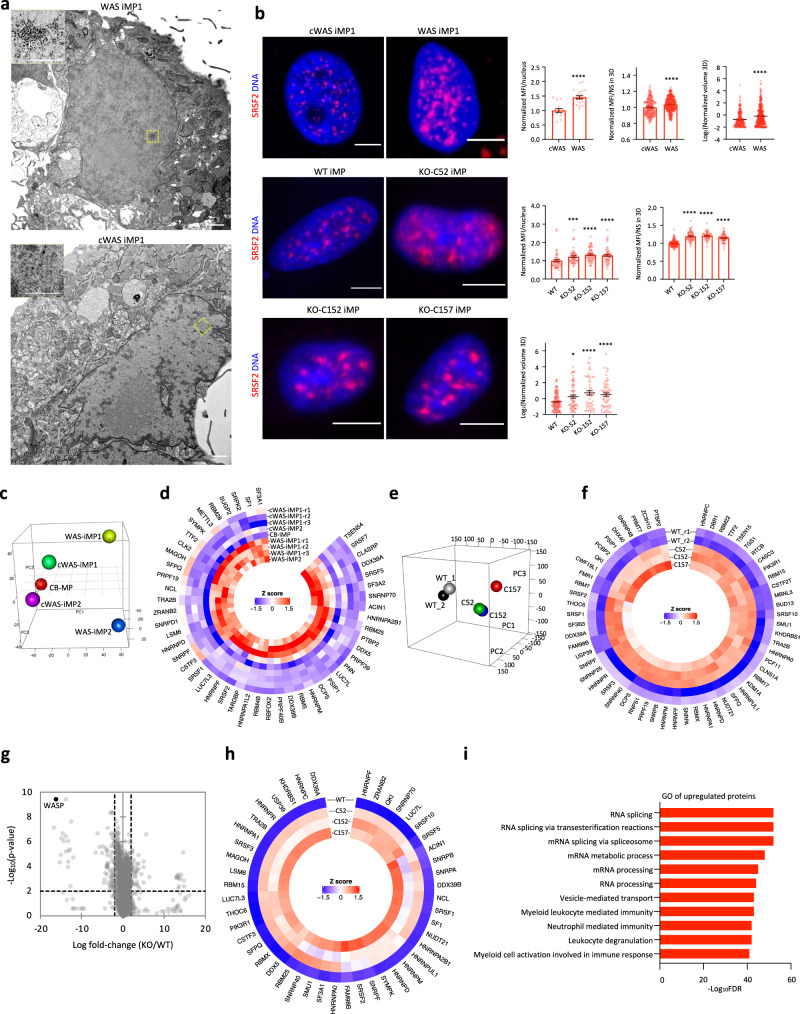
WASP deficiency results in overexpression of splicing factors. **a** Representative TEM images of 70-nm sections of WAS-iMPs and cWAS-iMPs collected under identical conditions. Bar = 1 μm in the large panels; Bar = 0.5 μm in the insets. IGC: interchromatin granule cluster (nuclear speckles). Number of cells analyzed: *n* = 7 (WAS-iMP1), *n* = 4 (cWAS-iMP1). **b** Representative maximum projection confocal images and image-based quantitative analysis of the nuclear speckle marker SRSF2 in WAS iMP1s, cWAS iMP1s, WT iMPs and KO-iMPs (three clones). The mean fluorescence intensity (MFI) of SRSF2 signal per nucleus was measured in the 2D images in cWAS iMP1s (*n* = 15), WAS iMP1s (*n* = 22), WT iMPs (*n* = 49), C52 KO-iMPs (*n* = 53), C152 KO-iMPs (*n* = 59), and C157 KO-iMPs (*n* = 54). For the 3D analysis of the MFI and volume of nuclear speckles, SRSF2 speckles were segmented and quantitated using the Avizo software (see “Methods”). Number of particles analyzed: *n* = 217 (cWAS iMP1s), *n* = 379 (WAS iMP1s), *n* = 129 (WT iMPs), *n* = 64 (C52 KO-iMPs), *n* = 51 (C152 KO-iMPs), *n* = 62 (C157 KO-iMPs). Data are normalized by the corresponding wild-type and shown as mean ± SEM. Comparisons were performed with two-sided Student’s *t* test, or Mann–Whitney test if data are not normally distributed, *P = 0.0173, ***P = 0.001, ****P < 0.0001. Bar = 5 μm. Source data are provided as a Source Data file. **c** PCA of RNA-seq data of WAS-iMPs, cWAS-iMPs derived from two patients, and wild-type CB-MP. **d** Heatmap of samples shown in C based on upregulated SFs. **e** PCA of RNA-seq data of WT iMPs and WASP-KO-iMPs. **f** Heatmap of upregulated SFs in WASP-KO-iMPs shown in (**e**). **g** Volcano plot of mass spectrometry of WT and WASP-KO-iMP samples. Parametric test with Benjamini–Hochberg correction. **h** Heatmap of upregulated SFs in WASP-KO-iMPs shown in (**g**). **i** GO analysis of upregulated proteins in WASP-KO-iMPs.

Consistent with previous reports^4,7^, WASP localized to both the cytoplasm and the nucleus in major blood lineages except for erythrocytes (Supplementary Fig. S3a–c). Although the roles of WASP in transcription have been described in T_H_ cells^4,6,7^, the nuclear localization and function of WASP in monocytes/macrophages have not been reported. Nuclear and cytoplasmic WASP staining in macrophages was verified with four different WASP antibodies (see “Methods”, Supplementary Fig. S3b). WASP puncta occupied interchromatin regions in blood cells (Supplementary Fig. S3b, c). WASP is known to co-localize with hyperphosphorylated RNA polymerase II (Pol II)^7^. However, whether WASP participates in other nuclear compartments remains unknown. We, therefore, examined the nuclear compartments using TEM analysis using WAS-iMPs (Fig. 3a) and WASP mutant B cells (Supplementary Fig. S4a–c). Mutant cells contained numerous prominent interchromatin granule clusters (IGCs) that correspond to nuclear speckles by negative staining in TEM^46^, whereas IGCs in gene-corrected or wild-type cells were inconspicuous (Fig. 3a and Supplementary Fig. S4c).

Nuclear speckles are dynamic structures enriched in SFs and involved in pre-mRNA splicing^46,47^. To confirm the nuclear speckle phenotype, we next performed immunofluorescence staining of nuclear speckle marker SRSF2. Consistent with the TEM data, patient iMPs, KO-iMPs, and WASP mutant B cells contained significantly more prominent and irregularly sized nuclear speckles compared to wild-type or gene-corrected cells (Fig. 3b and Supplementary Fig. S4d–f). The higher expression level of SRSF2 was confirmed by western blot (Supplementary Fig. S4g). Immunofluorescence staining of SF3B1, another nuclear speckle marker^48^, well recapitulated the nuclear speckle phenotype shown by SRSF2 immunofluorescence (Supplementary Fig. S4h). Thus, these data suggest that WASP regulates the expression of SRSF2 and the organization of nuclear speckles.

Nuclear speckles play key roles in transcription-linked biological processes^47,49^. We therefore examined gene expression in WASP mutant cells. We first performed RNA-seq of WAS-iMPs (four biological replicates), cWAS-iMPs (four biological replicates), and macrophages derived from normal cord blood (CB-MPs). Two patient WAS-iMPs differed from CB-MPs in distinctive ways in principal component analysis (PCA), yet both resembled normal macrophages after gene correction (Fig. 3c). There were 652 genes upregulated and 480 downregulated (FDR < 0.05). Consistent with the aforementioned nuclear speckle phenotypes, we found 49 nuclear speckle-associated SFs to be overexpressed in both patient iMPs (Fig. 3d). The enrichment of upregulated SFs in the upregulated genes is statistically highly significant (*P* < 3.517 × 10^−34^, hypergeometric test).

WAS patients display dysfunction, clonal proliferation, and malignant transformation of the innate and adaptive immune lineages^3^, we, therefore, analyzed primary T and B cells (despite our attempts, none of the samples produced enough macrophages for analysis) in cryopreserved peripheral blood mononuclear cells of WAS patients using RNA-seq (Supplementary Fig. S5a, b). PCA and hierarchical clustering of differentially expressed genes showed that the two patients (P1 and P2) differ from the controls in a distinct fashion (Supplementary Fig. S5b, c). T and B cells of P1 (who had a worse prognosis) showed much greater differences and formed a separate cluster outside of the T- or B-cell clusters (Supplementary Fig. S5c). Similarly, 126 and 92 RNA SFs were upregulated in T cells and B cells of WAS patients, respectively (Supplementary Fig. S5d, e).

The overexpression of SFs in WAS patient macrophages, T cells, and B cells suggested a possible role of WASP in RNA splicing. To test this hypothesis, we next performed ultra-deep RNA-seq in the KO-iMP and WASP mutant B-cell models. The three WASP-KO-iMP samples were transcriptomically distinct from WT iMPs (Fig. 3e). Fifty-eight SFs were upregulated in KO-iMPs, which overlapped the upregulated SFs in patient iMPs by 53.06% (Fig. 3d, f). In contrast, only six SFs were downregulated. The enrichment of upregulated SFs in the upregulated genes is statistically highly significant (*P* < 3.607 × 10^–22^, hypergeometric test). A similar observation was made in mutant B-cell lines (Supplementary Fig. S5f). To explore if transcriptional upregulation corresponded to elevated protein levels, we quantitated protein expression using the data-independent acquisition mass spectrometry (DIA-MS). As expected, the DIA-MS data showed that WASP was the most significantly downregulated protein in KO-iMPs (Fig. 3g). Consistent with the RNA-seq data, 124 RNA SFs were overexpressed at the protein level in KO-iMPs (Supplementary Data 1), of which 46 overlapped with those detected by RNA-seq in either patient iMPs or KO-iMPs (Fig. 3h). In contrast, only 8 SFs were downregulated in the DIA-MS data. RNA splicing-related processes were the most enriched gene ontology (GO) terms in the upregulated proteins in KO-iMPs (Fig. 3i). Taken together, the results showed that WASP deficiency causes an upregulation of RNA SFs at the mRNA and protein levels.

### Altered RNA splicing universally occurs in WASP deficiency cells

To investigate the effect of WASP deficiency on RNA splicing, we examined alternative splicing (AS) by comparing gene expression at the isoform level (see “Methods”). We identified 303, 310, and 726 genes with differentially expressed isoforms in WAS-iMPs, patient T and B cells, respectively (Supplementary Fig. S6a). Most of these were also differentially expressed at the gene level. About 16% of the isoforms were shared by at least two cell types (Supplementary Fig. S6a), and were highly enriched in GO categories including the nucleoplasm and RNA binding (Supplementary Fig. S6b). We confirmed the AS differences using an alternative bioinformatics pipeline (Supplementary Fig. S6c). Patient cells had a strong preference for exon promotion (higher inclusion) in AS events, and the genes with promoted exons identified in both B and T cells were enriched in protein and nucleic acid turnover (Supplementary Fig. S6d–f).

**Fig. 4:**
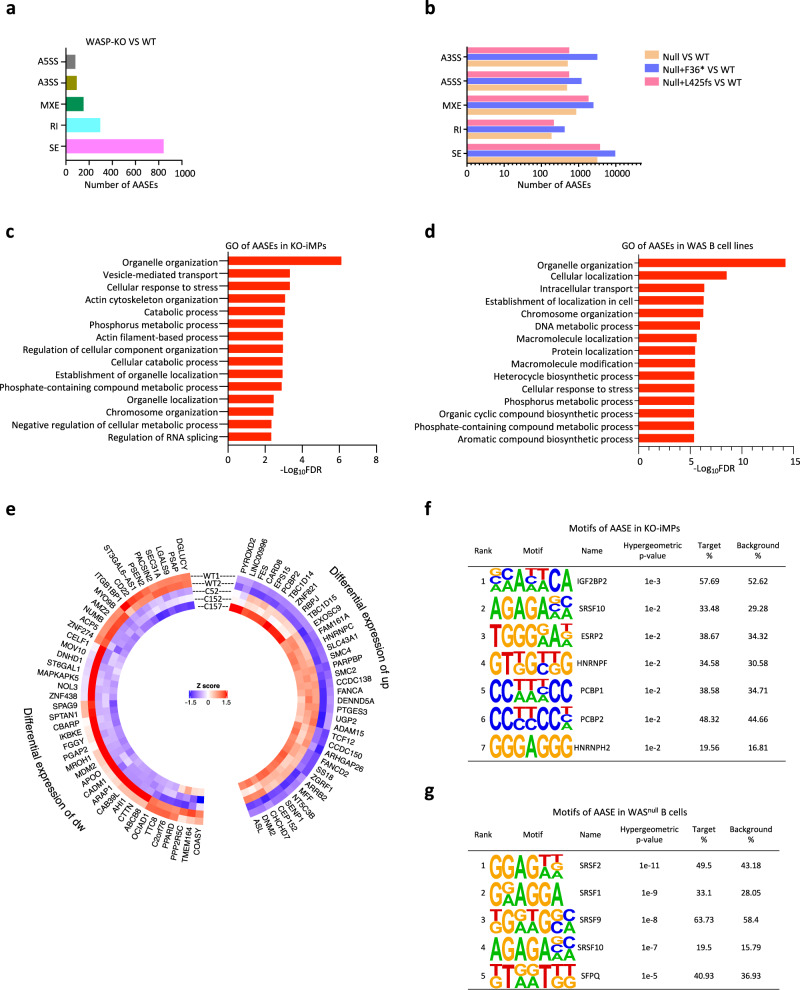
WASP deficiency results in altered RNA splicing in iMPs and B-cell lines. **a** Altered alternative splicing events (AASEs) in KO-iMPs (three clones). A3SS alternative 3’ splicing site, A5SS alternative 5’ splicing site, MXE multi-exon skipping, RI intron retention, SE exon skipping. WT iMP biological replicate *n* = 2, KO-iMP biological replicate *n* = 3. **b** AASEs in 3 WASP mutant B-cell lines. Biological replicate *n* = 2. **c** GO enrichment of AASEs in KO-iMPs. WT-iMP biological replicate *n* = 2, KO-iMP biological replicate *n* = 3. **d** GO enrichment of common AASEs in three WASP mutant B-cell lines. Biological replicate *n* = 2. Hypergeometric testing *P* values are corrected for multiple testing using a false discovery rate (FDR). **e** AASEs in both upregulated genes and downregulated genes in KO-iMPs. up upregulated genes, dw downregulated genes. **f** Splicing-factor motif analysis of altered SE, RI, and MXE events in KO-iMPs. **g** Splicing-factor motif analysis of the downstream exon of altered SE in *WAS*^*null*^ B cells.

**Fig. 5:**
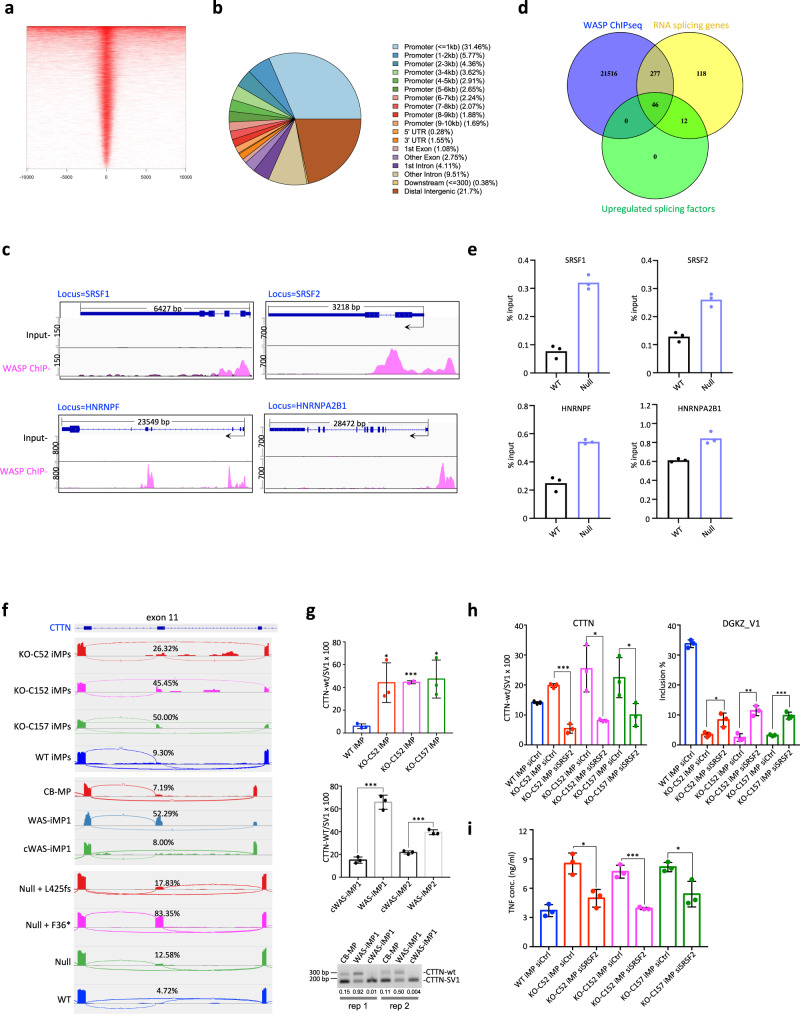
WASP binds to promoters of splicing-factor genes to regulate their transcription. **a** Heatmap of endogenous WASP ChIP-seq peaks around the TSS. **b** Genomic annotation of WASP ChIP-seq peaks. **c** WASP ChIP-seq peaks located in SF genes visualized in Integrative Genomics Viewer (IGV). **d** Venn diagram shows overlap between WASP-bound genes, RNA-splicing genes (GO term 0008380), and upregulated SFs of KO-iMPs. The hypergeometric testing *P* value is 1.27 × 10^−56^ for RNA-splicing term vs WASP ChIPseq and 5.21 × 10^−12^ for upregulated SF vs WAS ChIPseq. **e** Representative ChIP-qPCR results of the level of H3K27ac in WASP binding sites in selected SF genes. Biological replicates *n* = 2 for gene *SRSF1* and *HNRNPF*, and for gene *SRSF2* and *HNRNPA2B1* *n* = 3. One of the independent biological replicates is shown, and other biological replicate data are provided in a Source Data file. Data are shown as mean ± SEM. **f** Isoform-level analysis of RNA-seq data of macrophages and B-cell lines. The graph below the gene diagram shows IGV tracks of RNA-seq reads mapped to the *CTTN* gene. The numbers represent the percentage of exon 11-retained isoform in total isoforms. **g** Isoform-specific qRT-PCR analysis of two *CTTN* variants in macrophages. Bottom: Semi-quantitative RT-PCR shows expression of two *CTTN* isoforms in WAS-iMPs. Rep1 and Rep2: two biological replicates. The numbers below the gel pictures are densitometry ratio of CTTN-wt and CTTN-SV1. *P = 0.0196 (KO-C52 iMP), *P = 0.0131 (KO-C157 iMP), ***P = 0.0002 (KO-C152 iMP), ***P = 0.0002 (WAS-iMP1), ***P = 0.0003 (WAS-iMP2). **h** qRT-PCR analysis of the expression of the *CTTN* and *DGKZ* isoforms upon knockdown of SRSF2 in KO-iMPs. Biological replicate *n* = 3. *CTTN*: *P = 0.0181 (KO-C152 iMP), *P = 0.0487 (KO-C157 iMP), ***P = 0.0001; *DGKZ*_V1: *P = 0.0256, **P = 0.0017, ***P = 0.0007. **i** ELISA quantitation of TNF secretion by indicated macrophages upon stimulation with LPS. Biological replicate *n* = 3. *P = 0.0116 (KO-C52 iMP), *P = 0.026 (KO-C157 iMP), ***P = 0.0006. All statistics in this figure were done using a two-sided Student’s *t* test. Data in (**g**–**i**) are shown as mean ± SD.

To better quantitate the different types of AS events, we analyzed AS events using the ultra-deep RNA-seq data of KO-iMPs and B-cell lines. The results showed that skipped exon (SE) was the predominant form of AS change in KO-iMPs, followed by retained intron (RI), mutually exclusive exon (MXE), and alternative 3’ or 5’ splice site (A3SS and A5SS) (Fig. 4a). Similarly, alternative exon usage was the predominant form of AS change (SE and MXE in Fig. 4b, >75% on average) in mutant B cells (Fig. 4b). Many SE events were consistently detected in WAS-iMPs, KO-iMPs and WASP mutant B cells, including those in *CTTN*, *SENP1*, and *PCBP2* (Fig. 5f and Supplementary Fig. S6g). We validated these shared altered AS events (AASEs) of *CTTN*, *SENP1*, *TCF12*, *PCBP2*, *TPD52L2*, and *HMG20A* using exon-specific qPCR probes that distinguish the isoforms with the included or skipped exons (Fig. 5g and Supplementary Figs. S6h and S9c). We also validated AASEs that were specific to WASP-deficient macrophages in the *FN1*, *DGKZ*, and *HYAL2* genes, which are associated with immune functions (Supplementary Fig. S9a, b and Supplementary Data 2). Overall, the AASEs were enriched in biological processes such as organelle organization, chromosome organization, and cellular response to stress (Fig. 4c, d). The AASEs were present in both upregulated and downregulated genes (Fig. 4e). Motif analysis (see “Methods”) revealed that the cassette exons or downstream exons of AASEs contained multiple motifs that overlapped with binding sequences of human SFs that were upregulated in mutant cells (e.g., SRSF10, SRSF9, SRSF2, SFPQ, etc.) (Fig. 4f, g and Supplementary Fig. S6i, j). These findings suggest that aberrant overexpression of SFs (Fig. 3 and Supplementary Figs. S4 and 5) could be responsible for the AASEs in WASP-deficient cells.

### WASP binds to splicing-factor gene promoters and regulates local chromatin states

To understand if WASP directly participated in transcriptional regulation of SFs, we performed chromatin immunoprecipitation followed by high-throughput sequencing (ChIPseq) of endogenous WASP using a previously validated antibody^7^ in a wild-type B-cell line. We identified 80,711 high-confidence peaks that were strongly enriched at the transcription start site (TSS) (Fig. 5a). Major categories of WASP binding included proximal promoters (≤1 kb upstream of TSS,), distal promoters (between 1 and 10 kb upstream of TSS), distal intergenic DNA, introns, exons, 3’ UTR and 5’ UTR (Fig. 5b). WASP ChIP-seq peaks in the promoter and/or gene body of multiple genes were validated by ChIP-qPCR (Supplementary Fig. S8), and the genome-wide binding pattern of WASP was independently confirmed in *WAS*^null^ B cells overexpressing a wild-type WASP (Supplementary Fig. S7a, b, d).

WASP ChIP-seq peaks localized near the promoter of the SF genes that are misregulated in WAS (Fig. 5c and Supplementary Fig. S7c, d). The overlap between WASP-binding sites and RNA-splicing genes was statistically significant (Fig. 5d). For instance, 79% (46 out of 58) of the SFs upregulated in KO-iMPs were bound by WASP (Figs. 3f and 5c, d and Supplementary Fig. S7c, d). To understand the effect of WASP binding, we analyzed the active chromatin mark histone H3 lysine 27 acetylation (H3K27ac) in four SFs bound by WASP (Fig. 5c, d) and consistently upregulated in WAS mutant cells (Fig. 3d, f, h and Supplementary Data 1). We showed that the upregulation of SFs were correlated with a gain of H3K27ac, which is a marker of active transcription^50^, in WASP-bound promoter regions in *WAS*^null^ cells (Fig. 5e), suggesting that WASP regulates the local chromatin state of SF promoters. To explore the regulation role of WASP, we performed Homer de novo motif analysis using the promoter regions of upregulated SFs bound by WASP. Interestingly, three of the top-ranked motifs were best-matched to binding motifs of Kruppel-like zinc finger transcription factors (TFs) that contain the transcription repression KRAB domain (Supplementary Fig. S7e). The best-matched TFs included ZNF460 and ZNF189, which are associated with lymphoma and immunodeficiency, respectively. These data suggest that WASP might mediate transcriptional repression of SFs through KRAB-zinc finger TFs.

### Rescue of WAS-relevant phenotypes by genetic downregulation of splicing factors

Specific AS isoforms have been shown to be enriched in cancer due to their ability to promote proliferation and metastasis^51,52^. The full-length isoform of cortactin (CTTN-wt)^53^ is overexpressed in B-cell chronic lymphocytic leukemia but not normal B cells and correlates with poor prognosis^54^. RNA-seq and isoform-specific RT-PCR showed that CTTN-wt was specifically upregulated in WAS-iMPs, KO-iMPs and *WAS*^null^ B cells, whereas cWAS-iMPs, WT iMPs, CB-MP and WASP-rescued *WAS*^null^ B cells predominantly expressed a shorter isoform lacking exon 11 (CTTN-SV1)^53^ (Fig. 5f, g and Supplementary Fig. S9c). Moreover, overexpression of two WASP mutants resulted in a dramatic increase in CTTN-wt, suggesting that mutant WASP is responsible for the AS event (Supplementary Fig. S9c). Intriguingly, *CTTN* exon 11 contains a high density of the consensus exonic splicing enhancer motifs of SRSF2. The abundance of exon 11-containing transcript (CTTN-wt) correlated with the levels of SRSF2 (Supplementary Figs. S9c and S4g). Knocking down *SRSF2* in KO-iMPs and *WAS*^null^ and *WAS*^F36*^ B cells significantly reduced the ratio of CTTN-wt to CTTN-SV1 (Fig. 5h and Supplementary Fig. S9e), suggesting the overexpression of SRSF2 in WASP mutant cells is responsible for this AASE. Similarly, *SRSF2* knockdown partially rescued the AASE events in the *DGKZ* gene (Fig. 5h), which is involved in carcinogenesis and immune response^55,56^, while knocking down three other upregulated SFs (*SRSF1*, *SRSF3*, and *HNRNPD*) had no effects (Supplementary Fig. S9d). These data suggest that overexpression of SRSF2 may play a larger role than that of other SFs in AASEs.

A study in the *WAS* knockout mouse model and WAS patient cells showed that WASP-deficient macrophages upregulate the pro-inflammatory cytokine TNF, which was linked to exacerbated Th1/Th17-helper cell response and autoinflammatory colitis^57^. This pro-inflammatory phenotype was confirmed in our WAS models at the mRNA and protein levels (Supplementary Fig. S9f). While KO-iMPs secreted significantly more TNF than WT iMPs after treatment with lipopolysaccharide (LPS), this TNF overproduction phenotype was partially rescued by SRSF2 knockdown in all three KO-iMP lines (Fig. 5i).

### WASP physically interacts with splicing factors and regulates SRSF2 mobility

**Fig. 6:**
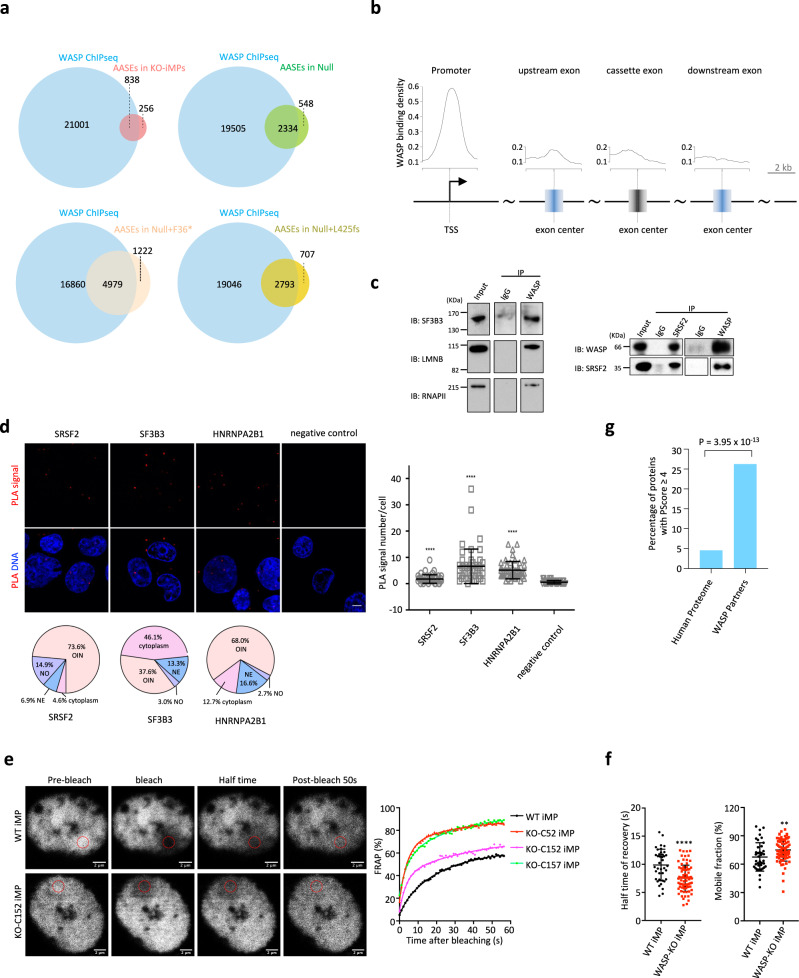
WASP directly binds alternatively spliced genes and physically interacts with splicing factors. **a** Venn diagram of WASP-bound genes and AASEs in WASP mutant cells. Hypergeometric testing *P* values are 1.39 × 10^−41^ (KO-iMPs), <10^−324^ (Null), <10^−324^ (Null + F36*), and <10^−324^(Null + L425fs). **b** WASP ChIP-seq signal density plotted around the center of the TSS and the cassette and flanking exons of all AASE genes identified in *WAS*^*null*^ B cells (shown in (**a**)). The vertical lines in the model gene diagram indicate the center of the elements. The distance from the center in kb is given by the scale bar. The *y* axes are drawn to the same scale. **c** co-IP validation of physical interaction between WASP and selected proteins. IP immunoprecipitation, IB immunoblotting. This experiment was done once for SF3B3, LMNB, and RNAPII, and twice for SRSF2. **d** Representative immunofluorescence images of PLA signals for WASP-SRSF2, WASP-SF3B3, and WASP-HNRNPA2B1 in the WT B-cell line. The pie chart below each image shows the percentage of PLA signals in different cellular compartments. Right: quantitative analysis of PLA signals. Cell number *n* = 50 for each setting. Data are shown as mean ± SEM. Mann–Whitney test, ****P < 0.0001. Nuclei were stained with DAPI. Bar = 5 μm. NE nuclear envelope, NO nucleolus, OIN other locations inside the nucleus. **e** Left: representative FRAP images of SRSF2-GFP expressing WT and WASP-KO-iMPs (KO-C152 is shown here, and other clones are shown in Supplementary Fig. S10i). Scale bar = 2 µm. Right: time-lapse FRAP curve of SRSF2-GFP fluorescence generated from representative iMP cells. **f** Statistic analysis for the half time of FRAP recovery and mobile fraction of SRSF2-GFP fluorescence in WT (*n* = 39) and WASP-KO-iMPs (*n* = 78). Data are shown as mean ± SD. Two-sided Student’s *t* test, **P = 0.004, ****P < 0.0001. Source data are provided as a Source Data file. **g** Percentage of predicted phase-separation proteins (PScore ≥ 4) in the human proteome (*n* = 18,476) and WASP partners among nuclear speckle proteins (*n* = 95). *P* value is calculated with Fisher’s exact test. Source data are provided as a Source Data file.

Surprisingly, WASP-binding sites were also significantly enriched in AASEs of WASP mutant cells (Fig. 6a), indicating that WASP could directly participate in RNA splicing. We further performed an integrated analysis of WASP ChIP-seq and AASEs and found that, besides the expected enrichment at promoters (Fig. 5a, b), WASP binding was also enriched around the cassette exon and its flanking exons, albeit to lower levels—especially for the downstream exon—than WASP binding at promoters (Fig. 6b). The exonic WASP binding was validated by ChIP-qPCR (Supplementary Fig. S8). These patterns of WASP binding suggest that WASP may work with SFs co-transcriptionally. To test this hypothesis, we first examined if WASP was present in the same complex as SFs. Only a few proteins are known to interact with WASP in the nucleus^6^. To systematically extend the knowledge on how WASP participates in nuclear functions such as splicing, we performed MudPIT (multidimensional protein identification technology) analysis using co-immunoprecipitation (co-IP) of endogenous WASP, which achieved specific and sensitive detection of WASP interaction partners (see “Methods”). MudPIT analysis in whole cells and purified nuclei (Supplementary Fig. S10a) identified 78 and 299 high-confidence WASP-interacting polypeptides, respectively. These included known partners of WASP, including SMARCA5 (mean spectral count (SC) 6, mean sequence coverage (SeqCov) 5.3%)^6^, WIPF1 (SC 17.5, SeqCov 9.7%)^4^, and VIM (SC 5, SeqCov 9.7%)^58^, thus validating our method (Supplementary Data 3). Analysis of proteomic datasets of nuclear sub-compartments^47,59,60^ showed that the 99 proteins of WASP interactome overlapped with components of nuclear speckle (e.g., SRSF2, SF3B3, and CDC5L, Supplementary Data 3), which are enriched in RNA-splicing-related GO terms (Supplementary Fig. S10b). Similarly, WASP-interacting proteins were highly enriched in RNA-binding proteins (Supplementary Fig. S10c)^61^.

The association between WASP and nuclear speckle components indicated an unexpected role of WASP in the RNA-splicing process. We thus confirmed the physical interaction between WASP and SFs that are bona fide nuclear speckle components using co-immunoprecipitation assay (Fig. 6c). Proximity ligation assay (PLA, see “Methods”), which is a sensitive and specific method for in situ detection of endogenous protein interactions, further showed that the locations where WASP physically interacts with SFs were consistent with known subcellular distribution of these factors (i.e., SRSF2: mainly nuclear^62^, SF3B3 (also known as SAP130) and HNRNPA2B1: shuttling between the nucleus and cytoplasm^63,64^) (Fig. 6d). Furthermore, purified recombinant WASP and SRSF2 proteins showed direct binding in the microscale thermophoresis (MST) assay (Supplementary Fig. S10f, g).

SRSF2 is shown to move dynamically between nuclear speckles and their surroundings^65^. We hypothesized that this process could be regulated through its interaction with WASP. To test this hypothesis, we expressed a GFP-tagged SRSF2 construct^65^ that had a similar nuclear distribution as the endogenous SRSF2 (Supplementary Fig. S10h) in WT and KO-iMPs and performed FRAP analysis. The FRAP data showed that the absence of WASP resulted in a faster fluorescence recovery and a higher mobile fraction of SRSF2. The difference between WT and KO-iMPs was statistically significant, although some clonal variation was observed (Fig. 6e, f and Supplementary Fig. S10i, j). Interestingly, the dynamics of SRSF2 within the nuclear speckles (i.e., SRSF2^bright^ speckles) was not significantly different between WT and KO-iMPs (Supplementary Fig. S10k), suggesting that the increase of SRSF2 dynamics was in the non-speckled regions. Besides, we used a non-speckle nuclear protein HP1α-GFP to evaluate if nuclear mobility was generally higher in WASP-KO cells. The results indicated that HP1α was highly immobile in both WT and KO-iMPs (Supplementary Fig. S10l). We confirmed that these observations were not artifacts as the same HP1α-GFP construct showed FRAP dynamics in HEK293T cell as documented in the literature^66^ (Supplementary Fig. S10l). These data supported the idea that WASP specifically alters SRSF2 mobility in the nucleoplasm. Endogenous WASP puncta were often observed near SRSF2^bright^ nuclear speckles (Supplementary Fig. S11a). Consistently, most WASP-SRSF2 interactions were detected near or outside of nuclear speckles (Supplementary Fig. S11b). Therefore, WASP physically interacts with the components of nuclear speckles including SRSF2 and constrains the mobility of SRSF2.

### Optogenetic WASP clusters with SRSF2, active Pol II, and newly transcribed RNA

**Fig. 7:**
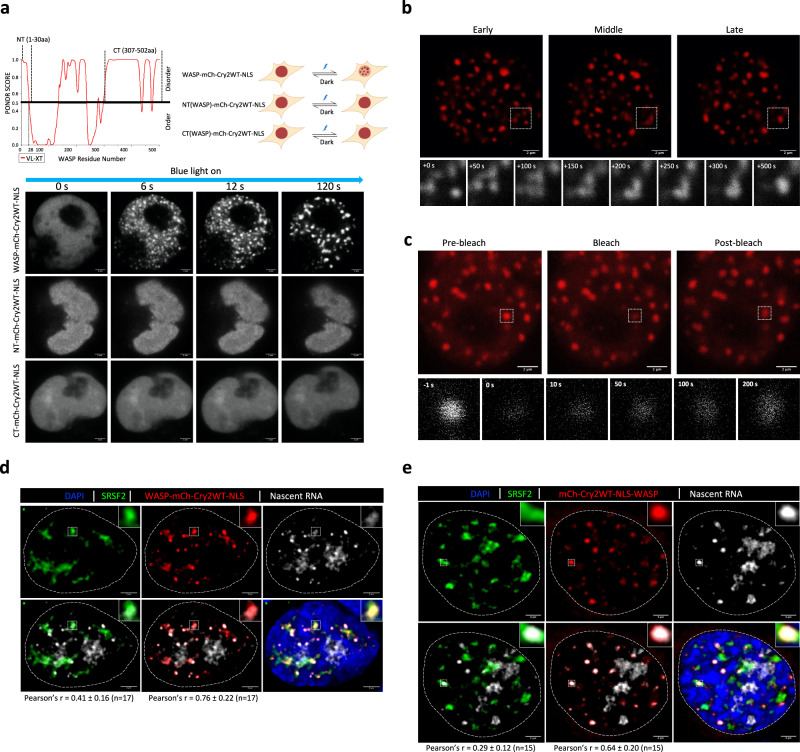
WASP forms condensates in the nucleus and clusters with SRSF2 and nascent RNA. **a** Top: schematic diagram of Cry2-based optogenetic constructs of WASP and its N-terminal (NT) and C-terminal (CT) IDRs (created with BioRender.com). The NT and CT locations are shown on the top of PONDR IDR prediction diagram. Bottom: time-lapse confocal images of HEK293 cells expressing photo-activated optogenetic constructs of WASP, NT, or CT following photoactivation. Scale bar = 2 µm. The experiment was repeated at least three times independently. **b** Confocal imaging of fusion of optoWASP optogenetic droplets after blue light activation. Time-lapse images of the region framed in the box are shown below. Scale bar = 2 µm. The experiment was repeated at least three times independently. **c** Confocal images showing fluorescence recovery after photobleaching (FRAP) of optoWASP droplets. Time-lapse images of the region framed in the box are shown below. Scale bar = 2 µm. The experiment was repeated at least three times independently. **d** Representative single-plane confocal images and colocalization analysis of optoWASP droplets (N-terminal WASP) with endogenous SRSF2 and nascent RNA. Insets show the framed region in the images. Scale bar = 2 µm. The Pearson correlation coefficient of colocalization (Pearson’s *r*) was calculated using the Leica LAS X software. Analyzed image *n* = 17, data are shown as mean ± SD. **e** Representative single-plane confocal images and colocalization analysis of optoWASP droplets (C-terminal WASP) with endogenous SRSF2 and nascent RNA. Insets show the framed region in the images. Scale bar = 2 µm. The Pearson’s r was calculated using the Leica LAS X software. Analyzed image *n* = 15, data are shown as mean ± SD.

Nuclear speckles are membraneless nuclear bodies with phase-separated, liquid-like properties that allow their constituents to shuttle between the nuclear body and nucleoplasm dynamically and transiently interact with nucleoplasmic factors^46,47,67^. These phase-separated condensates are driven by intrinsically disordered proteins (IDPs) that contain intrinsically disordered regions (IDRs)^12^. Intriguingly, WASP partners showed a strong enrichment for bioinformatically predicted^68,69^ IDPs (Fig. 6g, Supplementary Fig. S10d, e, and Supplementary Data 3), which implies a potential role of WASP in condensates. Because nuclear speckles in WASP mutant cells show an irregularly sized and sometimes reticular morphology rather than the more uniform and rounder speckle-like morphology in wild-type cells (Fig. 3b and Supplementary Fig. S4d, e), we hypothesized that WASP could control the localization of SFs (e.g., SRSF2, SF3B1) through LLPS. Indeed, WASP was predicted to contain multiple IDRs (http://www.pondr.com, Fig. 7a). To test if WASP could phase separate in vivo, we cloned the WT WASP and the predicted N- and C-terminal IDRs of WASP to the N-terminus of the Cry2-based optogenetic droplet construct^15^. Optogenetic WT WASP (optoWASP) readily formed LLPS droplets in cells, but neither IDR was sufficient for droplet formation (Fig. 7a and Supplementary Movies 1–3). Furthermore, we observed the growth and fusion of optoWASP droplets over time by live-cell imaging and the dynamic exchange of WASP molecules between the soluble and droplet phases by FRAP (Fig. 7a–c and Supplementary Movies 1 and 4), proving the liquid-like properties of optoWASP droplets.

WASP is a nucleation factor of actin polymerization. Using a GFP-tagged nuclear actin chromobody, we showed that nuclear filamentous actin (F-actin) colocalized with optoWASP droplets (Supplementary Fig. S10m), suggesting that optoWASP retained the normal function of WT WASP. We used 1,6-hexanediol (1,6-HD), one of the most widely accepted tools to disrupt condensates^70^, to investigate whether endogenous WASP participates in condensate formation. Untreated iMPs showed distinct WASP puncta similar to a well-known phase-separation protein, FUS^71^. However, most WASP puncta were significantly reduced in iMPs treated with 1% 1,6-HD for 20 min (Supplementary Fig. S10n). This 1,6-HD treatment did not affect cell viability. The puncta (condensates) of FUS condensates were similarly dissolved by this treatment, thus validating our method. These data demonstrated that endogenous WASP participates in phase separation.

A recent report^16^ showed that serum stimulation induces Pol II clustering, a process requiring N-WASP that phase-separated with Pol II. We therefore used the serum deprivation/stimulation paradigm to study optoWASP condensates. Several lines of evidence indicate the nuclear speckle as a storage depot of SFs that supplies SFs to active transcription sites nearby (reviewed in ref. ^72^). Nuclear speckles contain few, if any, genes within, and are frequently located close to active transcription loci that contain specific genes (e.g., coordinately expressed active genes)^46^. To probe if WASP regulates splicing by participating in the gene-body condensate that is the proposed site of active transcriptional elongation and co-transcriptional RNA processing, we fixed optoWASP-expressing cells after droplet induction and performed immunostaining of SRSF2 and CTD-phosphorylated actively elongating Pol II. We found that optoWASP droplets colocalized with a subset of SRSF2^dim^ regions that were adjacent to the SRSR2^bright^ nuclear speckles (storage sites of SRSF2). As expected, active Pol II also occupied territories adjacent to the SRSR2^bright^ nuclear speckles, some of which colocalized with WASP droplets and SRSF2^dim^ signals (Supplementary Fig. S11c, d). Endogenous WASP colocalized with a subset of active Pol II as described previously^7^ (Supplementary Fig. S11e). Consistently, most WASP-active Pol II interactions colocalized with active Pol II puncta (Supplementary Fig. S11b). These data show that WASP participates in phase-separated condensates encompassing SFs and active Pol II. Consistently, we found that optoWASP droplets could also cluster nascent RNA (produced by serum-stimulated transcription) together with SRSF2 (Fig. 7d, e), which further supported the role of WASP in RNA splicing. All observations made with N-terminal WASP-Cry2 fusion construct were reproduced with a C-terminal Cry2-WASP fusion construct (Fig. 7d, e and Supplementary Fig. S11c, d).

To confirm the interaction of WASP and RNA, we performed RNA immunoprecipitation followed by high-throughput sequencing (RIPseq). WASP antibody pulled down specific RNA species in WT but not in *WAS*^*null*^ B cells in either the whole cell or nuclear fraction (Supplementary Data 4). We further validated the enrichment of select RNA in WASP immunoprecipitation in wild-type cells using RIP-qPCR (Supplementary Fig. S11f). Taken together, our data reveal that WASP is a phase-separated protein and it could regulate RNA processing via interaction with SRSF2, active Pol II, and the nascent RNA in gene-body condensates.

## Discussion

WASP is a multifaceted protein important for diverse hematopoietic and immune processes^1^. The pathogenic mechanisms of WAS remain poorly defined, partly due to the lack of an experimental system of suitable for dissecting the function of WASP in appropriate cellular context in humans. Here, we take advantage of isogenic iPSC models to investigate the role of WASP in RNA splicing.

This study revealed that WASP deficiency caused by diverse WAS genotypes results in overexpression of SFs and leads to altered splicing events. We found a significant enrichment of WAS-associated AASEs in the immunity-associated gene list in the Gene Set Enrichment Analysis (GSEA) database (*P* < 0.002, Supplementary Data 2). Some of these AASEs affect genes that are not only associated with immunity but also play essential roles in inflammation (e.g., *FN1* and *HYAL2*) and cancer oncogenesis (e.g., *CTTN*, *FN1*, and *DGKZ*). The WAS-associated CTTN-wt isoform (Fig. 5g and Supplementary Fig. S9c) encodes an extra actin-binding domain compared to the CTTN-SV1 isoform. CTTN-wt is found to be overexpressed in B-cell chronic lymphocytic leukemia, but the functional relevance of the exon (i.e., the extra actin-binding domain) remains to be elucidated^54^. It has been reported that *HYAL2* has two variants that contain either exon 1 or exon 2 at the 5’ UTR. The splicing variant exons 1–3 is absent in cancer cell lines, but the variant exons 2–3 show a higher expression in cancer cell line^73^. In our model, exon 2 of *HYAL2* exhibited a higher inclusion ratio in WASP-KO-iMPs, which might be related to the high cancer susceptibility in WAS patients. In addition, exon 2 of *DGKZ* is more frequently skipped in WASP-KO-iMPs, leading to a truncated protein with two different alternative N-terminal domains (DGKZ_V1 and DGKZ_V2) with the same internal deletion (Met1-Ala243del). In summary, these new analyses show that WAS AASEs could lead to altered protein sequences and may be involved in disease-relevant processes.

We uncovered two mechanisms by which WASP regulates RNA splicing. First, WASP represses the transcription of SF genes under normal circumstances by directly binding to their promoters and regulating the local chromatin state. Second, we demonstrate that WASP is a phase-separated protein that can directly participate in the process of RNA splicing during transcription through physical association with target genes and components of the gene-body condensates (e.g., active Pol II, SFs, and nascent RNA) (Supplementary Fig. S12).

The evidence of WASP binding to splicing-factor promoters provides a hint that WASP regulates the transcription of SF genes. WASP could regulate chromatin remodeling and/or histone modifications of these promoters, which is supported by our observation of the gain of H3K27ac in mutant cells. Increasing evidence has shown that nuclear actin or nuclear actin polymerization regulates gene transcript by RNA Pol II^16,74–76^. It will be interesting to test if the transcriptional regulation of SFs by WASP depends on its actin-polymerization function. The nuclear actin phase separates with Pol II during serum-induced transcription, as process that requires N-WASP^16^. Another possibility is that WASP plays a role in regulating transcription initiation. Besides SFs, the identified WASP-binding partners include transcription factors and epigenetic regulators (Supplementary Data 3), which could be recruited by WASP (whose binding is enriched at TSS) to the gene promoter condensate that contains the pre-initiation complex, co-activators, and nuclear actin^17,77^ to regulate transcription. As WASP also occupies the gene body (i.e., exons and introns), an intriguing possibility that WASP may facilitate interactions between the promoter and distal sequences also warrants future study.

Recently, a WASP family protein, N-WASP (or WASL), was shown to drive LLPS via multivalent interactions with signaling complexes on the cell membrane^14^. Bioinformatics tools such as PScore^68^ and PSPredictor^78^ predicate that WASP has phase-separation propensity, but no experimental evidence existed prior to this study. We proved WASP is a phase-separated protein in the nucleus by using an optogenetic tool and by disrupting endogenous WASP condensates with 1,6-HD. WASP in the liquid-droplet phase carries out normal function in actin nucleation (Fig. 7a and Supplementary Fig. S10m). However, the N- and C-terminal IDRs of WASP are not sufficient for liquid–liquid phase separation (Fig. 7a), suggesting that multiple IDRs are involved in LLPS of WASP. Our data revealed extensive protein–protein interactions that WASP mediates in the nuclear compartment, including interactions with numerous SFs. We showed that WASP in normal situations constrains the mobility of SRSF2. Interestingly, the nuclear speckles are enlarged and irregularly sized in WASP-deficient cells (Fig. 3b). The nuclear speckle morphology can be disturbed by carcinogenesis, viral infection, and phosphorylation of serine/arginine-rich SFs and assumes an enlarged and irregular appearance^46,47,79^. This is similar to the phenotype of WASP-deficient cells (Fig. 3a, b and Supplementary Fig. S4c–e, h). In addition, we showed WASP droplets encompass SRSF2, active Pol II, and the nascent RNA. It is thus conceivable that WASP may facilitate the function of the splicing machinery in the splicing condensate described recently^17,77^ through LLPS in the nucleus. Future work is needed to decipher the relative contribution of the two mechanisms and how they coordinate splicing regulation.

The cellular models here presented can be used for elucidating how the mechanisms and protein factors identified herein contribute to pathologies of WAS. Finally, the significant alterations in gene expression and AS described in this study may serve as diagnostic biomarkers for WAS patients and constitute the basis upon which to develop targeted therapies for WAS.

## Methods

### Cells used and culture conditions

The use of human samples that were obtained with informed consent was approved by the Institutional Review Board (IRB) of the Salk Institute for Biological Studies and KAUST Institutional Biosafety and Bioethics Committee (IBEC). Human fibroblasts from a male Wiskott–Aldrich syndrome patient with the c.1271dupG mutation (in the *WAS* gene were purchased from the Coriell Institute (Camden, NJ). Human fibroblasts from a male Wiskott–Aldrich syndrome patient with the c.107_108del mutation and from the mother of the patient (unaffected) were purchased from the Cell Line and DNA Bank from Patients Affected by Genetic Diseases (Genova, Italy). All human fibroblasts were cultured at 37 °C in DMEM containing GlutaMAX, nonessential amino acids, sodium pyruvate, and 15% fetal bovine serum (FBS). The B-lymphocyte cell lines ID00003 (*WAS*^*null*^*:* c.431 G > A, p.E133K, clinical score 4–5) and GM11518 (wild type) were purchased from the Coriell Institute (Camden, NJ). All lymphoblastoid cell lines were cultured at 37 °C in RPMI-1640 containing 2mM L-glutamine and 15% FBS. H1 and H9 human ESCs were purchased from WiCell Research. Human ESCs and generated iPSC lines were cultured on Matrigel or mitotically inactivated MEF cells at 37 °C in DMEM/F12 (Invitrogen) supplemented with 0.1 mM nonessential amino acids (Invitrogen), 1 mM GlutaMAX (Invitrogen), 20% Knockout Serum Replacement (Invitrogen), 55 mM beta-mercaptoethanol (Invitrogen) and 10 ng/ml bFGF (Joint Protein Central) as described^80^. Peripheral blood mononuclear cells from two WAS patients were obtained with informed consent under approval by IRB at IRCCS Fondazione Policlinico San Matteo. One patient (P1, clinical score 4) manifested thrombocytopenia, eczema that was difficult to control, infections, humoral immune deficiency, but no autoimmunity or cancer. P1 was diagnosed with WAS over 25 years ago, and no genetic test was done. The other patient (P2, clinical score 2) manifested thrombocytopenia and humoral immune deficiency, but no autoimmunity or cancer. P2 carries the mutation p.P413fs, which impacts the c-terminal VCA domain of WASP. All cells of P1 and P2 were used to produce the data presented in the manuscript and therefore are no longer available. Human buffy coats were purchased from San Diego Blood Bank (San Diego, CA). Human umbilical cord blood units were gifts from StemCyte (Covina, CA) and San Diego Blood Bank (San Diego, CA). The cord blood units were anonymized and donated to research by individuals who gave informed consent. Cord blood CD34-positive cells were isolated by immune selection using human CD34 magnetic microbeads and MiniMACS separator (Miltenyi Biotec, San Diego, CA) according to the manufacturer’s instructions. The purity of the cells was checked by staining with anti-CD34-PE antibody on a BD LSRFortessa cytometer. Cryopreserved CB CD34^+^ cells were FACS sorted again before being used in experiments. The HEK293T cell line was purchased from ATCC and cultured in DMEM medium containing 10% FBS (heat-inactivated).

### iPSCs generation

The episomal vector-based reprogramming was performed as in refs. ^25,26^. Briefly, fibroblasts were nucleofected with the episomal vectors using a Nucleofector II (Lonza) with program U20. Five days after nucleofection, fibroblasts were reseeded onto mitotically inactivated mouse embryonic fibroblasts (MEFs). The reprogrammed colonies were allowed to grow until they were ready to be mechanically picked and transferred onto MEFs. Upon successful expansion of the iPSC lines on MEFs, they were then cultured on Matrigel (BD Biosciences) in mTeSR medium (StemCell Technologies). Analyses of pluripotency of iPSCs, including detection of transgene insertion, transgene expression, teratoma formation, immunostaining of pluripotency markers, and karyotyping were carried out as in refs. ^25,26^. The genotype of the WAS-iPSCs was periodically verified during the course of this study. The identity of the iPSCs was checked by Sanger sequencing and DNA fingerprinting.

### Construction and preparation of HDAdV

Construction and preparation of WAS-c-HDAdV for gene correction were generated using a BAC clone containing the human *WAS* locus (RP11-1148L6, BACPAC Resources)^81^. In brief, an *FRT*-PGK-EM7-neo-bpA-*FRT* fragment was recombined into intron 2 of *WAS* in the BAC clone. A total of 20.4 kb of *WAS* homology sequence and *FRT*-PGK-EM7-neo-bpA-*FRT* cassette were subcloned into the HDAdV plasmid pCIHDAdGT8-4 (kindly provided by Dr. Kohnosuke Mitani) (Suzuki et al.^32^). The WAS-c-HDAdV virus was generated according to a published method^32,82^. Briefly, the WAS-c-HDAdV plasmid was linearized by PI-SceI (New England Biolabs) and transfected into 116 cells (a kind gift from Dr. Philip Ng) in the presence of helper virus AdHPBGF35 (a kind gift from Dr. André M. Lieber). Crude virus extracts were serially amplified in 116 cells and the purified. βgal-transducing units (btu) were determined in 293 cells to define infectious vector titers.

### Isolation of gene-corrected human iPSCs

For the generation of gene-corrected iPSCs, 2.5–3.0 × 10^6^ *WAS* patient iPSCs were infected with *WAS*-c-HDAdV at the multiplicity of infection (MOI) of 3-30 btu/cell. Two days after infection, G418 (50 μg/ml; Invitrogen) was added to the medium to start positive selection. After 9 days, 2 μM Ganciclovir (GANC; Invitrogen) in addition to G418 was added to the medium to start negative selection. After an additional 8 days, G418/GANC double-resistant clones were transferred to 96-well plates and expanded for further characterization. Gene-targeted clones were determined by PCR of genomic DNA from drug-resistant clones with the following primers (P1, 5’-CATCCTGCCCCAGCCGACCAGACCTTAATGCTC-3’; P2, 5’-CCCCAAAGGCCTACCCGCTTCCATTGCTCA-3’; P3, 5’-CTACCTGCCCATTCGACCACCAAGCGAAACATC-3’; P4, 5’- AAATTTTCCGCCATCTTTCCCCACGGCTAACGAC-3’; see Fig. 1e) using PrimeSTAR GXL DNA Polymerase (TAKARA). To determine gene-corrected clones for exon 1, exon 1 of *WAS* was PCR-amplified with the following primers: 5’-CCACCCAGGCCCATGACTACTCCTTGCCACA-3’ and 5’- AGGCAACCATCCCGGCTGAGAGAATACCCA-3’ with PrimeSTAR GXL DNA Polymerase. To determine gene-corrected clones for exon 10, exon 10 of *WAS* was PCR-amplified with the following primers: 5’-ACTGCTTCAGTCAGGAGTTG-3’ and 5’-GAGGCTGACACAAGATTCAT-3’ with PrimeSTAR GXL DNA Polymerase. Amplicons were sequenced using an ABI 3730 sequencer (Applied Biosystems). The inserted neomycin-resistance cassette was removed using the FLP/*FRT* system^80^.

### Knockout iPSC generation

We used a previously well-characterized wild-type iPSC line^26,33^ (46XX) to perform homozygous knockout of the *WAS* gene using CRISPR-Cas9. To knock out the entire *WAS* gene, sgRNAs targeting upstream and downstream of the *WAS* gene with the following protospacer and protospacer-adjacent motif (PAM) sequences–5’-CCAAGCTCAGCCTAACGAGG-AGG-3’ (upstream) and 5’-GGATTTCACCCCCTAAGGGC-AGG-3’ (downstream)–were synthesized by in vitro transcription using the T7 MEGAshortscript kit (Thermo Fisher Scientific). Neon transfection system (Thermo Fisher Scientific; 1600 v/10 ms/3 pulses) was used for electroporating 200 k single cells with 50 pmol Cas9 protein and 25 pmol each sgRNA ribonucleoprotein complexes (RNPs) in buffer R (provided by Neon kit). After electroporation, iPSCs were cultured in Essential 8 medium (Thermo Fisher Scientific) with ROCKi (Abcam) on rhLaminin521 (Thermo Fisher Scientific) coated 24-well plates. The cells were dissociated by TrypleE after 48 h and subsequently 1000 single cells were seeded on laminin521 coated six-well plates. After 7 days half of the colony was picked for genotyping by PCR using the following primers: (F1, 5’-GTAGTAACCCTTCCGGACTA-3’ and R1, 5’-CGTAAAGGCGGATGAAGTAG-3’; F2, 5’-TCACACTCACCCAACAATCC-3’ and R2, 5’-TAACTGGGCCGACATTTCTC-3’, see Fig. 1d). The genotyping results were confirmed by Sanger sequencing.

### Plasmid construction

The FLAG-tagged WASP, WASP^F36*^, WASP^ΔEX2^, and WASP^L425fs^ were cloned into the pLenti-CAG-PL9 (kind gift from the Drs. Inder Verma and Ronald Evans) using the Gibson Assembly® HiFi 1-Step Kit (Cat# GA1100-10, San Diego, Synthetic Genomics, CA). The pHR-mCh-Cry2WT was purchased from Addgene (Cat# 101221). The SRSF2-GFP plasmid was purchased from OriGene (Cat# RG209842). The nuclear actin-Chromobody plasmid (TagGFP2) was purchased from Chromotek (Cat# Acg-n). The HP1α-GFP was purchased from Addgene (Cat# 17652). A nuclear localization signal (NLS) was added to C-terminal of Cry2WT in pHR-mCh-Cry2WT. N- and C-terminus IDRs of WAS were cloned to the N-terminal side of mCherry in the pHR-mCh-Cry2WT-NLS plasmid, and the WASP coding sequence was cloned to either the N-terminal side of mCherry or the C-terminal side of Cry2WT-NLS in the pHR-mCh-Cry2WT-NLS plasmid. All the constructs generated were subjected to DNA sequencing to confirm accurate sequences.

### Lentivirus production

shRNA lentiviral vectors targeting SRSF2 (TRCN0000000106 and TRCN0000000109) were purchased from the Mission shRNA library of Sigma-Aldrich. Lentiviruses were generated by co-transfecting the 293 T cells (ATCC) with the lentiviral vectors together with the packaging plasmids (pMDLg/pRRE, pRSV-Rev, and pMD2.G, from Addgene, 12251, 12253, and 12259, respectively) into 293T cells using Lipofectamine 3000 (Cat# L3000-015, Invitrogen). Lentiviruses were collected 24 h after transfection, filtered through a 0.45-mM filter, and concentrated using the PEG-it virus precipitation solution (SBI).

### Fluorescence in situ hybridization

The WAS-c-HDAdV probe was custom labeled with 5-TAMRA by Empire Genomics (Buffalo, NY). 5-fluorescein-labeled chromosome 11 probe was purchased from Empire Genomics. Cells were fixed in 4% formaldehyde for 10 min at room temperature, permeabilized with Triton X-100 and repeated freezing in liquid nitrogen, and deproteinized by treating with 0.1 M HCl. Hybridization was carried out at 37 °C overnight. Samples were counterstained with DAPI, mounted, and examined using a Zeiss LSM 780 Laser Scanning Confocal Microscope.

### Hematopoietic differentiation

Hematopoietic differentiation efficiency and hematopoietic colony formation activity were assayed as described in ref. ^83^ with some modifications. Briefly, iPSCs and ESCs cultured on MEF feeders in human ESC medium were manually dissociated and plated on OP9 feeders to start differentiation. Cells were co-cultured with the OP9 feeders for 12–14 days in OP9 differentiation medium (Alpha MEM containing 10% Hyclone defined FBS (GE Life Sciences catalog number SH30070.03), 100 μM monothioglycerol (Cat# M6145, Sigma-Aldrich) and 50 μg/ml ascorbic acid (Cat# A4544, Sigma-Aldrich). Differentiated cells were dissociated with collagenase and Accutase (Innovative Cell Technologies) and subjected to flow cytometry analyses, cell sorting, qPCR analyses and clonogenic progenitor cell assays. Macrophage and dendritic cell differentiation were performed as described^84^. Briefly, 2-week differentiated hiPSC were harvested and the non-adherent fraction (or FACS sorted CD34^+^/CD43^+^) were cultured under myeloid progenitor expansion condition (IMDM 10% FBS, GM-CSF (100 ng/ml, Peprotech cat# 300-03), M-CSF (50 ng/ml, Peprotech cat# 300-25)) for 10 days and then induced to maturity by lineage-specific cytokines. Macrophages were matured in X-vivo 10 (Lonza Cat#BE04-380Q) supplemented with 50 ng/ml M-CSF for 1 week. DC differentiation was induced using DC differentiation medium (RPMI-1640 plus 10% FBS, GM-CSF (100 ng/ml), IL4 (10 ng/ml, Peprotech)). DCs were matured in RPMI-1640 supplemented with 10% FBS, 10 ng/ml TNFα, 10 ng/ml IL4, and 3 μg/ml LPS (Sigma-Aldrich, Cat# L3024-5MG).

### Characterization of iPSC-derived cells

Characterization of the identity of iMPs and iDCs using lineage-specific cell surface markers were performed as described in a published protocol^85^. Briefly, 100 K cells were washed in FACS buffer (1× PBS without Ca^2+^ or Mg^2+^, 2 mM EDTA, 2% FBS) and pelleted at 400×*g* for 5 min at 4 °C. The pellet was resuspended in 100 μl FACS buffer in a 5-ml polystyrene test tube. Antibodies or isotype controls were added to the cell suspension and incubated for 30 min at 4 °C with agitation. Four milliliters of FACS buffer were added to wash the cells. The washed cells were pelleted at 400×*g* for 5 min at 4 °C. The pelleted cells were resuspended in FACS buffer without FBS and analyzed on a flow cytometer. Macrophage morphology was quantitated using the circularity plugin of ImageJ. Macrophage phagocytosis assay was performed with pHrodo™ Red *E. coli* BioParticles® Conjugate for Phagocytosis (Cat# P35361, Life Technologies, Carlsbad, CA). The pHrodo red particles were prepared according to the manual and opsonized *Escherichia coli* BioParticles® opsonizing Reagent (Life Technologies, Carlsbad, CA) as instructed. Adherent macrophages were incubated with pHrodo red *E. coli* particles for 1 h at 37 °C, dissociated, washed, and analyzed by FACS. Migration assay was performed using the CytoSelect™ 24-Well Cell Migration Assay, 5 µm kit (Cat# CBA-102, Cell Biolabs, inc., San Diego, CA), or using 24-well Transwell plates with 5-µm pore inserts (Corning, Inc., Maine, USA) as follows: 50,000 macrophages were resuspended in 100 µl X-Vivo-10 serum-free medium (Lonza, Cat#BE04-380Q) with 30 µM R848 (Stem Cell Technologies) and seeded in the insert; IMDM medium (Thermo Fisher Scientific) with 10% FBS and 5 ng/ml CCL3 was added in the lower compartment; The cells of the insert were stained by crystal violet (BD) after 12 h of incubation at 37 °C, 5% CO_2_ and the cells on the upper surface of the insert were removed by cotton swabs; the number of migratory cells was measured using the imageJ software (ver. 1.51 s).

### Clonogenic progenitor cell assay

Hematopoietic clonogenic assays were performed in 35-mm low-adherent plastic dishes (Stem Cell Technologies) in triplicate using 1.1 ml/dish of MethoCult GF + H4435 semisolid medium (Cat# 04435, Stem Cell Technologies) or StemMACS human HSC-CFU complete medium with Epo (Cat# 130-091-280, Miltenyi Biotec). Colony-forming cells (CFCs) were scored after 15 days of incubation. Cytospins were stained with the Diff stain kit (IMEB K7128) according to the manufacturer’s instructions.

### qRT-PCR

Total RNA extraction and cDNA synthesis were performed with the RNeasy micro kit (Qiagen) and iScript™ Reverse Transcription Supermix for RT-qPCR (Cat# 1708840, Biorad). Quantitative RT-PCR was carried out with SsoAdvanced™ Universal SYBR® Green Supermix (Cat# 1725270, Biorad) or PrimeTime® Gene Expression Master Mix (Cat# 1055770, IDT) coupled with fluorescently labeled probes on a CFX384 real-time PCR detection system (Biorad). The oligonucleotides used in this paper are shown in Supplementary Data 5.

### Live-cell imaging

The Cry2 fusion constructs were transfected into HEK293 cells using Lipofectamine 3000. After 24 h, the HEK293 cells were seeded in eight-well µ-slides with a glass bottom (Ibidi). All live-cell imaging was performed using ×93 glycerol objective on a Leica SP8 confocal microscope equipped with a chambered stage maintaining a humid environment at 37 °C, 5% CO_2_. Generally, cells were activated by a laser with a wavelength of 488 nm and imaged by a laser with a wavelength of 580 nm.

### Serum starvation and stimulation

Before serum starvation, the HEK293 cells were transfected with the Cry2 fusion constructs using Lipofectamine 3000 and cultured in DMEM medium with 10% FBS for 24 h. Subsequently, cells were harvested and seeded in eight-chamber slides in DMEM medium without FBS overnight. The medium was replaced by 20% FBS DMEM medium for one hour (for nascent RNA labeling, the 20% FBS medium was mixed with 1 mM 5-ethynyl uridine (Thermo Fisher, cat # C10329)). The cells were exposed under blue light for 10 min to induce optogenetic droplet formation and immediately fixed by 4% formaldehyde in PBS for 20 min at room temperature to be ready for the further staining experiment. We used a Click-it RNA imaging kit (Thermo Fisher, cat # C10329) to stain nascent RNA followed a standard protocol provided by the kit.

### Fluorescence recovery after photobleaching assay

FRAP assay of optoWASP was performed on a Leica SP8 confocal equipped with a live-imaging chambered stage. The optoWASP positive cells were activated by 488 nm laser and monitored by 588 nm laser in a live mode every 5 s until the droplets reached proper sizes. The photobleaching applied 588 nm laser with 100% power for 20 iterations, for a circular region with ~1.0 µm in diameter containing a droplet inside. Images were acquired for five prebleach frames with 2 s interval time and 30 postbleach frames with 10-s interval time. For the FRAP assay of SRSF2-GFP and HP1α-GFP, 0.5 μg plasmid were delivered to 1 × 10^5^ WT iMP or WASP-KO-iMPs using the Neon transfection system (Thermo Scientific). The electroporation parameters are as follow: pulse voltage 1900 v; pulse width 30 ms; pulse number 1. Cells were cultured for 24 h before imaging. The same amount of HP1α-GFP was transferred into HEK293T cells using Lipofectamine 3000. FRAP was performed on ZEISS LSM 710 inverted confocal microscope. Pixel dwell is 0.42 microseconds for SRSF2-GFP and 0.67 microseconds for HP1α-GFP. Bleaching was carried out for 50 iterations with 100% 405-nm laser. Images were collected every 1.04 microseconds for SRSF2-GFP and every 1 s for HP1α-GFP. After image acquisition, the data were analyzed using FRAP module installed in ZEISS software. The t-half and mobile fraction were used for statistical analysis and the time-lapse data were used for generating the FRAP curve.

### Immunofluorescence analysis

Cells were fixed with 4% formaldehyde in PBS for 20 min at room temperature, and then permeabilized with 0.4% Triton X-100. After a blocking step with 10% normal donkey serum in PBS, cells were incubated with the primary antibody at 4 °C overnight, followed by incubation at room temperature with the corresponding secondary antibody for 1 h. Nuclei were counterstained with 4’,6-diamidino-2-phenylindole (DAPI) and mounted with Vectashield (Vector Laboratories). For condensate disruption, cells were treated with 1% 1,6-HD (AAA1243936, Thermo Scientific) for 20 min followed by fixation and permeabilization. Images were acquired with a ZEISS LSM 780 laser-scanning inverted confocal microscope or Leica SP8 TCS STED 3X microscope. Afterward, we used the Avizo 2021.1 software to analyze the 3D information of the captured nuclear speckles. Specifically, we used the interactive thresholding module to segment the nuclear speckles. Then, we used the mean and volume3d functions from the label analysis module to calculate their mean fluorescence intensity and volume.

### Colocalization analysis of optoWASP confocal images

The proteins were labeled by excitation wavelength well-separated dyes of Alexa Fluor 488, mCherry, and Alexa Fluor 647, and the high-resolution images were collected by a Leica SP8 TCS STED ×3 microscope using the lightning mode with the lowest channel cross-talk setting. The colocalization was analyzed by the Leica LAS X microscope software. The whole single cell was circled as a region of interest (ROI) for colocalization analysis of optoWASP and SRSF2 or Pol II. The nucleoli where rRNA are highly expressed but without visible optoWASP expression were not included in ROIs for colocalization analysis of optoWASP and nascent RNA. The Pearson correlation coefficient (Pearson’s *r*) value was calculated within the ROIs.

### Transmission electron microscopy

Cells grown on coverslips were fixed for 5 min at 37 °C and then transferred to 4 °C and brought to the Salk Biophotonics core facility for further processing. Media were removed and washed with warmed buffer before the fixative solution (2.5% glutaraldehyde + 2% paraformaldehyde + 2 mM CaCl_2_ in 0.15 M cacodylate buffer). Cells were washed and fixed again (1% OsO_4_ and 0.3% potassium ferrocyanide in 0.15 M cacodylate buffer, 2 mM CaCl_2_) and for 1 h on ice in the dark. After washing, cells were stained with 2% uranyl acetate for one hour on ice in the dark. The cells were then washed in distilled water and acetone or ethanol series. Embedding was done with Spurr’s embedding kit. After cleaning with methanol, the slides were coated with Teflon and let dry. The embedded samples were flattened using two glass slides in resin. The samples were imaged using a Carl Zeiss Libra 120 kV PLUS Energy Filtered Transmission Electron Microscope that is equipped with a 2k × 2k fiber optically coupled YAG CCD camera.

### Proximity ligation assay and colocalization analysis

Proximity ligation assay (PLA) is a method that allows in situ detection of protein interactions with high sensitivity and specificity. Briefly, two primary antibodies raised in different species are used to label the target proteins (e.g., WASP and SRSF2). PLA probes, which are species-specific secondary antibodies conjugated with a short oligonucleotide sequence, bind to the primary antibodies. When the target proteins are in proximity (<30–40 nm), the oligonucleotides of the two different PLA probes can participate in rolling circle amplification. The amplified DNA can then be visualized by a fluorescent oligonucleotide probe, generating strong fluorescence signals (e.g., red spots in confocal images). These red PLA spots indicated the locations where WASP interacts with its partner proteins. PLA was carried out according to the manufacturer’s protocol for the Duolink® In Situ Red Starter Kit Mouse/Rabbit (DUO92101-1KT, Sigma-Aldrich). In all, 0.2 million WT B cells were spun onto slides (pre-coated with 1% of FBS) using a PrO-Cyt Centrifuge. Then, WT B cells were fixed with 4% formaldehyde in PBS for 20 min at room temperature and further permeabilized with 0.4% Triton X-100 for 30 min. After blocking with the blocking buffer in the kit (DUO92004-30RXN, Sigma-Aldrich), slides were treated with two different primary antibodies at 4 °C overnight. The primary antibodies, mouse monoclonal anti-WASP antibody (D-1) (Cat# SC-5300, Santa Cruz Biotechnology) and rabbit polyclonal isotype (Cat# ab37415, Abcam), were used as a negative control. The next day, Duolink® In Situ PLA® Probe Anti-Mouse MINUS and Duolink® In Situ PLA® Probe Anti-Rabbit PLUS were mixed together and applied to the samples. Each slide was incubated at 37 °C for 60 min. After washing, the ligation and amplification were performed following the Duolink® In Situ Detection Reagents Red kit instructions. If needed, an Alexa fluor 488 secondary antibody was applied to the sample to visualize the green immunofluorescence signal of target proteins. Finally, using Duolink® In Situ Mounting Medium with DAPI (DUO82040-5ML, Sigma-Aldrich) to stain nuclei with DAPI and protect PLA signals. PLA signals were imaged using a Zeiss LSM 880 with Airyscan function. To quantify the PLA signal number, we randomly picked 50 cells from five different images using a ×63 oil objective. PLA signals were checked carefully in the Z-stack mode in the ZEN software. For colocalization analysis, the color threshold function in the Fiji software was used. We define a yellow color (20–50) as colocalization, which is generated from the PLA signal in the red channel and SRSF2 or POI II signal in the green channel.

### Immunoprecipitation and immunoblot analysis

The *WAS*^null^ B cells and WT B cells described above or *WAS*^null^ B cells expressing FLAG-tagged wild-type WASP were lysed with a buffer comprising 50 mM Tris-HCl (pH 7.5), 150 mM NaCl, 0.5% Triton X-100, aprotinin (10 μg/ml), leupeptin (10 μg/ml), 1 mM PMSF, 400 μM Na3VO4, 400 μM EDTA, 10 mM NaF, and 10 mM sodium pyrophosphate. The lysates were centrifuged at 20,000×*g* for 10 min at 4 °C to remove debris, and the resulting supernatants were then incubated for 30 min at 4 °C with protein A—Sepharose 4 Fast Flow (Cat# P9424, Sigma) conjugated with antibodies as indicated. The resulting immunoprecipitates were washed three times with ice-cold lysis buffer, fractionated by SDS-PAGE, and subjected to immunoblot analysis with primary antibodies and HRP-conjugated secondary antibodies to either mouse (Cat# 7076, Cell Signaling Technology), or rabbit (7074, Cell Signaling Technology) IgG. Immune complexes were detected with an ECL system (GE Healthcare Cat# RPN2232).

### ChIP-seq and ChIP-qPCR

ChIP-sequencing: Three batches of 4 × 10^7^ cells were cross-linked, sonicated, and immunoprecipitated as directed by the EZ-Magna ChIP™ HiSens Kit (17-10461, Merk Millipore). Briefly, cells were cross-linked with formaldehyde (final concentration 1%) and sonicated using a Diagenode Bioruptor Pico programmed to 75 cycles 30” ON/30” OFF, and vortexed 10 s each 25 cycles; Sonicated DNA sizes were verified by 1.2% agarose gel electrophoresis. ChIP-grade protein A/G magnetic beads (20 µl/Rx) were washed and incubated for 2 h with 10 µg of WASP D-1 antibody (Cat# SC-5300, Santa Cruz Biotechnology) at 4° C on a rotating platform. The chromatin (4 × 10^7^ cells/Rx) was incubated with the antibody-coupled beads overnight at 4 °C on a rotating platform. On a magnetic rack, the beads were separated together with the linked DNA. To reverse cross-linking the immunoprecipitated samples and input controls (10 µL saved from the sonicated sample) were treated with RNase A and incubated on a thermomixer at 37 °C for 30 min; then, Proteinase K was added and incubated at 65° C for 2 h followed by 95 °C for 15 min. DNA was extracted with the ChIP DNA Clean and Concentrator Kit (D5205, Zymo Research). Three rounds of ChIP samples were pooled at 15 µL final volume (120 × 10^6^ cells per cell line). Library preparation was performed using the MicroPlex Library Preparation Kit v2 (C05010012, diagenode). Library purification and size selection were performed using AMPure® XP Beads Beckman Coulter. The purified Library concentration was measured by Bioanalyzer analysis, pooled at 2 nM, and sequenced at paired-end 150 bp on an Illumina HiSeq 4000 sequencer. ChIP-Seq reads were mapped to hg38 by DNASTAR SeqMan NGen v2.5.1 with default parameters.

ChIP-qPCR: Two batches of cells (2.5 × 10^7^ cells for each cell line) were cross-linked and sonicated as described above. The immunoprecipitations (1 × 10^7^ cell equivalent/Rxn) were carried out using the EZ-Magna Kit or in according to Fujita and Fujii (2015) with the 5 µg α-H3K27ac (39133, Active Motif) antibody and 5 µg isotype control IgG (ab37415, Abcam). In all immunoprecipitations, ChIP-grade protein A/G magnetic beads (20 µl/Rx) were washed and incubated for 2 h with 5 µg of antibody at 4 °C on a rotating platform. The chromatin was incubated with the antibody-coupled beads overnight at 4° C on a rotating platform. On a magnetic rack, the beads were separated together with the cross-linked DNA. To reverse cross-linking the immunoprecipitated chromatin and input control were incubated on a thermomixer at 65 °C for 2 h followed by 95 °C for 15 min in elution buffer and the addition of Proteinase K. DNA was extracted with the ChIP DNA Clean and Concentrator Kit (D5205, Zymo Research) and resuspended in 40 µl of Elution buffer each. For qPCR, 0.25 µl of DNA was added per reaction using the SsoAdvanced™ Universal SYBR® Green Supermix (BioRad) on a CFX384 real-time PCR detection system (BioRad). The same protocol was applied for WASP ChIP-seq validation using 10 µg WASP D-1 antibody (Cat# SC-5300, Santa Cruz Biotechnology) and 10 µg isotype control IgG (ab91353, Abcam). All primers used are shown in Supplementary Data 5.

### Flow cytometry

Cells were harvested and passed through a 70-µm strainer. The single cells were stained with antibodies against indicated cell surface markers and DAPI, and then analyzed on a BD LSRFortessa cytometer or FACS Aria II cytometer. Cell sorting was performed by the flow cytometry core facilities of the Salk Institute and UCSD on a FACS Aria II cytometer or a BD Influx cytometer. Figures exemplifying the gating strategy are provided in Supplementary Data 7.

### Small molecules

The following small molecules were used: sodium butyrate (Cat# 04-0005, Stemgent, Lexington, MA), working concentration: 0.25 mM. All drugs were dissolved in DMSO and used at concentrations as indicated.

### Proteomic analyses

MudPIT: We suspect that the sensitivity and specificity of MS analysis might be confounded by the close association between WASP and the ubiquitous cytoskeletal components. To improve confidence in specificity, we performed WAS co-IP in two independent B-lymphocyte lines, each with two well-characterized monoclonal antibodies. We then filtered out peptides identified in the IgG co-IP or in the *WAS*^null^ B-cell line by either of the two antibodies. To improve the detection of nuclear factors, we repeated the MudPIT analysis with highly purified nuclei and identified high-confidence WASP-interacting polypeptides. Proteins associated with endogenous WASP were co-immunoprecipitated with two different monoclonal antibodies (sc-13139, Santa Cruz Biotechnology; 557773, BD Biosciences) against WASP from wild-type B lymphoblastoid cell lines using the Active Motif Universal Magnetic Co-IP Kit. Co-IP using mouse IgG in wild-type cells and co-IP using WASP antibodies in WASP mutant cells were used to discriminate against nonspecific proteins. To specifically immunoprecipitate WASP-interacting proteins in the nucleus, nuclei were isolated using the Nuclei EZ Prep kit (Cat# NUC101, Sigma-Aldrich, St. Louis, MO) and processed as other co-IP experiments. The co-IP samples were subjected to MudPIT analysis as in refs. ^80,86,87^. Briefly, the immunoprecipitates were then eluted, reduced, and alkylated with iodoacetamide (10 mM final concentration), and trypsinized. Upon completion of the digestion, the proteins were desalted with a fused silica capillary desalting column and washed overnight. Following the desalting process, a 100-μm internal diameter capillary consisting of a 10-μm laser pulled tip packed with 10 cm 3-μm Aqua C18 material (Phenomenex) was attached to the filter union (desalting column–filter union–analytical column) and the entire split-column was placed in line with an Agilent 1100 quaternary HPLC (Palo Alto, CA) and analyzed using a modified 6-step separation^88^. Peptides eluted from the microcapillary column were electrosprayed directly into an LTQ 2-dimensional ion trap mass spectrometer (ThermoFinnigan) with the application of a distal 2.4 kV spray voltage. A cycle of one full-scan mass spectrum (400–1400 *m/z*) followed by 8 data-dependent MS/MS spectra at a 35% normalized collision energy was repeated continuously throughout each step of the multidimensional separation. Application of mass spectrometer scan functions and HPLC solvent gradients were controlled by the Xcalibur data system. As each step was executed, its spectra were recorded, and poor-quality spectra were removed. MS/MS spectra remaining after filtering were searched with the SEQUEST algorithm against the NCBI RefSeq Human protein database concatenated to a decoy database in which the sequence for each entry in the original database was reversed. SEQUEST results were assembled and filtered using the DTASelect (version 2.0) program. DTASelect 2.0 uses a linear discriminant analysis to dynamically set XCorr and DeltaCN thresholds for the entire dataset to achieve a user-specified false-positive rate. The false-positive rates are estimated by the program from the number and quality of spectral matches to the decoy database.

### Data-independent acquisition mass spectrometry (DIA-MS)

WT iMPs and WASP-KO-iMPs were harvested after 1-month differentiation with M-CSF and GM-CSF cytokines. About 2 × 10^6^ macrophages were lysed in 500 µl RIPA buffer with protease inhibitor cocktail and were sheared by sonication on ice (Qsonica XL-2000 ultrasonic liquid processor, US; 10 s per pulse, 3 pulses, 2-min interval time). The protein concentration of the supernatant was determined by Pierce^TM^ BCA protein assay kit (Thermo Fisher), after 14,000×*g* centrifugation for 20 min at 4 °C. A filter-aided sample preparation protocol^89,90^ with modifications was used for the sample processing. Briefly, the sample supernatant containing 10 µg total protein was mixed with 200 µl of 8 M urea in 0.1 M Tris/HCl (PH 8.5) in a Microcon-10kDa centrifugal filter unit (Millipore) and centrifuged at 14,000×*g* for 40 min. The samples were digested by trypsin (enzyme to protein ratio 1:50) at 37°C overnight. The filtrates were desalted using Sep-Pak column C18 cartridges (Waters). Approximately 1.5 µg of peptide mixture per sample was injected in single technical replicates and an Orbitrap Fusion Lumos mass spectrometer (Thermo Scientific) coupled with an UltiMate^TM^ 3000 UHPLC was used for DIA-MS analysis. A Spectronaut software against the Pan-Human library^91^ was applied for protein/peptide identification and quantification.

### Enzyme-linked immunosorbent assay (ELISA)

KO-iMPs were transduced with 30 pmol siRNA targeting SRSF2 per million cells by RNAiMAX (Thermo Fisher) and the same amount of non-targeting siRNA was transduced to both WT and KO-iMPs as control. After 24 h transfection, the cells were harvested and seeded 100k/well in 24-well with 1 µg/ml LPS in the medium. The supernatant was collected after 8 h LPS treatment and centrifuged at 500×*g* for 5 min at 4 °C to remove any cell debris. The TNF concentration in the supernatant was measured by an ELISA kit (PeproTech, Cat # 900-TM25) followed a standard protocol provided by the kit.

### Microscale thermophoresis (MST)

His6-tagged SRSF2 were labeled with RED –tris-NTA dye in PBS-T buffer (Nano-Temper-technology) with a ratio 1:2 dye to protein. The reaction was then incubated for 30 min at room temperature. The interaction of SRSF2 with WASP was measured using MST instrument monolith NT.115 in premium coated capillaries supplied by Nano temper. The recombinant WASP purchased from Abnova (Cat # H00007454-P01) is a full-length protein (1-502 a.a.) with a GST tag at the N-terminus that was expressed in an in vitro wheat germ expression system and purified by the Glutathione Sepharose 4 Fast Flow method. A series of 10 µl reaction of 16 different concentrations from 90 pM to 3 µM of WASP were prepared. Each reaction was mixed with 10 µl of SRSF2 labeled with RED-Tris-NTA to obtain a final concentration of 1 nM. All reactions were done in buffer (50 mM HEPES pH 7.5, 10 mM MgCl2, 100 mM KCl, 0.1 mg/ml BSA, 5% glycerol and 1 mM DTT). The fluorescent molecules were excited with a red laser (640 nm) to monitor the spatial distribution of the molecules in each capillary. The thermophoresis signals were measured in each capillary by a temperature gradient induced by an infrared (IR) laser with an emission wavelength of 1480 nm focused on a local defined sample volume. Upon heating, the protein molecules diffused away from the peak of the temperature and bound and unbound proteins respond differently. The change in the thermophoresis of florescent molecules due to binding was plotted and used to calculate the bound protein fraction and the dissociation constant was obtained by fitting the results to a binding isotherm.

### Expression and purification of recombinant human SRSF2

Human full-length SRSF2 (accession no. Q01130) N-terminally 8X histidine- and SUMO-tagged and C-terminus Strep-tagged *E. coli* optimized sequence was cloned into pET-24a by GenScript. SRSF2 plasmid was transformed into *E. coli* strain BL21 (DE3) competent cells (Novagen) and colonies were selected on agar plates containing 50 μg/ml kanamycin. SRSF2 was over-produced by growing the transformed cells in 10 L of 2YT media (Teknova) supplemented with kanamycin. Cells were grown at 37 °C to OD_600_ of 0.8 and then induced with 0.2 mM isopropyl β-d-thiogalactopyranoside (IPTG) and incubated further for 19 h at 16 °C. Cells were collected by centrifugation at 5500×*g* for 10 min and resuspended in lysis buffer [50 mM Tris (pH 8), 700 mM NaCl, 40 mM imidazole, 5 mM β-mercaptoethanol, 0.2% NP-40, 1 mM PMSF, 5% glycerol and EDTA free protease inhibitor cocktail tablet/50 ml (Roche, UK)]. All further steps were performed at 4 °C. Cells were lysed enzymatically by adding 2 mg/ml lysozyme and mechanically by sonication. Cell debris was removed by centrifugation (22,040×*g*, 30 min) and the clear supernatant was directly loaded onto HisTrap HP 5 ml affinity column ((GE Healthcare) pre-equilibrated with buffer A [50 mM Tris (pH 8), 700 mM NaCl, 40 mM imidazole, 5 mM β-mercaptoethanol and 5% glycerol]. The column was then washed with ten column volumes (CVs) of buffer A followed by elution gradient with ten CVs using buffer B [50 mM Tris (pH 8), 500 mM NaCl, 700 mM imidazole, 5 mM β-mercaptoethanol and 5% glycerol]. The peak fractions were pooled and dialyzed overnight in a dialysis buffer [50 mM Tris (pH 8), 300 mM NaCl, 5 mM β-mercaptoethanol, and 10% glycerol]. The dialyzed fractions were concentrated, flash frozen and stored at −80 °C.

### Sample preparation for RNA immunoprecipitation sequencing (RIP-seq)

WT B cells and *WAS*^null^ B (negative control) cells were harvested and 1 × 10^7^ cells per sample were used for whole-cell RIP following a published protocol^92^ or nuclear fraction RIP following another published protocol^93^. For whole-cell RIP, the cells were fixed with 0.1% formaldehyde for 10 min at room temperature followed by adding 125 mM glycine for 5 min at room temperature. The cells were washed twice with cold PBS and resuspended in 1 ml RIPA lysis buffer with freshly added 0.5 mM DTT, 1× protease inhibitor cocktail (PIC), and 100 U/ml RNaseOUT. After 10 min of incubation at 4 °C, sonication was performed by 30 s on and 45 s off for total 10 min using Bioruptor®Pico (Diagenode). The samples were centrifuged at 10,000×*g* for 10 min at 4 °C and mixed the supernatant with equal volume of RIP buffer (150 mM KCl, 25 mM Tris-HCl (PH 7.5), 5 mM EDTA, 0.5% NP-40, 0.5 mM DTT, 1× PIC and 100 U RNaseOUT. The 1% sample was saved as input and the rest was incubated with 6 µg WASP antibody (B9) for 2 hours at 4 °C and further incubated with 50 µl Dynabeads for 1 h at 4 °C. After washing the beads twice using RIP buffer, the beads were resuspended in 56 µl RNase-free water and 33 µl 3 x reverse cross-linking buffer (3×PBS, 6% N-lauroyl sarcosine, 30 mM EDTA, 15 mM DTT), 10 µl proteinase K and 1 µl RNaseOUT followed by incubation at 42 °C and 55 °C for 1 h, respectively. For the nucleation fraction RIP, the nuclei were isolated using nuclear isolation buffer (1.28 M sucrose, 40 mM Tris-HCl (PH 7.5), 20 mM MgCl_2_, 4% Triton X-100, 1×PIC and 100 U/ml RNaseOUT) and resuspended in 1 ml RIP buffer with 1-h incubation at 4 ˚C. Sonication was performed by 30 s on and 45 s off for total 10 min using Bioruptor®Pico (Diagenode). After 13, 000 rpm centrifugation for 10 min, the supernatant was collected, of which 1% was saved as input, the rest incubated with 6 µg WASP antibody (B9) overnight at 4 °C and further incubated with 50 µl protein G-coated magnetic beads for 3 h at 4 °C. The beads were washed with RIP buffer, low salt buffer (20 mM Tris-HCl (PH 7.4), 150 mM NaCl, 2 mM EDTA, 1% Triton, 0.1% SDS), high-salt buffer (20 mM Tris-HCl (pH 7.4), 500 mM NaCl, 2 mM EDTA, 1% Triton, 0.1% SDS), low salt buffer, high-salt buffer, and RIP buffer. The beads from the final step of both whole-cell RIP and nuclear fraction RIP and input samples were resuspended in 1 ml TRIzol followed by RNA purification. The Qubit Fluorometer was used for RNA concentration measurement and Bioanalyzer was used to determine RNA integrity prior to library preparation.

### Antibodies

Antibodies used in this study are listed in Supplementary Data 6.

### High-throughput whole transcriptome sequencing

RNA was isolated by Qiagen RNeasy kit with in-column DNase digestion. The Agilent TapeStation or Bioanalyzer was used to determine RNA integrity (RIN) numbers prior to library preparation. Stranded mRNA-Seq libraries were prepared using the TruSeq Stranded mRNA Library Prep Kit (Cat# RS-122-2101, Illumina) or TruSeq RNA Access Library Prep Kit (Cat# RS-301-2001, Illumina) according to the manufacturer’s instructions. Briefly, RNA with polyA tail was isolated using poly-T oligos conjugated to magnetic beads. mRNA was then fragmented and reverse-transcribed into cDNA. dUTPs were incorporated, followed by second-strand cDNA synthesis. The dUTP-incorporated second strand was not amplified. cDNA was then end-repaired, index adapter-ligated and PCR-amplified. Purification of nucleic acids after each enzymatic step was achieved by using AMPure XP beads (Beckman Coulter).

### Next-gen sequencing

mRNA Libraries were quantified, pooled, and sequenced at single-end 50 base pair (bp) using the Illumina HiSeq 2500, paired-end 75 bp using the Illumina NextSeq 500 platform or paired-end 150 bp using the Illumina NovaSeq 6000 platform at the Salk Next Generation Sequencing Core Lab, the KAUST Bioscience Core Lab or the Novogene Co. Ltd. Raw sequencing data were demultiplexed and converted into FASTQ files using CASAVA (v1.8.2). Libraries were sequenced at an average depth of 50 million reads per library.

### Bioinformatics analysis

Sequencing reads were aligned to the hg19 human genome using STAR (v2.5.1) (https://academic.oup.com/bioinformatics/article/29/1/15/272537) with default parameters. Cuffdiff (v2.2.1) (https://www.nature.com/articles/nbt.2450) was used to analyze the differentially expressed (DE) genes and isoforms (-b -u—library-type fr-firstrand) on a pair-wise experiment design. A default cutoff of *q*-value < 0.05 was used to identify the DE genes. The spliced ratio was defined as the expression of one isoform over all isoforms of a specific gene under one condition. Isoforms with a difference in spliced ratio > 20% between two conditions and FPKM (Fragments Per Kilobase of transcript per Million mapped reads) > 5 in both conditions were identified as differentially spliced (DS) isoforms. Pathway analysis was performed using DAVID (v6.8) (1) where the list of DE genes/DS isoforms was used as input and the whole genome was used as background. As an alternative isoform expression analysis method, we used STAR v2.5.3a to build a genome index from ENSEMBL GRCh38 human primary assembly. We augmented the index with the ENSEMBL annotation file release 90 through the—sjdbGTFfile command line parameter. The paired-end reads were then aligned using STAR v2.5.3a to the previously built human genome. Afterward, we used the QC command of QoRT (https://www.ncbi.nlm.nih.gov/pubmed/26187896) to generate counts for novel and annotated splice junctions based on the alignment files using the arguments (—stranded –runFunctions writeKnownSplices writeNovelSplices writeSpliceExon). Next, we used the mergeNovelSplices command to aggregate the splice junctions and filter out low coverage (<10 normalized read coverage) novel splice junctions resulting in flat exon and splice junction count files for each sample. We used the previously generated flat count files as input for JunctionSeq (https://www.ncbi.nlm.nih.gov/pubmed/27257077) to determine exon and splice junction differential usage. We followed the user manual to obtain the differentially used junctions and exons. Using the previously generated alignment files, we obtained gene-level counts through htseq-count v0.6.0, part of the HTSeq framework (https://www.ncbi.nlm.nih.gov/pubmed/25260700), based on ENSEMBL annotation file release 90 (https://www.ncbi.nlm.nih.gov/pubmed/27899575). We then used DESeq2 (https://www.ncbi.nlm.nih.gov/pubmed/25516281) to obtain the differentially expressed genes following the standard workflow. The splicing pattern was visualized using IGV. Gene expression cluster analysis was conducted using Cluster 3.0 (https://www.encodeproject.org/software/cluster/) and Java TreeView (jtreeview.sourceforge.net/). Dendrogram on the heatmap represented the hierarchical clustering of the gene expression based on their correlation using the average linkage method in Cluster 3.0. GO term enrichment analysis was performed using ShinyGO v0.61 (http://bioinformatics.sdstate.edu/go/). Ingenuity-pathway analysis of the disease and function categories affected using RNA-seq data was performed according to the manufacturer's instructions (https://www.qiagenbioinformatics.com/products/ingenuity-pathway-analysis/).

Skipped exon events were detected using rMATS (v4.0.1; 10.1073/pnas.1419161111) by evaluating reads that were mapped to the splicing junctions and the “on target” exons. Each condition was tested using two replicates of paired-end 75 bp reads. To get the reads with a fixed length, reads were pre-processed by cutadapt (v1.14; 10.14806/ej.17.1.200) to trim the first nucleotide (nt) off whenever necessary and aligned by STAR (v2.5.1) with default parameters and the soft-clipping option off. The resulted skipped exon events with >10% difference in splicing ratio and FDR < 0.05 were identified as significantly alternatively spliced (AS) exons.

Motif enrichment tests on the exons were performed using Homer (v4.8; PMID: 20513432). The RBPmap database^94^ containing motif information of 94 RNA binding proteins was used to scan the aberrant skipped exons of WASP-deficient cells, and their upstream or downstream exons, respectively. The motif lengths from 4 nt to 15 nt were tested for enrichment with respect to the background. Significantly enriched motifs were defined as motifs with hypergeometric *P* < 1e-2, greater than 5% enrichment in target sequences, and fold-enrichment > 1.5.

R (http://www.R-project.org/) was used to generate graphs, unless mentioned specifically. Venn diagrams were plotted using the R package VennDiagram (10.1186/1471-2105-12-35). ChIPpeakAnno package (10.1186/1471-2105-11-237) was used for the integrated analysis of the WASP ChIP-seq and AASEs.

Raw reads of RNA-seq data of B-cell lines were processed with FASTQC (https://www.bioinformatics.babraham.ac.uk/projects/fastqc/) and BBDuk (https://jgi.doe.gov/data-and-tools/bbtools/) in order to check the quality and remove low-quality bases and adapters. A minimum quality of 25 and a minimum read length of 35 nt were required. High-quality reads were then mapped against the Homo sapiens reference genome (NCBI GRCh38.p12) with STAR. FeatureCounts (10.1093/bioinformatics/btt656) were used to perform read summarization at the gene level, only reads with quality higher than 30 were used. In addition, strand-specific and paired-end modes were included. Statistical analyses and plots were generated with R. Lowly expressed genes were filtered out with the HTSFilter (10.1093/bioinformatics/btt350) package, then a differential expression analysis was performed using edgeR. Genes with an FDR less or equal than 0.05 were considered significantly differentially expressed. Differential Splicing Analysis was performed with the software RMATS using the trimmed reads as input for mapping. Only splicing events with an FDR < = 0.05 were considered differential.

### Statistical analysis

Results are shown as mean ±  SEM unless indicated otherwise. Comparisons were performed with two-sided Student’s *t* test, or Mann–Whitney test if data are not normally distributed, unless indicated otherwise.

### Reporting summary

Further information on research design is available in the Nature Research Reporting Summary linked to this article.

## Supplementary information

Supplementary Information

Description of Additional Supplementary Files

Supplementary Data 1

Supplementary Data 2

Supplementary Data 3

Supplementary Data 4

Supplementary Data 5

Supplementary Data 6

Supplementary Data 7

Supplementary Movie 1

Supplementary Movie 2

Supplementary Movie 3

Supplementary Movie 4

Reporting Summary

## Data Availability

The human GRCh38 genome (http://ftp.ensembl.org/pub/release-99/fasta/homo_sapiens/) was used as the reference genome. The RNA-seq data generated in this study including raw sequencing data and the processed gene expression tables have been deposited in the NCBI GEO database under the accession number GSE107963 and GSE165382, and in NCBI SRA database within the BioProject under the accession code PRJNA541823. Processed ChIP-sequencing data generated in this study have been deposited in the NCBI GEO under the accession number GSE126483. The mass spectrometry proteomics data generated in this study have been deposited to the ProteomeXchange Consortium via the PRIDE^95^ partner repository with the dataset identifier PXD033326. All data are available in the main text or the Supplementary Information. Source data are provided with this paper.

## Source data

Source Data
