# Author Correction: Wiskott-Aldrich syndrome protein forms nuclear condensates and regulates alternative splicing

**DOI:** 10.1038/s41467-022-32875-z

**Published:** 2022-09-08

**Authors:** Baolei Yuan, Xuan Zhou, Keiichiro Suzuki, Gerardo Ramos-Mandujano, Mengge Wang, Muhammad Tehseen, Lorena V. Cortés-Medina, James J. Moresco, Sarah Dunn, Reyna Hernandez-Benitez, Tomoaki Hishida, Na Young Kim, Manal M. Andijani, Chongwei Bi, Manching Ku, Yuta Takahashi, Jinna Xu, Jinsong Qiu, Ling Huang, Christopher Benner, Emi Aizawa, Jing Qu, Guang-Hui Liu, Zhongwei Li, Fei Yi, Yanal Ghosheh, Changwei Shao, Maxim Shokhirev, Patrizia Comoli, Francesco Frassoni, John R. Yates, Xiang-Dong Fu, Concepcion Rodriguez Esteban, Samir Hamdan, Juan Carlos Izpisua Belmonte, Mo Li

**Affiliations:** 1 Bioscience Program, Biological and Environmental Science and Engineering Division, King Abdullah University of Science and Technology (KAUST), Thuwal, 23955-6900 Kingdom of Saudi Arabia; 2 Gene Expression Laboratory, Salk Institute for Biological Studies, 10010 North Torrey Pines Road, La Jolla, CA 92037 USA; 3 Institute for Advanced Co-Creation Studies, Graduate School of Engineering Science, Osaka University, Osaka, Japan; 4 Department of Cell Biology, Scripps Research Institute, La Jolla, CA 92037 USA; 5 The Waitt Advanced Biophotonics Core Facility, Salk Institute for Biological Studies, 10010 North Torrey Pines Road, La Jolla, CA 92037 USA; 6 Altos Labs, Inc. 5510 Morehouse Drive, Suite 300, San Diego, CA 92121 USA; 7 Laboratory of Biological Chemistry, School of Pharmaceutical Sciences, Wakayama Medical University, 25-1 Shitibancho, Wakayama, Wakayama 640-8156 Japan; 8 Next-generation sequencing core, Salk Institute for Biological Studies, 10010 North Torrey Pines Road, La Jolla, CA 92037 USA; 9 Life Science Center, Tsukuba Advanced Research Alliance, University of Tsukuba, 1-1-1 Tennoudai, Tsukuba, Ibaraki 305-8577 Japan; 10 Department of Cellular & Molecular Medicine, University of California at San Diego, La Jolla, CA 92093 USA; 11 Integrative Genomics and Bioinformatics Core, Salk Institute for Biological Studies, 10010 North Torrey Pines Road, La Jolla, CA 92037 USA; 12 State Key Laboratory of Membrane Biology, Institute of Zoology, Chinese Academy of Sciences, Beijing, 100101 China; 13 University of Southern California, 1333 San Pablo Street, MMR 618, Los Angeles, CA 90033 USA; 14 Ambys Medicines, 131 Oyster Point Blvd. Suite 200, South San Francisco, CA 94080 USA; 15 Pediatric Hematology/Oncology and Cell Factory, Fondazione IRCCS Policlinico San Matteo, Pavia, Italy; 16 Department of Research Laboratories and Director of Center for Stem Cell and Cell Therapy, Instituto G. Gaslini Children Hospital Scientific Institute, 16147 Genova, Italy

**Keywords:** Biological techniques, Stem cells

Correction to: *Nature Communications* 10.1038/s41467-022-31220-8, published online 25 June 2022.

The original version of this Article contains an error in the order of authorship of Mo Li and Juan Carlos Izpisua Belmonte.

This has been corrected in both the HTML and PDF versions of the Article.

