# Supplementary material for: Wiskott-Aldrich syndrome protein forms nuclear condensates and regulates alternative splicing
Source: Nat Commun. 2022 Jun 25;13:3646. doi: 10.1038/s41467-022-31220-8 (PMC9233711; doi:10.1038/s41467-022-31220-8)
Supplement: Supplementary file 1 — Supplementary Information [file 41467_2022_31220_MOESM1_ESM.pdf]

SUPPLEMENTARY INFORMATION

SUPPLEMENTAL Fig. S1-S12

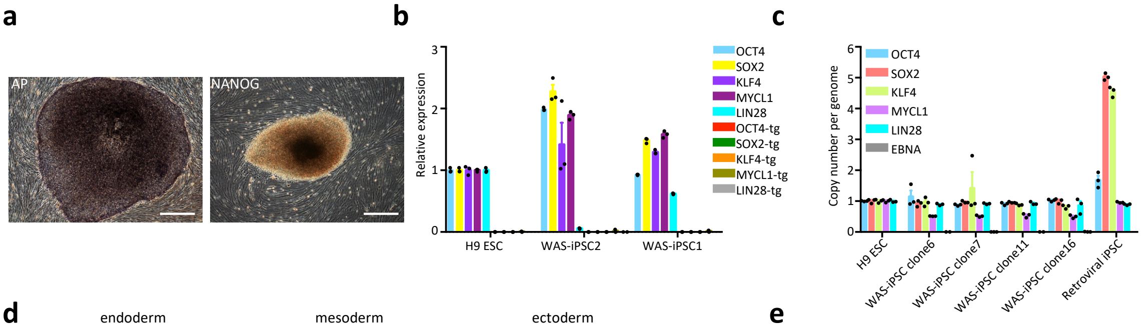

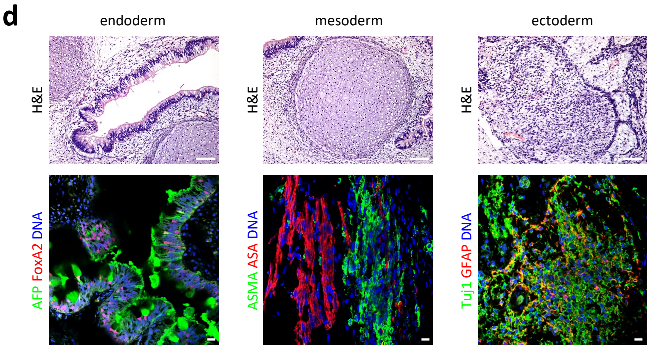

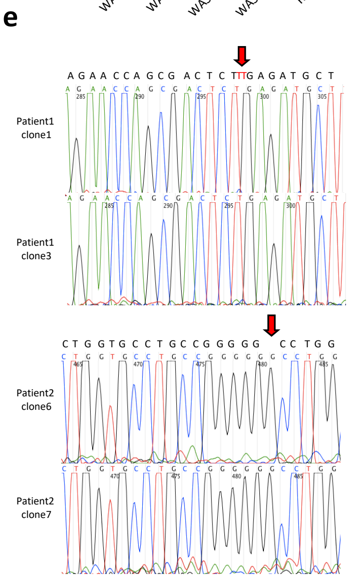

f

Strains	# of infected cells	# of G418[R] GANC[R] [a]	# of gene targeted clone [b]	gene targeting (%) [b/a x100]	# of gene corrected clone [c]	**gene correction (%) [c/a x100]**
Patient1 clone1	$2.5x10^6$	6	4	67	3	**50**
Patient1 clone4	$3.0x10^6$	23	12	52	8	**35**
Patient2 clone6	$2.0x10^6$	20	14	70	1	**5**

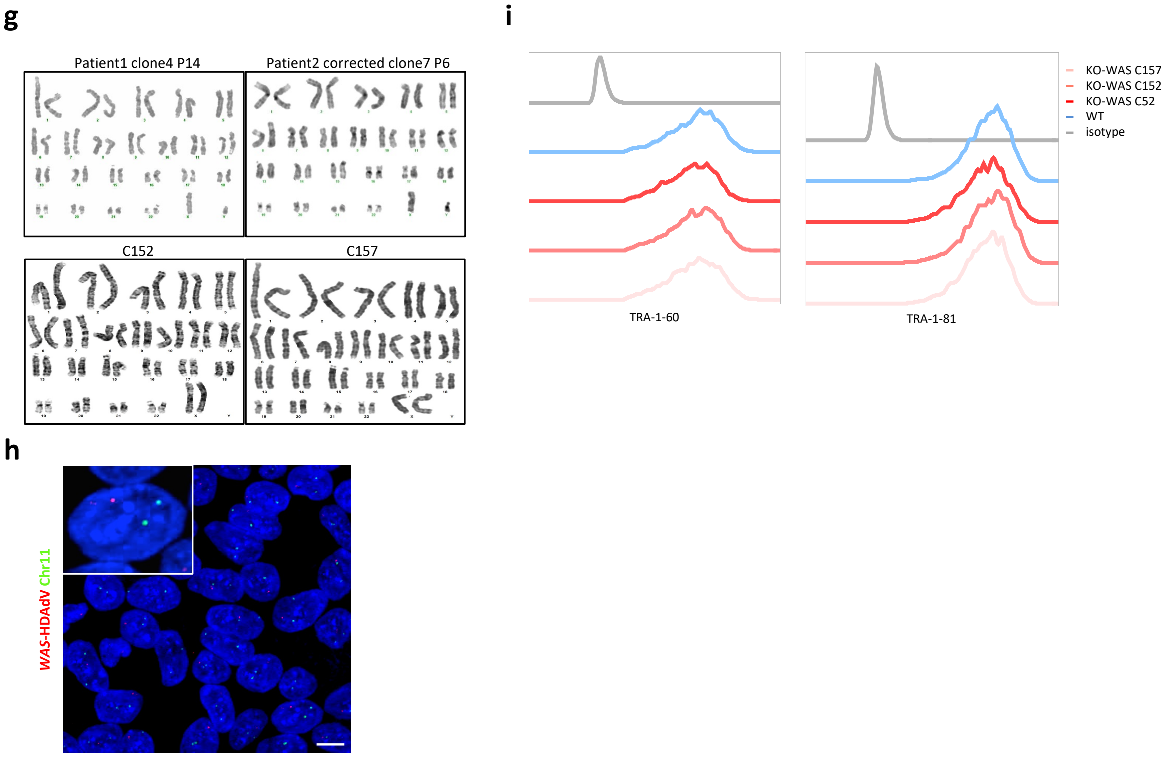

Fig. S1 Generation of WAS-specific iPSCs and isogenic gene-corrected iPSCs, related to Fig. 1.

(a) Representative Alkaline phosphatase (AP) and NANOG immunostaining of colonies of reprogrammed WAS patient fibroblasts. Scale bar = 0.5 mm. The experiment was repeated at least 3 times independently.

(b) Quantitative RT-PCR (qRT-PCR) analysis of the expression of endogenous pluripotency transcription factors (*Oct4*, *Sox2*, *Klf4*, *Mycl1* and *Lin28*) or transgenes (*Oct4-tg*, *Sox2-tg*, *Klf4-tg*, *Mycl1-tg* and *Lin28-tg*) in the two patient WAS-iPSCs. No exogeneous reprogramming factor expression was detected. The qPCR was performed in triplicate per sample, data are shown as mean ± SEM.

(c) Quantification of the copy number of episomal vectors that may integrate in the genome. No ectopic reprogramming factor or episomal vector sequence was detected. An iPSC line generated with integrative retroviruses was included as a control. Copy numbers of the reprogramming factor transgenes were normalized by the copy number of the same gene in the H9 ESC, except for copy numbers of *EBNA1* that doesn't exist in H9 ESC and were normalized by copy number of the *FBXO15* gene. Mean ± SEM, biological replicate for WAS iPSCs n = 4, for H9 ESCs n = 1, for retroviral iPSC n = 1.

(d) Representative hematoxylin and eosin (H&E) histological staining and immunostaining in teratomas derived from a randomly selected gene-corrected WAS-iPSC clone show in vivo differentiation towards ectodermal, mesodermal and endodermal tissues. The experiment was repeated at least 3 times independently. Scale bar = 50 μm.

(e) Verification of patient specific mutations by DNA sequencing in WAS-iPSC clones. Wild-type sequence is shown on top of the chromatographs.

(f) Gene-targeting and gene-correction efficiencies at the WAS locus in WAS-iPSCs achieved in multiple clones.

(g) Karyotyping analysis revealed normal karyotypes in randomly selected clones of WAS-iPSC lines, either before or after gene editing.

(h) Representative fluorescence in situ hybridization image of random integration of WAS-c-HDAdV. Interphase iPSC nuclei were hybridized with a TAMRA-labeled probe specific for WAS-c-HDAdV. Gene-corrected WAS-iPSCs showed one hybridization signal (red puncta) per nucleus. A chromosome 11 probe (green puncta) was used as control. Scale bar =10 μm. Blue: DAPI. The experiment was done once.

(i) FACS analysis of cell surface pluripotency markers TRA-1-60 and TRA-1-81 in WT and KO-iPSCs.

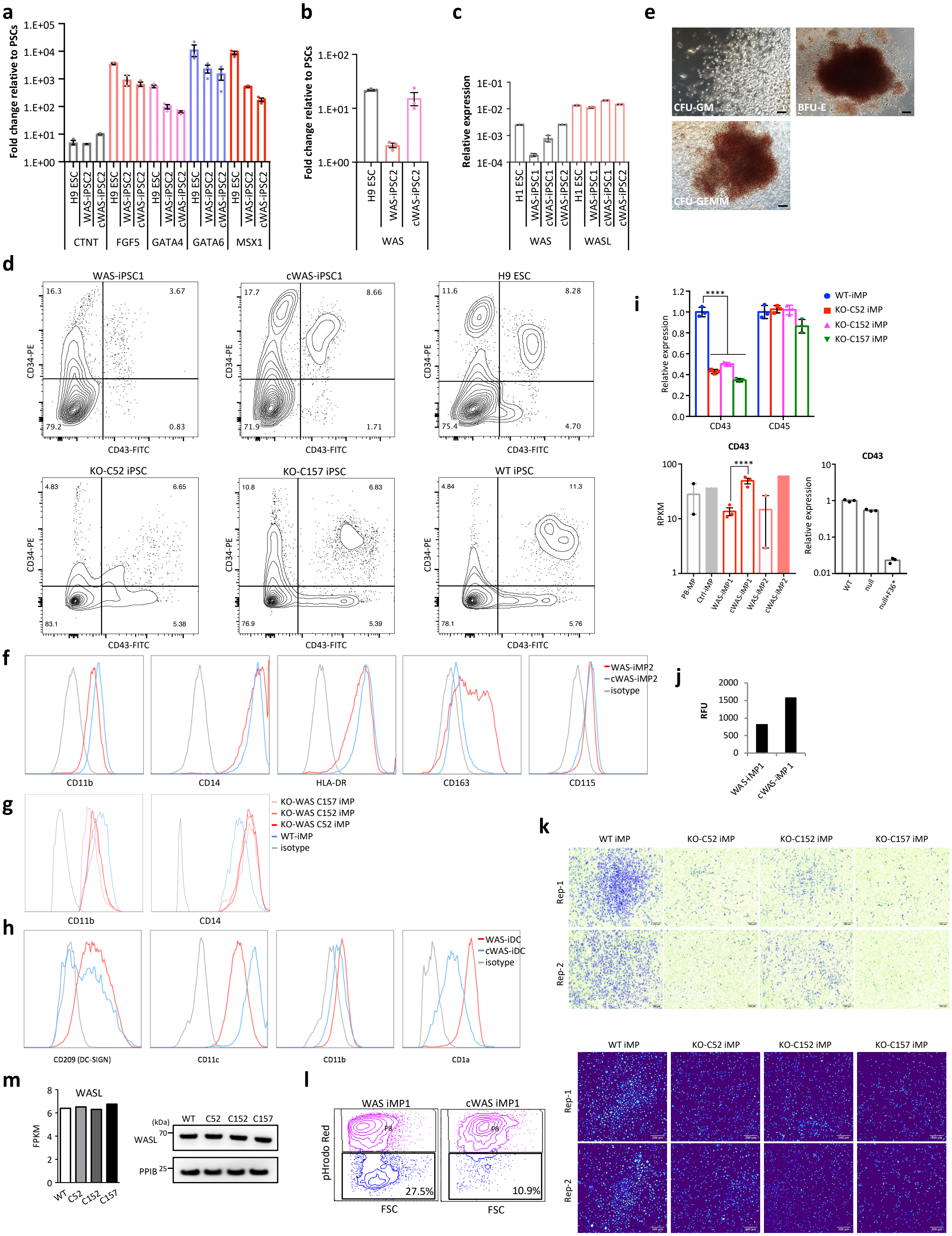
a
Fold change relative to PSCs
1.E+05
1.E+04
1.E+03
1.E+02
1.E+01
1.E+00
H9 ESC
WAS-iPSC2
cWAS-iPSC2
CTNT
FGF5
GATA4
GATA6
MSX1
b
Fold change relative to PSCs
1.E+02
1.E+01
1.E+00
H9 ESC
WAS-iPSC2
cWAS-iPSC2
WAS
c
Relative expression
1E-01
1E-02
1E-03
1E-04
H1 ESC
WAS-iPSC1
cWAS-iPSC1
WAS-iPSC2
cWAS-iPSC2
WAS
WASL
e
CFU-GM
BFU-E
CFU-GEMM
d
WAS-iPSC1
cWAS-iPSC1
H9 ESC
KO-C52 iPSC
KO-C157 iPSC
WT iPSC
CD34-PE
CD43-FITC
16.3
3.67
79.2
0.83
17.7
8.66
71.9
1.71
11.6
8.28
75.4
4.70
4.83
6.65
83.1
5.38
10.8
6.83
76.9
5.39
4.84
11.3
78.1
5.76
i
Relative expression
1.2
1.0
0.8
0.6
0.4
0.2
0.0
****
CD43
CD45
WT-iMP
KO-C52 iMP
KO-C152 iMP
KO-C157 iMP
CD43
RPKM
100
10
1
****
PB-MP
Ctrl-iMP
WAS-iMP1
cWAS-iMP1
WAS-iMP2
cWAS-iMP2
CD43
Relative expression
10
1
0.1
0.01
WT
null
null+F36*
f
CD11b
CD14
HLA-DR
CD163
CD115
WAS-iMP2
cWAS-iMP2
isotype
j
RFU
2000
1500
1000
500
0
WAS-iMP1
cWAS-iMP1
g
KO-WAS C157 iMP
KO-WAS C152 iMP
KO-WAS C52 iMP
WT-iMP
isotype
CD11b
CD14
k
WT iMP
KO-C52 iMP
KO-C152 iMP
KO-C157 iMP
Rep-1
Rep-2
h
WAS-iDC
cWAS-iDC
isotype
CD209 (DC-SIGN)
CD11c
CD11b
CD1a
m
WASL
FPKM
8
6
4
2
0
WT
C52
C152
C157
(kDa)
WT
C52
C152
C157
70
WASL
25
PPIB
l
WAS iMP1
cWAS iMP1
pHrodo Red
P8
27.5%
10.9%
FSC
WT iMP
KO-C52 iMP
KO-C152 iMP
KO-C157 iMP
Rep-1
Rep-2

Fig. S2 Recapitulation of WAS phenotypes using isogenic WAS iPSC models to Fig. 2.

(a-c) qRT-PCR analysis of the expression of: endodermal (*GATA4* and *GATA6*), mesodermal (*CTnT* and *MSX1*), and ectodermal (*FGF5*) genes in embryoid bodies (EBs) generated from H9 ESCs, WAS-iPSCs and cWAS-iPSCs (a); the *WAS* gene in EBs generated from H9 ESCs, WAS-iPSCs and cWAS-iPSCs (b); and the *WAS* and *WASL* genes in ESCs and iPSCs following hematopoietic differentiation (c). The qPCR was performed in triplicate per sample, data are shown as mean ± SD.

(d) Representative FACS analysis of HPC populations in differentiated iPSCs and ESCs. Cells are in the Tra-1-85+ gate. Numbers represent percentages.

(e) Representative images of hematopoietic colonies differentiated from WAS-iPSCs. Bar = 0.1 mm. The experiment was repeated at least 3 times independently.

(f-h) FACS analysis of iMPs and iDCs. Histograms show expression of typical cell surface markers of macrophages (f, g) and dendritic cells (h).

(i) Top: qRT-PCR analysis of the *CD43* gene in KO iMPs. Biological replicate n=3. ****$p < 0.0001$; bottom: expression values of *CD43* measured in RPKM (Reads Per Kilobase of transcript per Million mapped reads) in macrophages, biological replicate n=2 (PB-MP), 1 (Ctrl-iMP), 3 (WAS-iMP1 and cWAS-iMP1), 2 (WAS-iMP2), 1 (cWAS-iMP2). Hypergeometric testing P-values are corrected for multiple testing using FDR, ****$p = 5 \times 10^{-5}$; bottom right: qRT-PCR analysis of the expression of the CD43 gene in B cells. The qPCR was performed in triplicate per sample, data are shown as mean ± SD.

(j) Quantitative measurement of transwell migration of iMPs (n=1) as shown in Fig. 2C. RFU: relative fluorescence unit.

(k) Transwell migration assay as shown in Fig. 2c. The experiment was repeated 3 times independently. Scale bar = 200 μm.

(l) Left: FACS contour plots of phagocytic activity of WAS-iMP1 and cWAS-iMP1; right: Replicates of fluorescent images as shown in Fig. 2d. Scale bar = 200 μm.

(m) The FPKM value of the *WASL* gene in WT (n=2) and KO iMPs (n=3). Western blot of N-WASP (WASL) in WT and KO iMPs. PPIB: loading control.

All statistics in this figure were done using two-sided Student's t-test unless indicated otherwise. Data are shown as mean ± SEM.

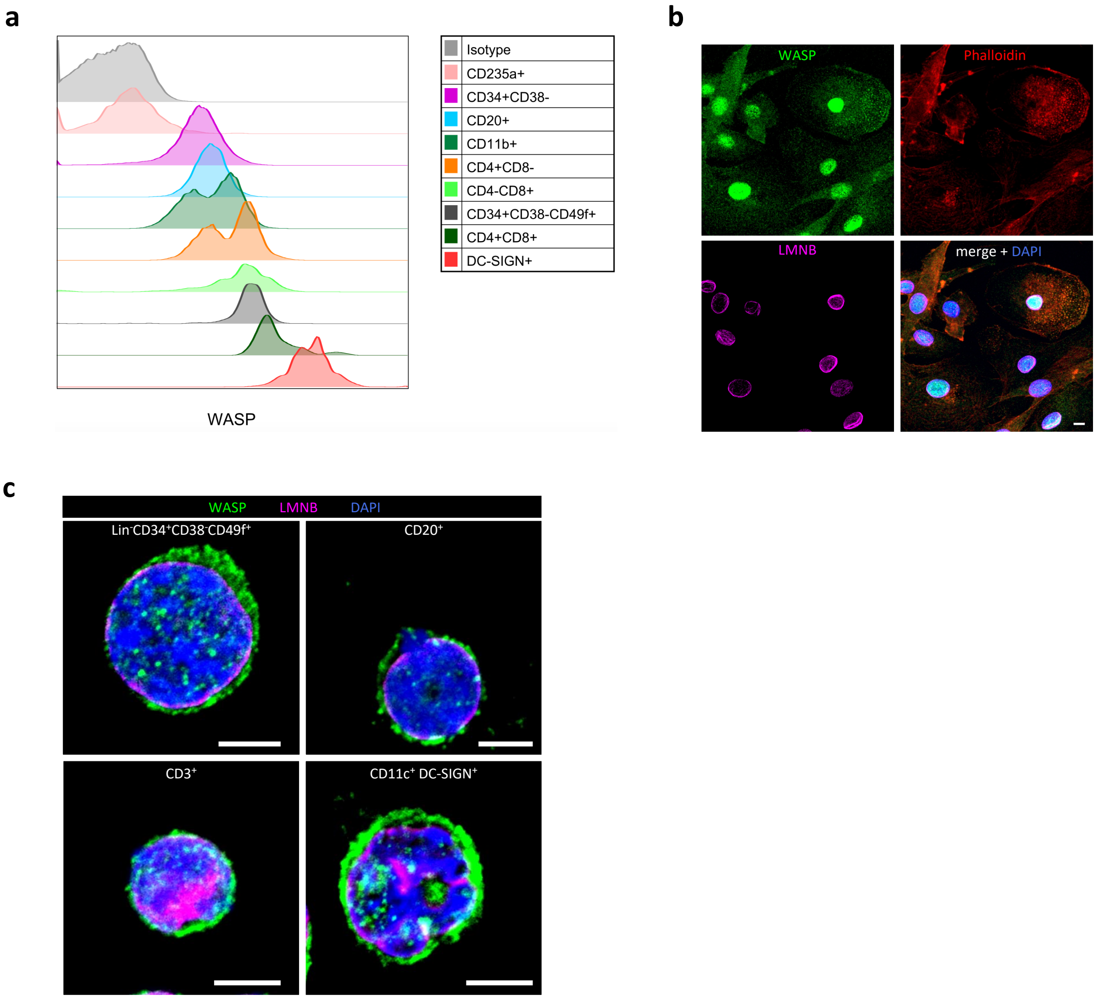

Fig. S3 Localization of wild type WASP in hematopoietic lineages.

(a) FACS analysis of WASP expression by intracellular staining in indicated hematopoietic lineages from human umbilical cord blood cells.

(b) Representative immunofluorescence images of nuclear and cytoplasmic localization of WASP in wild-type macrophages. Lamin B1 and phalloidin were used as nuclear and cytoplasmic landmarks, respectively. DNA was stained with DAPI. Bar = 10 µm. The experiment was repeated at least 3 times independently.

(c) Representative immunofluorescence image of the nuclear and cytoplasmic localization of WASP in wild-type hematopoietic stem/progenitor cells ($Lin^{-}CD34^{+}CD38^{-}CD49f^{+}$), B cells ($CD20^{+}$), T cells ($CD3^{+}$) and dendritic cells ($CD11c^{+}$ $DC\text{-}SIGN^{+}$). Note that blood cells have scant cytoplasm that is demarcated from the nucleus by LMNB staining. Green: WASP; Magenta: LMNB; Blue: DNA. Bar = 5 µm. The experiment was done once.

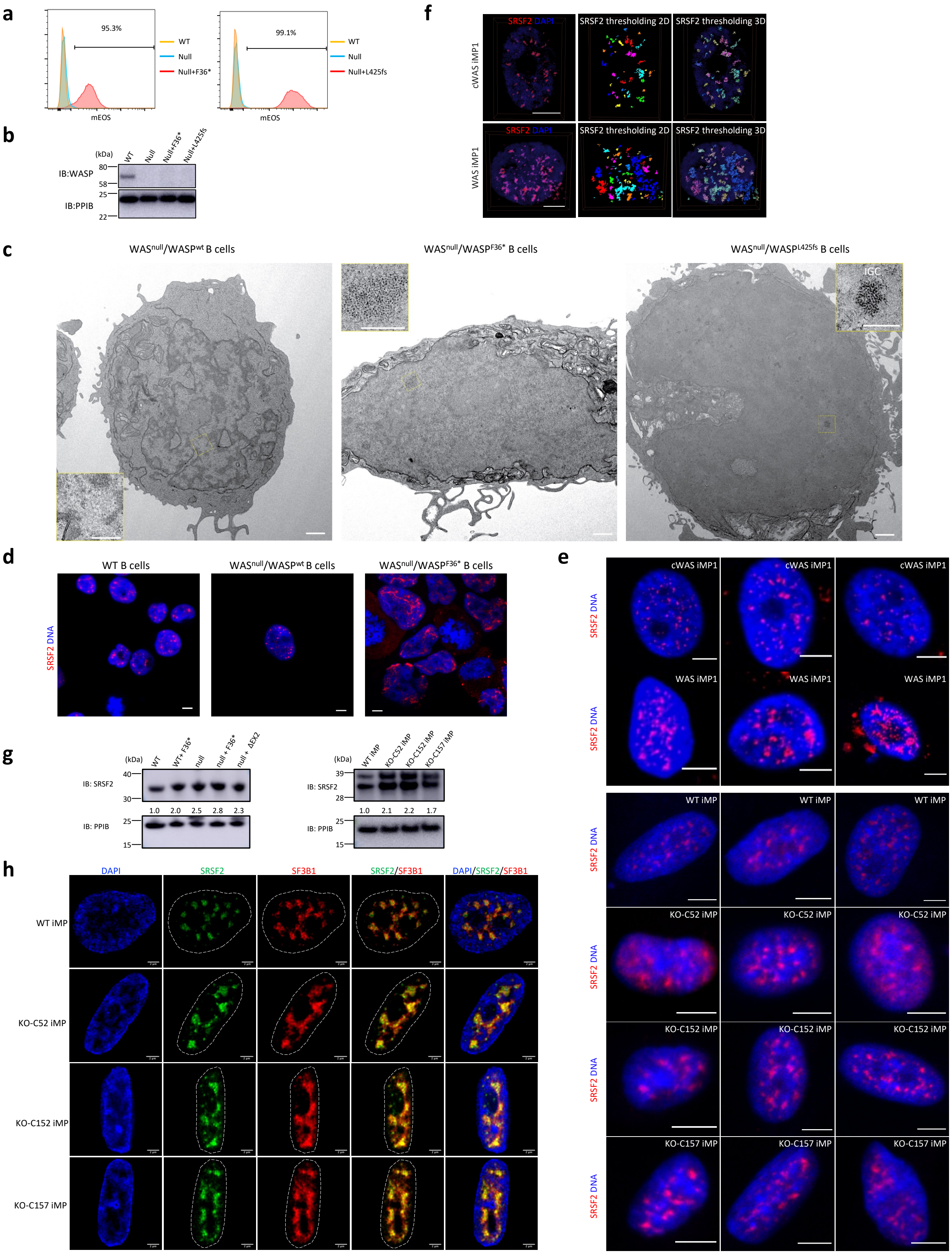
a
95.3%
WT
Null
Null+F36*
mEOS
99.1%
WT
Null
Null+L425fs
mEOS
b
(kDa)
WT
Null
Null+F36*
Null+L425fs
IB:WASP
IB:PPIB
c
WASnull/WASPwt B cells
WASnull/WASPF36* B cells
WASnull/WASPL425fs B cells
IGC
d
WT B cells
WASnull/WASPwt B cells
WASnull/WASPF36* B cells
SRSF2 DNA
e
cWAS iMP1
WAS iMP1
WT iMP
KO-C52 iMP
KO-C152 iMP
KO-C157 iMP
f
SRSF2 DAPI
SRSF2 thresholding 2D
SRSF2 thresholding 3D
cWAS iMP1
WAS iMP1
g
(kDa)
WT
WT+ F36*
null
null + F36*
null + ΔEX2
IB: SRSF2
IB: PPIB
1.0 2.0 2.5 2.8 2.3
WT iMP
KO-C52 iMP
KO-C152 iMP
KO-C157 iMP
1.0 2.1 2.2 1.7
h
DAPI
SRSF2
SF3B1
SRSF2/SF3B1
DAPI/SRSF2/SF3B1
WT iMP
KO-C52 iMP
KO-C152 iMP
KO-C157 iMP

Fig. S4 WASP deficiency results in striking changes in the morphology of nuclear speckles and overexpression of SRSF2 in B cell lines.

(a) Flow cytometry analysis of the expression of $WASP^{F36*}$-mEOS and $WASP^{L425fs}$-mEOS in $WASP^{null}$ B cells.

(b) Representative Western blot analysis of WASP in B cell lines with PPIB as a loading control. The experiment was repeated at least 3 times independently.

(c) Representative TEM images of WAS^{null} B cell lines overexpressing wild-type WASP, $WASP^{F36*}$ and $WASP^{L425fs}$. Bar = 1 μm in large panels; Bar = 0.5 μm in insets. IGC: interchromatin granule cluster. The experiment was done once.

(d) Representative confocal immunofluorescence image of the nuclear speckle marker SRSF2. Nuclei were stained with DAPI. Bar = 5 μm. The experiment was repeated at least twice independently.

(e) Representative single-plane confocal images of cWAS iMP1s, WAS iMP1s, WT iMPs and KO iMPs. Scale bar = 5 μm. The experiment was repeated at least 3 times independently.

(f) Examples of segmentation of nuclear speckles in cWAS iMP1 and WAS iMP1. Scar bar = 5 μm.

(g) Representative Western blot analysis of SRSF2 protein in B cell lines, WT iMPs and KO-iMPs. PPIB was used as a loading control. The numbers represent the normalized expression value of SRSF2 relative to WT B cells or WT iMP cells. The experiment was repeated at least twice independently.

(h) Representative single-plane confocal images of nuclear speckle markers, SRSF2 and SF3B1, in WT and KO-iMPs. Scale bar = 2 μm. The experiment was repeated twice independently.

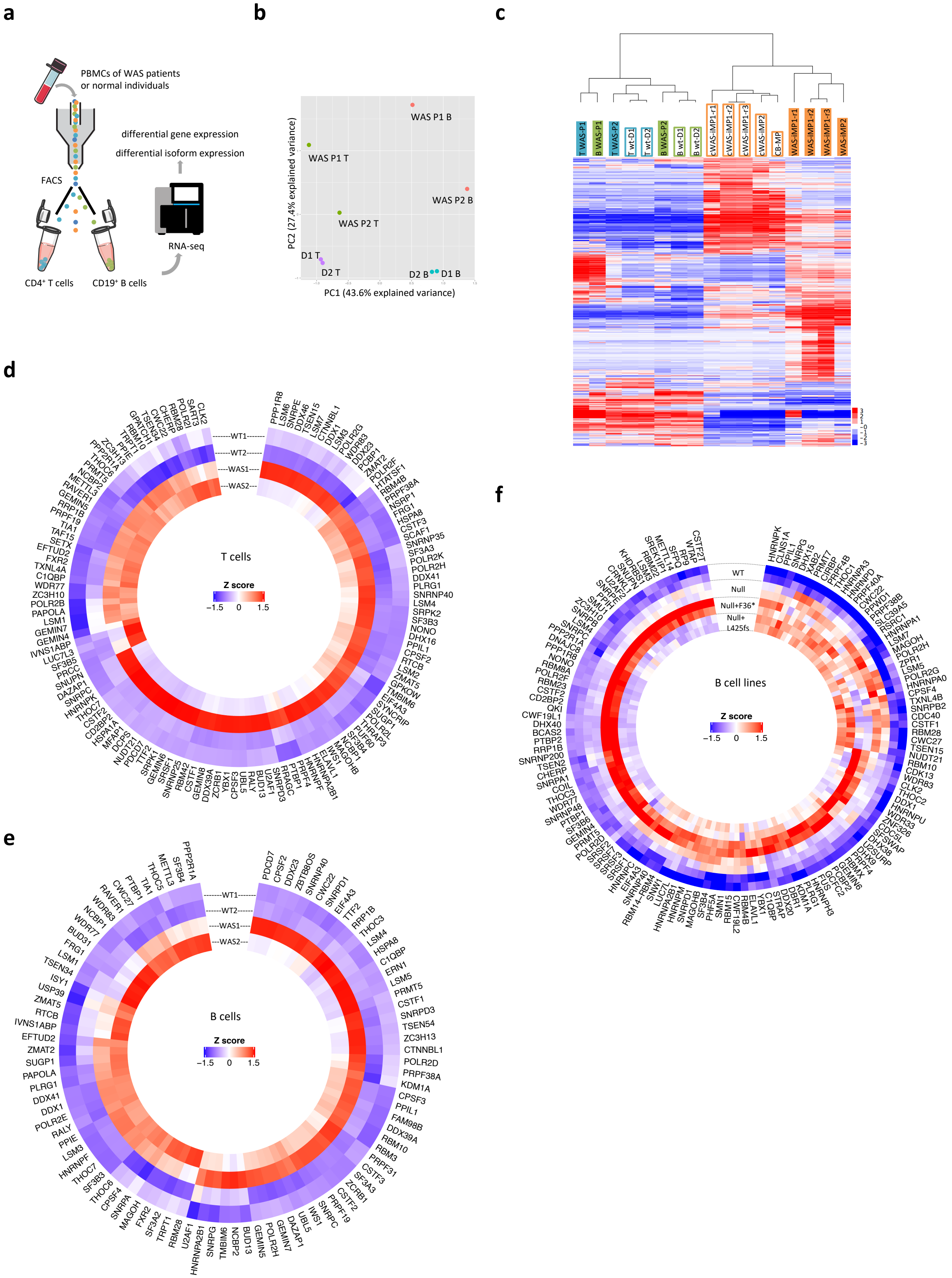

a
PBMCs of WAS patients
or normal individuals
differential gene expression
differential isoform expression
FACS
RNA-seq
CD4⁺ T cells
CD19⁺ B cells
b
PC2 (27.4% explained variance)
PC1 (43.6% explained variance)
WAS P1 B
WAS P1 T
WAS P2 B
WAS P2 T
D1 T
D2 T
D2 B
D1 B
c
T WAS-P1
B WAS-P1
T WAS-P2
T wt-D1
T wt-D2
B WAS-P2
B wt-D1
B wt-D2
cWAS-iMP1-r1
cWAS-iMP1-r2
cWAS-iMP1-r3
cWAS-iMP2
CB-MP
WAS-iMP1-r1
WAS-iMP1-r2
WAS-iMP1-r3
WAS-iMP2
d
WT1
WT2
WAS1
WAS2
T cells
Z score
−1.5
0
1.5
e
WT1
WT2
WAS1
WAS2
B cells
Z score
−1.5
0
1.5
f
WT
Null
Null+F36*
Null+
L425fs
B cell lines
Z score
−1.5
0
1.5

Fig. S5 WASP deficiency increases the expression of splicing factors in B cell lines and primary T and B cells.

(a) A schematic diagram of the design of experiments of patient PBMCs.

(b) PCA of RNA-seq data of patients (P1 and P2, clinical score of 4 and 2, respectively) and normal donors (D1 and D2).

(c) Hierarchical clustering of all differentially expressed genes among indicated samples. Note that patient T and B cells formed separate clusters outside of the T or B cell clusters, indicating that disease-associated gene expression signature is dominant over the cell-type specific transcriptional program.

(d) Heatmap of upregulated splicing factors in WAS-T cells, two replicates per cell line.

(e) Heatmap of upregulated splicing factors in WAS-B cells, two replicates per cell line.

(f) Heatmap of upregulated splicing factors in B cell lines, two replicates per cell line.

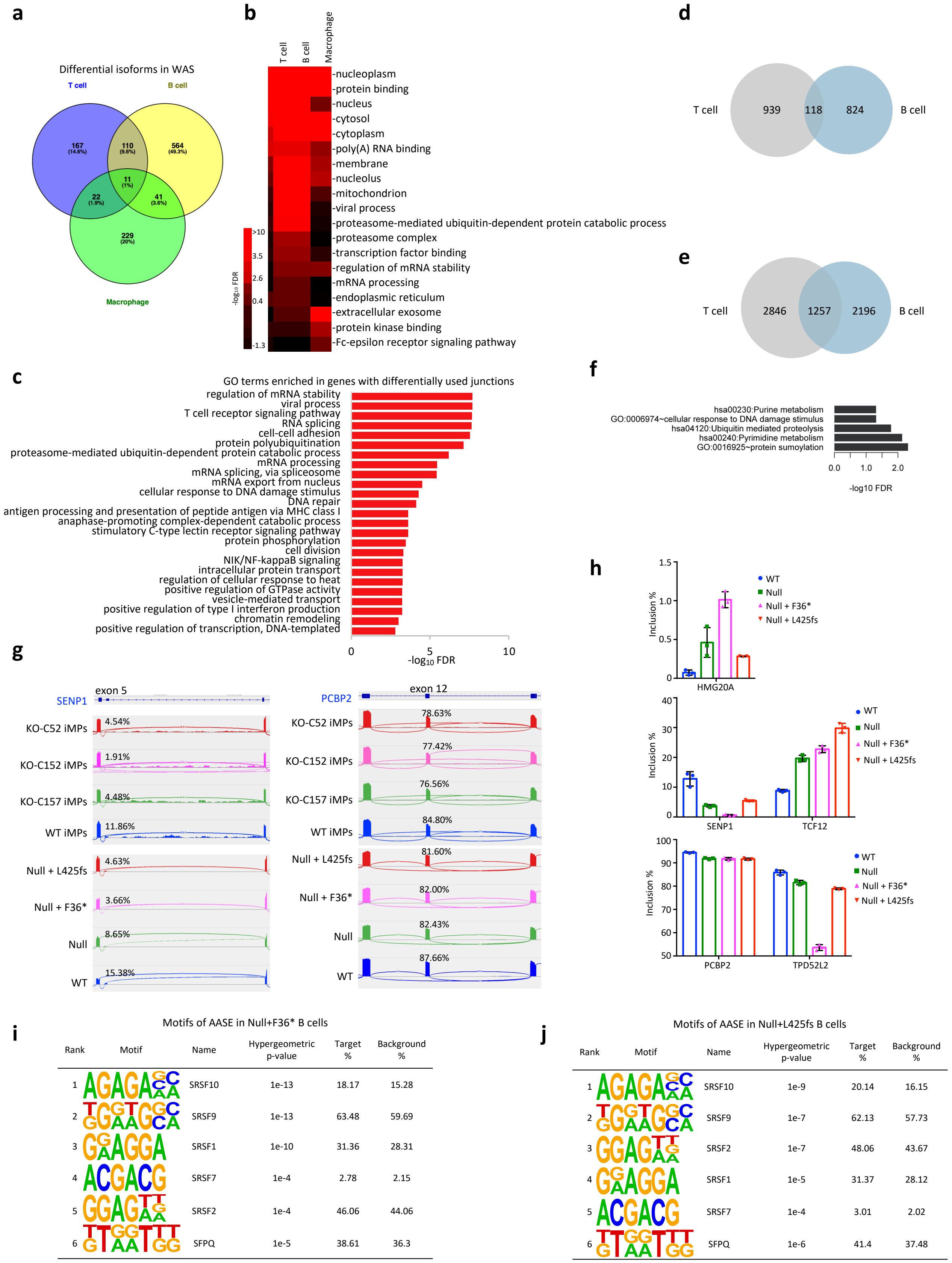
a
Differential isoforms in WAS
T cell
B cell
Macrophage
167 (14.8%)
110 (9.8%)
564 (49.3%)
11 (1%)
22 (1.9%)
41 (3.6%)
229 (20%)
b
T cell
B cell
Macrophage
-log10 FDR
>10
3.5
2.6
0.4
-1.3
-nucleoplasm
-protein binding
-nucleus
-cytosol
-cytoplasm
-poly(A) RNA binding
-membrane
-nucleolus
-mitochondrion
-viral process
-proteasome-mediated ubiquitin-dependent protein catabolic process
-proteasome complex
-transcription factor binding
-regulation of mRNA stability
-mRNA processing
-endoplasmic reticulum
-extracellular exosome
-protein kinase binding
-Fc-epsilon receptor signaling pathway
c
GO terms enriched in genes with differentially used junctions
regulation of mRNA stability
viral process
T cell receptor signaling pathway
RNA splicing
cell-cell adhesion
protein polyubiquitination
proteasome-mediated ubiquitin-dependent protein catabolic process
mRNA processing
mRNA splicing, via spliceosome
mRNA export from nucleus
cellular response to DNA damage stimulus
DNA repair
antigen processing and presentation of peptide antigen via MHC class I
anaphase-promoting complex-dependent catabolic process
stimulatory C-type lectin receptor signaling pathway
protein phosphorylation
cell division
NIK/NF-kappaB signaling
intracellular protein transport
regulation of cellular response to heat
positive regulation of GTPase activity
vesicle-mediated transport
positive regulation of type I interferon production
chromatin remodeling
positive regulation of transcription, DNA-templated
0
5
10
-log10 FDR
d
T cell
939
118
824
B cell
e
T cell
2846
1257
2196
B cell
f
hsa00230:Purine metabolism
GO:0006974~cellular response to DNA damage stimulus
hsa04120:Ubiquitin mediated proteolysis
hsa00240:Pyrimidine metabolism
GO:0016925~protein sumoylation
0.0
1.0
2.0
-log10 FDR
g
SENP1
exon 5
KO-C52 iMPs
4.54%
KO-C152 iMPs
1.91%
KO-C157 iMPs
4.48%
WT iMPs
11.86%
Null + L425fs
4.63%
Null + F36*
3.66%
Null
8.65%
WT
15.38%
PCBP2
exon 12
KO-C52 iMPs
78.63%
KO-C152 iMPs
77.42%
KO-C157 iMPs
76.56%
WT iMPs
84.80%
Null + L425fs
81.60%
Null + F36*
82.00%
Null
82.43%
WT
87.66%
h
Inclusion %
WT
Null
Null + F36*
Null + L425fs
HMG20A
SENP1
TCF12
PCBP2
TPD52L2
i
Motifs of AASE in Null+F36* B cells
Rank | Motif | Name | Hypergeometric p-value | Target % | Background %
1 | AGAGA | SRSF10 | 1e-13 | 18.17 | 15.28
2 | TGGTGGC | SRSF9 | 1e-13 | 63.48 | 59.69
3 | GAAGGA | SRSF1 | 1e-10 | 31.36 | 28.31
4 | ACGACG | SRSF7 | 1e-4 | 2.78 | 2.15
5 | GGAGTT | SRSF2 | 1e-4 | 46.06 | 44.06
6 | TTGGTTT | SFPQ | 1e-5 | 38.61 | 36.3
j
Motifs of AASE in Null+L425fs B cells
Rank | Motif | Name | Hypergeometric p-value | Target % | Background %
1 | AGAGA | SRSF10 | 1e-9 | 20.14 | 16.15
2 | TGGTGGC | SRSF9 | 1e-7 | 62.13 | 57.73
3 | GGAGTT | SRSF2 | 1e-7 | 48.06 | 43.67
4 | GAAGGA | SRSF1 | 1e-5 | 31.37 | 28.12
5 | ACGACG | SRSF7 | 1e-4 | 3.01 | 2.02
6 | TTGGTTT | SFPQ | 1e-6 | 41.4 | 37.48

Fig. S6 WASP mutations disrupt normal AS programs, related to Fig. 4.

(a) Venn diagram shows shared and distinct differentially expressed isoforms in the three cell types.

(b) Heatmap shows GO enrichment of differentially expressed isoforms in the three cell types.

(c) GO enrichment of genes with differentially used junctions detected by Junction-seq between T cells from WAS patients (n = 2) and normal donors (n = 2).

(d) Venn diagram showing the number of exons that are repressed in B and T cells from WAS patients compared to those from normal individuals.

(e) Venn diagram showing the number of exons that are promoted in B and T cells from WAS patients compared to those from normal individuals.

(f) GO Biological Process and KEGG pathway enrichment in genes containing commonly promoted exons in B and T cells from patients (n=2) with WAS.

(g) Isoform-level analysis of RNAseq data of macrophages and B cell lines. The graph below shows RNA-seq reads mapped to the cassette exon (middle) and its neighboring exons of the *SENP1* and *PCBP2* genes. The numbers above the IGV tracks represent the percentage of exon-retained isoform in total isoforms.

(h) Selected AASEs validated by probe-based qPCR. The qPCR was performed in triplicate per sample, data are shown as mean ± SD, mutant cell lines n = 3.

(i) Splicing factor motif analysis from downstream exon of altered SE in $WASP^{null+F36*}$ B cells. Hypergeometric testing p values are shown in the table.

(j) Splicing factor motif analysis from downstream exon of altered SE in $WASP^{null+L425fs}$ B cells. Hypergeometric testing p values are shown in the table.

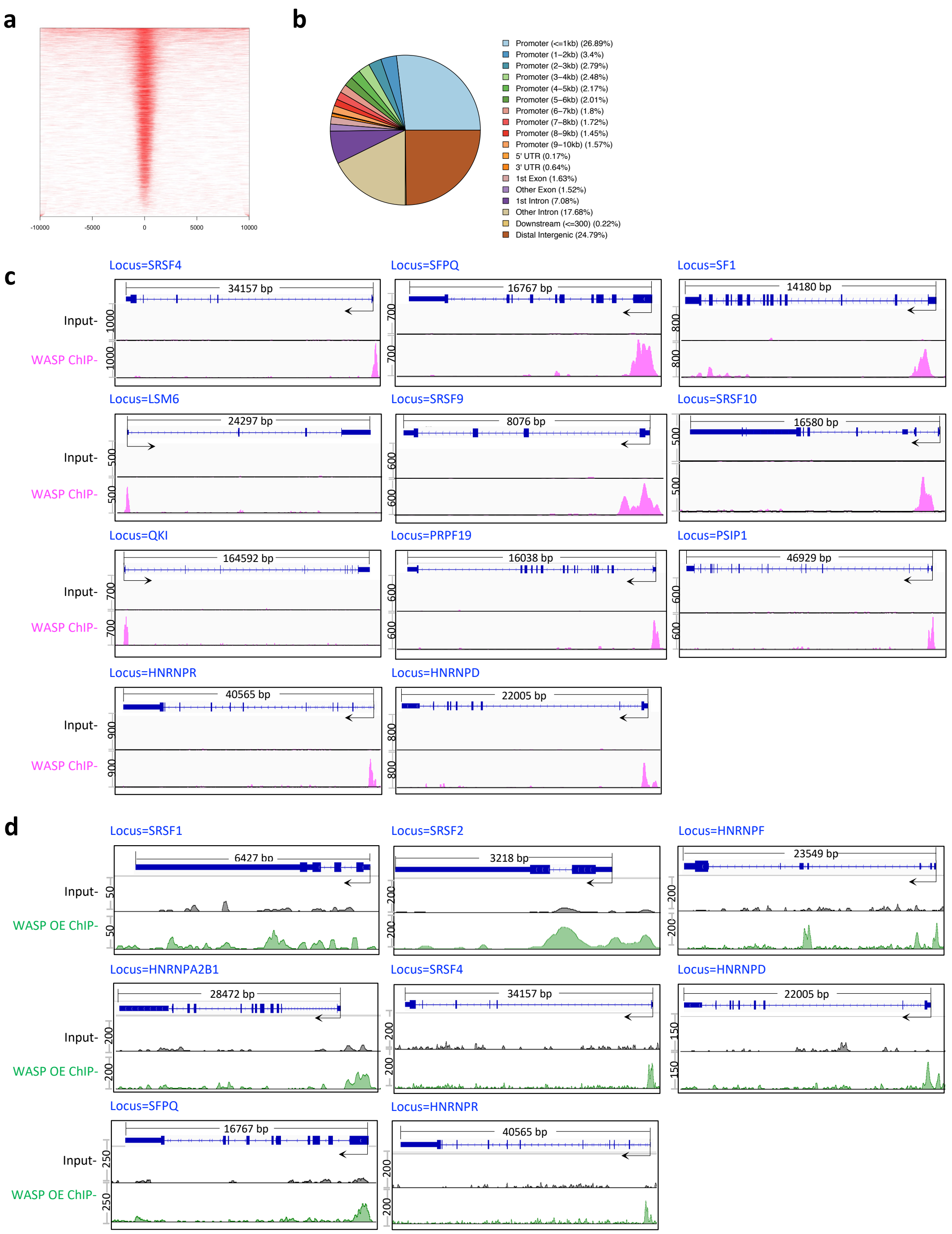

Top 5 de novo motifs from upregulated splicing factors associate with WASP binding

e

	Motif	p-value	Target %	Background %	Fold enrichment	Best known match
1	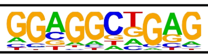	1e-25	61.26	12.05	5.1	ZNF460 (ZF)
2	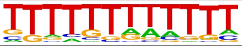	1e-24	33.33	1.11	3.0	SeqBias
3	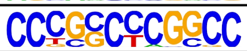	1e-20	37.84	3.52	10.8	KLF15 (ZF)
4	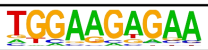	1e-18	40.54	5.59	7.3	ZNF189 (ZF)
5	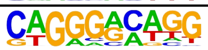	1e-17	63.06	19.88	3.2	PCBP3 (KH)

Fig. S7 Location of WASP ChIP peaks on splicing factor genes, related to Fig. 5-6.

(a) Heatmap of WASP ChIPseq peaks in TSS regions in the genome of WAS^{null} B cells overexpressing wild-type WASP cDNA. X-axis is shown in bp.

(b) Pie chart visualizing genomic annotation of WASP ChIPseq peaks from WAS^{null} B cells overexpressing wild-type WASP.

(c) Location of WASP ChIPseq peaks of WT B cells in SF genes shown in Integrative Genomics Viewer (IGV).

(d) Location of WASP ChIPseq peaks of WASP-overexpressing WAS^{null} B cells in SF genes shown in IGV.

(e) Top 5 de novo motifs in the promoter regions of upregulated SFs bound by WASP. Hypergeometric testing p values are shown in the table.

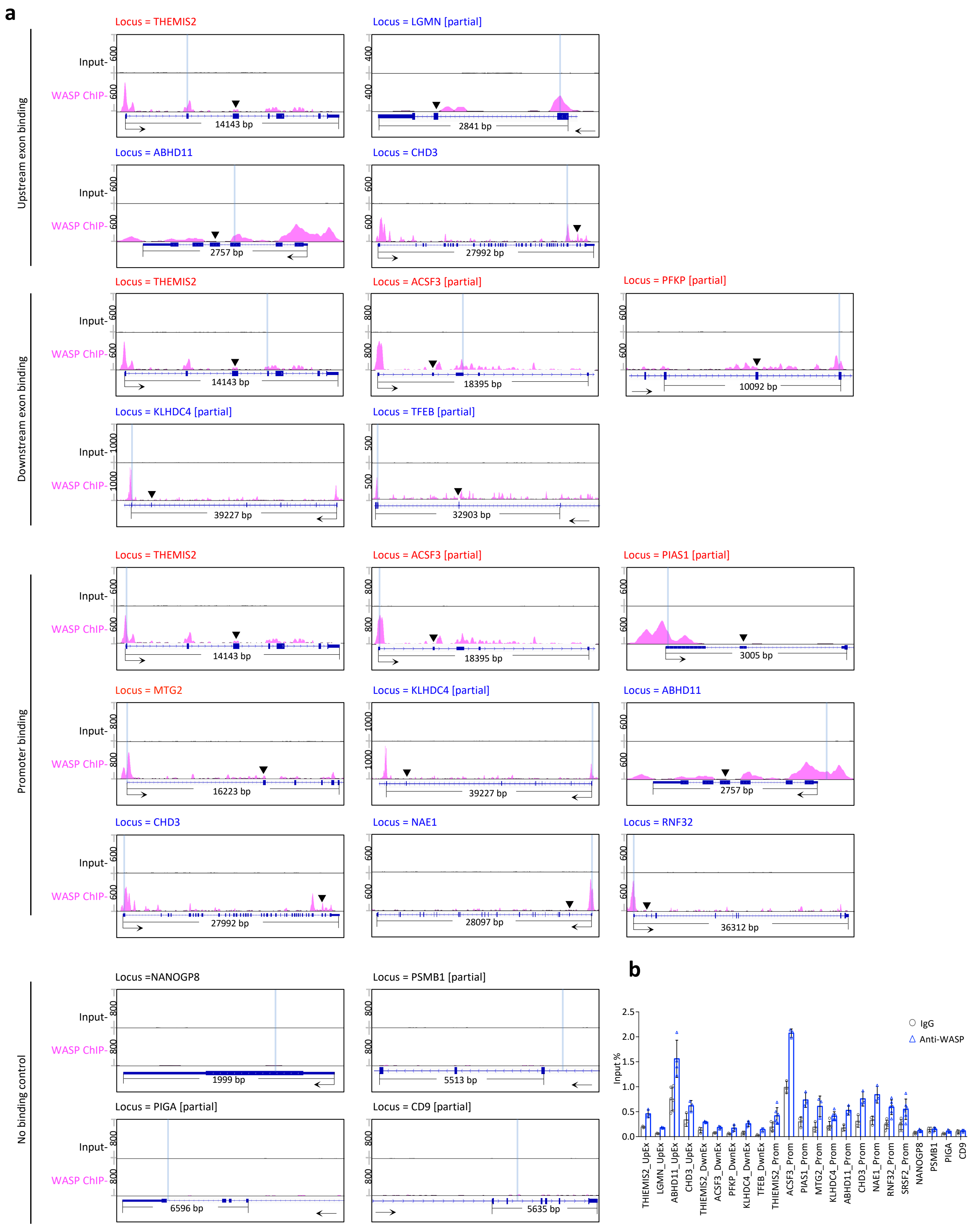
a
Upstream exon binding
Downstream exon binding
Promoter binding
No binding control
Input-
WASP ChIP-
Locus = THEMIS2
Locus = LGMN [partial]
Locus = ABHD11
Locus = CHD3
Locus = ACSF3 [partial]
Locus = PFKP [partial]
Locus = KLHDC4 [partial]
Locus = TFEB [partial]
Locus = PIAS1 [partial]
Locus = MTG2
Locus = NAE1
Locus = RNF32
Locus =NANOGP8
Locus = PSMB1 [partial]
Locus = PIGA [partial]
Locus = CD9 [partial]
14143 bp
2841 bp
2757 bp
27992 bp
18395 bp
10092 bp
39227 bp
32903 bp
3005 bp
16223 bp
28097 bp
36312 bp
1999 bp
5513 bp
6596 bp
5635 bp
b
Input %
IgG
Anti-WASP
THIEMIS2_UpEx
LGMN_UpEx
ABHD11_UpEx
CHD3_UpEx
THIEMIS2_DwnEx
ACSF3_DwnEx
PFKP_DwnEx
KLHDC4_DwnEx
TFEB_DwnEx
THIEMIS2_Prom
ACSF3_Prom
PIAS1_Prom
MTG2_Prom
KLHDC4_Prom
ABHD11_Prom
CHD3_Prom
NAE1_Prom
RNF32_Prom
SRSF2_Prom
NANOGP8
PSMB1
PIGA
CD9

Fig. S8 ChIP-qPCR Validation for WASP ChIPseq data, related to Fig. 5-6 and Fig. S7.

(a) Location of WASP ChIPseq peaks of WT B cells in selected AASE genes shown in IGV. The input track is shown above the corresponding WASP ChIPseq track using the same scale. Black triangle indicates the cassette exon; light blue vertical line indicates the center of the qPCR amplicon; elbow arrow below the gene models shows the transcription start site and the direction of transcription; straight arrow in partial gene view shows the direction of transcription; red gene name indicates more inclusion of the cassette exon in WASP deficient cells, blue more exclusion, and black negative control.

(b) ChIP-qPCR validation of WASP ChIPseq data for genes in Fig. S8a, S7d and Fig. 5c. UpEx: upstream exon. DwnEx: downstream exon. Prom: Promoter. WASP ChIP was independently repeated twice in WT B cells and once in WASP-overexpressing WAS^{null} B cells. The qPCR was performed in triplicate per sample, data are shown as mean ± SD, analyzed loci n = 23.

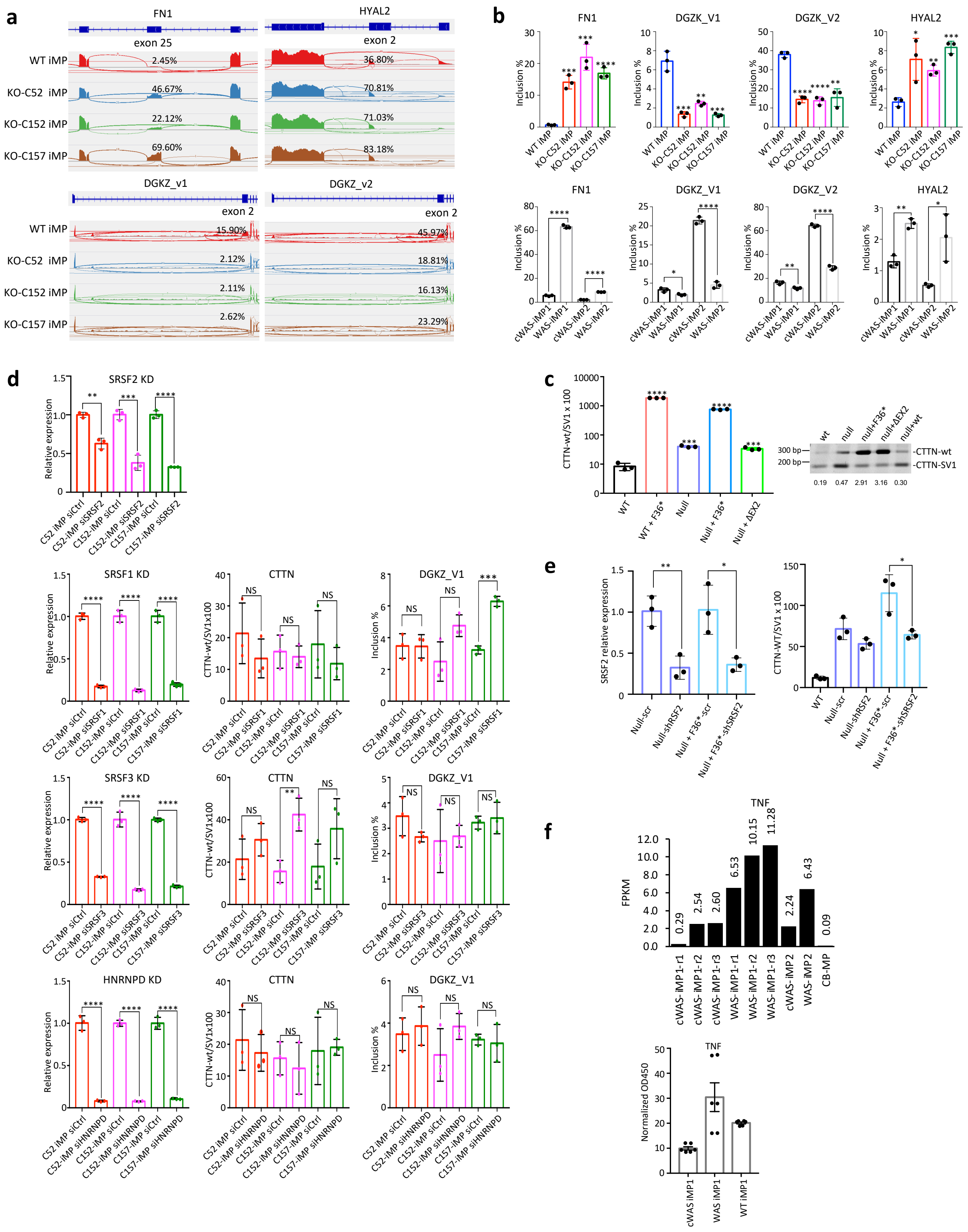
a
FN1
HYAL2
exon 25
exon 2
WT iMP
KO-C52 iMP
KO-C152 iMP
KO-C157 iMP
2.45%
46.67%
22.12%
69.60%
36.80%
70.81%
71.03%
83.18%
DGKZ_v1
DGKZ_v2
15.90%
2.12%
2.11%
2.62%
45.97%
18.81%
16.13%
23.29%
b
FN1
DGZK_V1
DGZK_V2
HYAL2
Inclusion %
WT iMP
KO-C52 iMP
KO-C152 iMP
KO-C157 iMP
DGKZ_V1
DGKZ_V2
cWAS-iMP1
WAS-iMP1
cWAS-iMP2
WAS-iMP2
c
CTTN-wt/SV1 x 100
WT
WT + F36*
Null
Null + F36*
Null + ΔEX2
wt
null
null+F36*
null+ΔEX2
null+wt
300 bp
200 bp
-CTTN-wt
-CTTN-SV1
0.19
0.47
2.91
3.16
0.30
d
SRSF2 KD
SRSF1 KD
SRSF3 KD
HNRNPD KD
CTTN
DGKZ_V1
Relative expression
CTTN-wt/SV1x100
Inclusion %
NS
e
SRSF2 relative expression
CTTN-WT/SV1 x 100
Null-scr
Null-shRSF2
Null + F36*-scr
Null + F36*-shSRSF2
Null-shSRSF2
f
TNF
FPKM
0.29
2.54
2.60
6.53
10.15
11.28
2.24
6.43
0.09
cWAS-iMP1-r1
cWAS-iMP1-r2
cWAS-iMP1-r3
WAS-iMP1-r1
WAS-iMP1-r2
WAS-iMP1-r3
cWAS-iMP2
WAS-iMP2
CB-MP
Normalized OD450
cWAS iMP1
WAS iMP1
WT iMP1

Fig. S9 Validation of AASEs and rescue of WAS phenotype, related to Fig. 5.

(a) Sashimi plot of selected AASEs in genes associated with the GSEA immunity gene set in WAS mutant iMPs.

(b) Exon-specific qPCR validation for the selected AASEs shown in (a). Biological replicate n = 3. Data are shown as mean ± SD. Two-sided Student's t-test. *FN1*: ***p = 0.0004 (KO-C52 iMP), ***p = 0.0008 (KO-C152 iMP), ****p < 0.0001; *DGZK*_V1: *p = 0.0192, **p = 0.002, ***p = 0.0009 (KO-C52 iMP), ***p = 0.0007 (KO-C157 iMP), ****p < 0.0001; *DGZK*_V2: **p = 0.0013 (KO-C157 iMP), **p = 0.0079 (WAS-iMP1), ****p < 0.0001; *HYAL2*: *p = 0.0262 (KO-C52 iMP), *p = 0.0251 (WAS-iMP2), **p = 0.0015 (KO-C152 iMP), **p = 0.001 (WAS-iMP1), ***p = 0.0002.

(c) Exon-specific qPCR validation for the CTTN AASE in B cell lines. Biological replicate n = 3. Data are shown as mean ± SD. Two-sided Student's t-test, ***p = 0.0001 (Null), ***p = 0.0005 (Null + ΔEX2), ****p < 0.0001.

(d) Left column: qPCR validation of the knockdown of SFs. Middle and right columns: exon-specific qPCR analysis of AASEs in after siRNA mediated knockdown of upregulated splicing factors (*SRSF1*, *SRSF2*, *SRSF3*, *HNRNPD*) in KO iMPs, related to Fig. 5h. Biological replicate n = 3. Data are shown as mean ± SD. Two-sided Student's t-test, **p = 0.0011 (*SRSF2*), **p = 0.0076 (*CTTN*), ***p = 0.0008 (*SRSF2*), ***p = 0.0002 (*DGKZ*_V1), ****p < 0.0001.

(e) *CTTN* splicing is rescued by knocking down *SRSF2* in WASP mutant (null and null+F36*) B cells. Biological replicate n = 3. Data are shown as mean ± SD. Two-sided Student's t-test, *SRSF2*: *p = 0.0204, **p = 0.0069: *CTTN*: *p = 0.0188.

(f) TNF expression levels in WAS iMPs and cWAS iMPs. Top: The RNAseq data of TNF mRNA expression level, biological replicate for WAS-iMP1 n = 3, for cWAS-iMP1 n = 3, for WAS-iMP2 n = 1, for cWAS-iMP2 n = 1, for CB-iMP n = 1. Bottom: ELISA quantitation of secreted TNF in the indicated macrophages upon stimulation with lipopolysaccharide (LPS). Values shown are as mean ± SEM. Biological replicate n =3.

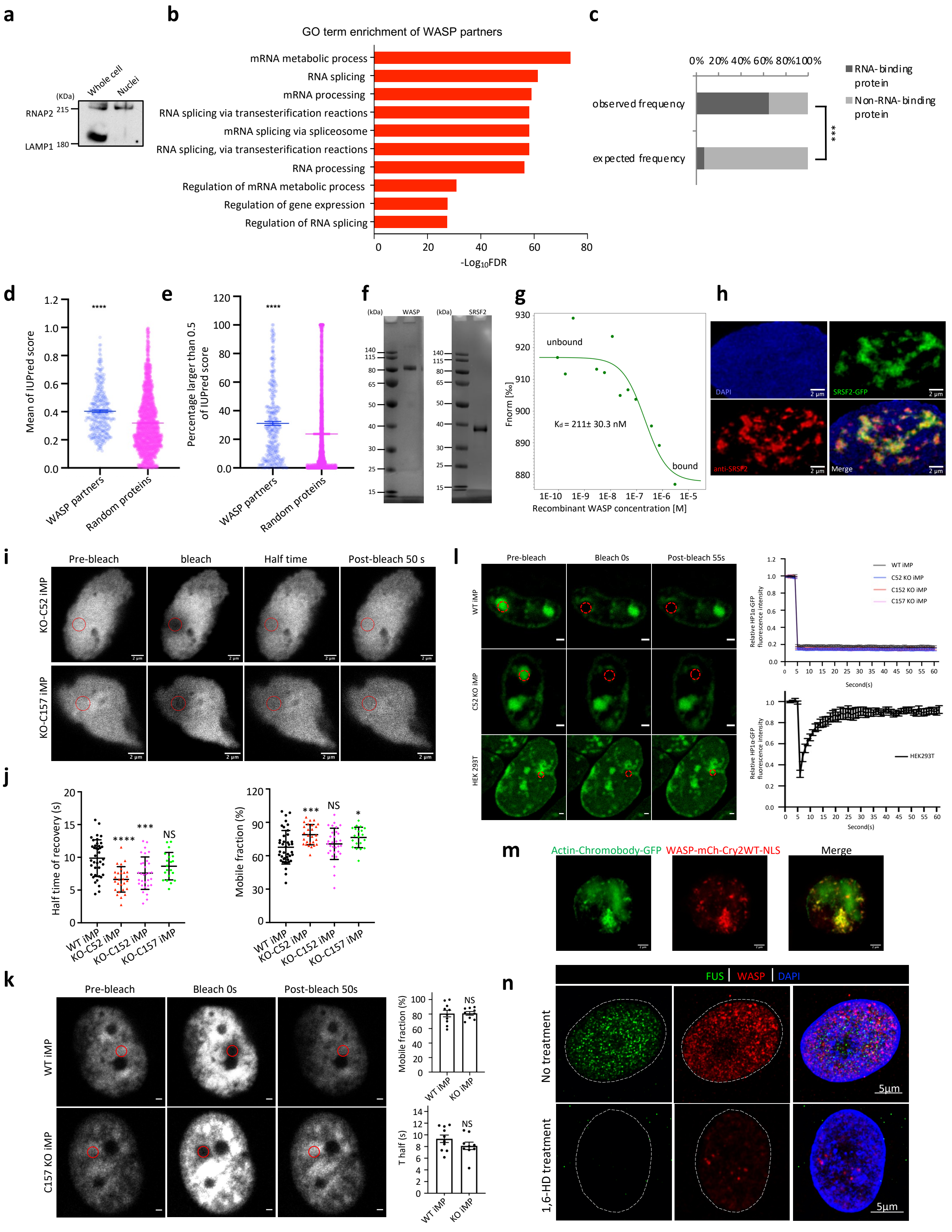

a
b
c
GO term enrichment of WASP partners
mRNA metabolic process
RNA splicing
mRNA processing
RNA splicing via transesterification reactions
mRNA splicing via spliceosome
RNA splicing, via transesterification reactions
RNA processing
Regulation of mRNA metabolic process
Regulation of gene expression
Regulation of RNA splicing
-Log10FDR
Whole cell
Nuclei
RNAP2
LAMP1
observed frequency
expected frequency
RNA-binding protein
Non-RNA-binding protein
d
e
f
g
h
Mean of IUPred score
Percentage larger than 0.5 of IUPred score
WASP partners
Random proteins
WASP
SRSF2
unbound
bound
Kd = 211± 30.3 nM
Fnorm [‰]
Recombinant WASP concentration [M]
DAPI
SRSF2-GFP
anti-SRSF2
Merge
i
Pre-bleach
bleach
Half time
Post-bleach 50 s
KO-C52 iMP
KO-C157 iMP
l
Bleach 0s
Post-bleach 55s
WT iMP
C52 KO iMP
HEK 293T
j
Half time of recovery (s)
Mobile fraction (%)
KO-C52 iMP
KO-C152 iMP
KO-C157 iMP
m
Actin-Chromobody-GFP
WASP-mCh-Cry2WT-NLS
k
Post-bleach 50s
C157 KO iMP
T half (s)
KO iMP
n
FUS
WASP
No treatment
1,6-HD treatment
5μm

Fig. S10 Characterization of WASP binding partners and WASP condensates, related to Fig. 6-7.

(a) Western blot analysis of lysosomal-associated membrane protein 1 (LAMP1) and RNA polymerase II (RNAP2) in whole cell and nuclei lysates. The experiment was done once.

(b) GO term enrichment of 99 WASP partners.

(c) Quantitation of known RNA-binding proteins (n=200) in the WASP interactome. $\chi 2$ test, ****p*<0.0001.

(d, e) Mean IUPred score (d) and the percentage of proteins with IUPred score>0.5 (e) in 342 WASP partners and 3000 random proteins. Data are shown as mean ± SEM. Mann Whitney test, *****p* < 0.0001.

(f) Representative Coomassie-stained SDS-PAGE images of full-length human WASP and SRSF2. The experiment was repeated 3 times independently.

(g) MST of the binding interaction between SRSF2 and WASP. The k_d value is shown as mean ± SEM, biological replicate n=3.

(h) Representative confocal images of exogenous SRSF2-GFP and antibody staining of SRSF2. Scale bar = 2 μm. The experiment was repeated twice independently.

(i-k) Representative FRAP images (i) and quantitative analysis (j) of the half time of FRAP recovery and mobile fraction of SRSF2-GFP fluorescence. Scale bar = 2 μm. Two-sided Student's t-test. Data are shown as mean ± SD. Biological replicate n=3, **p* = 0.0195, ****p* < 0.0006 (KO-C152 iMP), ****p* = 0.0009 (KO-C52 iMP), *****p* < 0.0001. The experiment was repeated 3 times independently. In (k), cells were bleached at the positions with a clear speckled GFP signal. Scale bar = 1 μm. n=10 for WT iMPs, 9 for KO iMPs. Data are shown as mean ± SD. Two-sided Student's t-test, NS: not significant.

(l) Representative FRAP images and time-lapse FRAP curves of HP1α-GFP fluorescence. n=15 (WT and KO iMPs), 8 (HEK293T). Data are shown as mean ± SEM. Scale bar = 1 μm.

(m) Live-cell confocal images of WASP and nuclear actin (GFP-tagged nuclear actin chromobody) in HEK293 cells. Scale bar = 2 μm. The experiment was repeated 3 times independently.

(n) Immunofluorescence of FUS and endogenous WASP in WT iMPs with or without 1,6-HD treatment. Scale bar = 5 μm. The experiment was repeated 3 times independently.

Source data for d, e, j, and k are provided as a Source Data file.

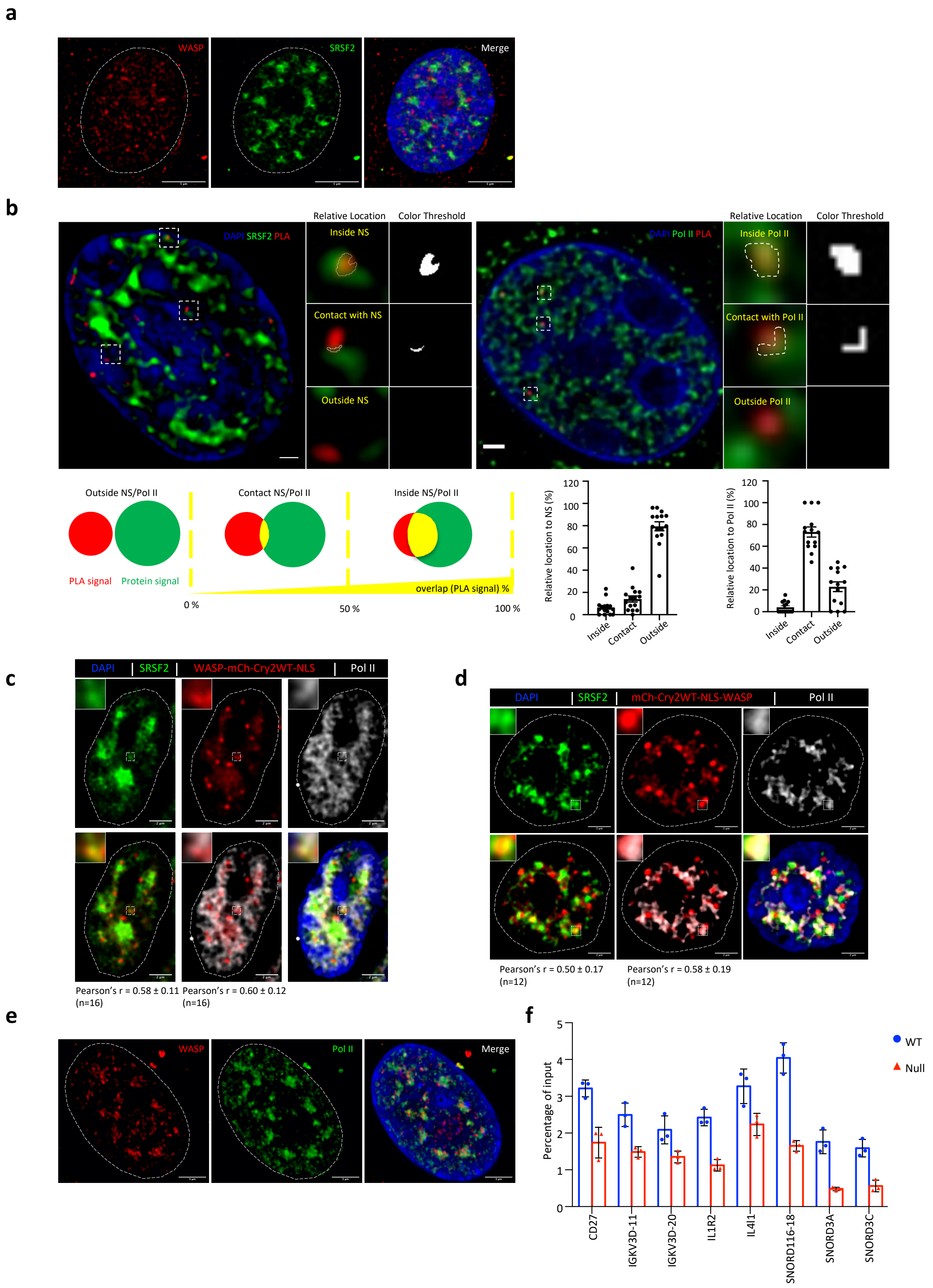
a
WASP
SRSF2
Merge
b
DAPI SRSF2 PLA
Relative Location
Color Threshold
Inside NS
Contact with NS
Outside NS
DAPI Pol II PLA
Inside Pol II
Contact with Pol II
Outside Pol II
Outside NS/Pol II
Contact NS/Pol II
Inside NS/Pol II
PLA signal
Protein signal
overlap (PLA signal) %
0 %
50 %
100 %
Relative location to NS (%)
Relative location to Pol II (%)
Inside
Contact
Outside
c
DAPI
SRSF2
WASP-mCh-Cry2WT-NLS
Pol II
Pearson's r = 0.58 ± 0.11 (n=16)
Pearson's r = 0.60 ± 0.12 (n=16)
d
DAPI
SRSF2
mCh-Cry2WT-NLS-WASP
Pol II
Pearson's r = 0.50 ± 0.17 (n=12)
Pearson's r = 0.58 ± 0.19 (n=12)
e
WASP
Pol II
Merge
f
Percentage of input
WT
Null
CD27
IGKV3D-11
IGKV3D-20
IL1R2
IL4I1
SNORD116-18
SNORD3A
SNORD3C

Fig. S11 Colocalization of WASP with SRSF2, Pol II and nascent RNA related to Fig. 7.

(a) Representative maximum projection of confocal images of endogenous WASP. Scale bar = 5 μm. The experiment was repeated at least 3 times independently.

(b) Upper: representative single-plane confocal images of PLA signals for WASP-SRSF2 and SRSF2 immunofluorescence signals (left) and for WASP-Pol II and Pol II immunofluorescence signals (right). $SRSF2^{bright}$ foci label nuclear speckles (NS). Insets: magnified view of boxed locations. Lower left: schematic diagrams of the categories for quantitative analysis. We define no overlap between PLA signals and SRSF2 or Pol II signals as "outside", 0-50% overlap signals to total PLA signals as "contact", and 50-100% as "inside". Lower right: statistics of the relative location of PLA signals to SRSF2 or Pol II signals. 487 PLA signals from 15 cells were quantified for the relative location to NSs. 167 PLA signals from 15 cells were quantified for the relative location to Pol II. Data are shown as mean ± SEM.

(c, d) Representative single-plane confocal images and colocalization analysis of optoWASP droplets (N-terminal WASP, c) and optoWASP droplets (C-terminal WASP, d) with endogenous SRSF2 and active Pol II, respectively. Scale bar = 2 μm. Insets show the framed region in the images. The Pearson correlation coefficient of colocalization (Pearson's r) value was calculated using the Leica LAS X software. Analyzed image n = 16 in c, n = 12 in d, data are shown as mean ± SD.

(e) Representative maximum Z-projection of confocal images of endogenous WASP co-stained with Pol II. Scale bar = 3 μm. The experiment was repeated twice independently.

(f) Selected RNA immunoprecipitation (RIP) events validated by qPCR. RIP was independently repeated twice. The qPCR was performed in triplicate per sample, data are shown as mean ± SD, analyzed loci n = 8.

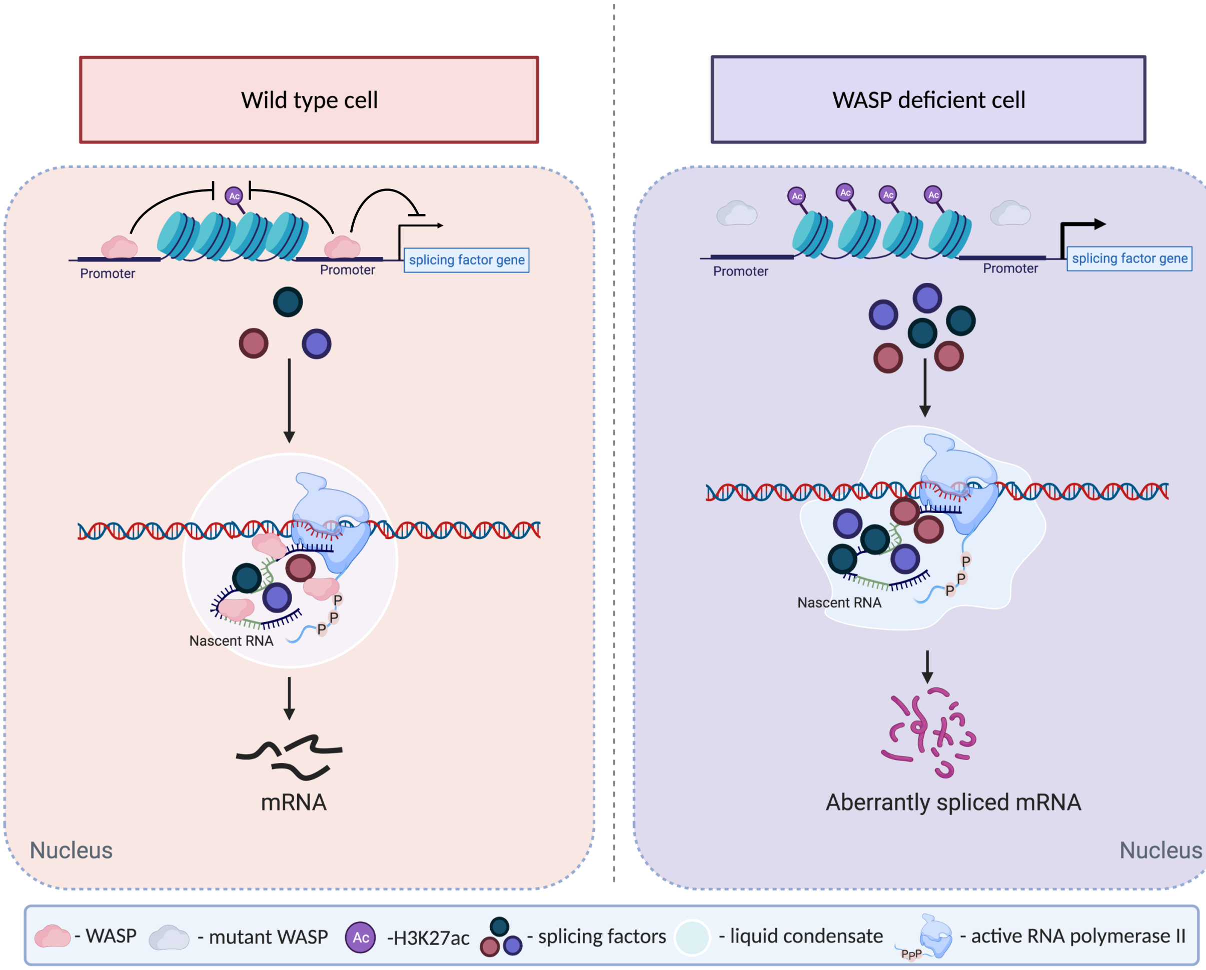

Fig. S12 Models of the mechanisms by which WASP regulates splicing.

Created with BioRender.com
