# Supplementary material for: Wiskott-Aldrich syndrome protein forms nuclear condensates and regulates alternative splicing
Source: Nat Commun. 2022 Jun 25;13:3646. doi: 10.1038/s41467-022-31220-8 (PMC9233711; doi:10.1038/s41467-022-31220-8)
Supplement: Supplementary file 2 — Description of Additional Supplementary Files [file 41467_2022_31220_MOESM2_ESM.pdf]

Description of Additional Supplementary Files

File name: Supplementary Data 1
Description: List of upregulated splicing factors in WAS KO-iMPs from DIA-MS data.

File name: Supplementary Data 2
Description: List of AASE in genes associated with immunity.

File name: Supplementary Data 3
Description: List of WASP partners identified by MudPIT.

File name: Supplementary Data 4
Description: List of genes identified by RIPseq.

File name: Supplementary Data 5
Description: List of oligonucleotides.

File name: Supplementary Data 6
Description: List of antibodies.

File name: Supplementary Data 7
Description: Gating strategy for FACS plots.

File name: Supplementary Movie 1
Description: A time-lapse movie of optoWASP activated by blue light.

File name: Supplementary Movie 2
Description: A time-lapse movie of N-terminal IDR of WASP fused with Cry2 optogenetic tool activated by blue light.

File name: Supplementary Movie 3
Description: A time-lapse movie of C-terminal IDR of WASP fused with Cry2 optogenetic tool activated by blue light.

File name: Supplementary Movie 4
Description: A time-lapse movie of optoWASP droplet fusion.
