# Supplementary material for: Wiskott-Aldrich syndrome protein forms nuclear condensates and regulates alternative splicing
Source: Nat Commun. 2022 Jun 25;13:3646. doi: 10.1038/s41467-022-31220-8 (PMC9233711; doi:10.1038/s41467-022-31220-8)
Supplement: Supplementary file 7 — Supplementary Data 5 [file 41467_2022_31220_MOESM7_ESM.docx]

Table S5 Oligonucleotides

| Oligonucleotides | |
| --- | --- |
| H-SFPQ right: CCCATTCCTCTAGGACCCTG | This paper |
| h-SFPQ left: GTGGTTCCATGATGGGAAGT | This paper |
| h-HNRNPA3 left: CAACTGCTCTGGTTCCTTTG | This paper |
| h-HNRNPA3 right: ATGGAGGTAAAACCGCCG | This paper |
| h-GATA2-F: GGCCCACTCTCTGTGTACC | This paper |
| h-GATA2-R: CATCTTCATGCTCTCCGTCAG | This paper |
| h-RUNX1-F: TCGAAGACATCGGCAGAAACT | This paper |
| h-RUNX1-R: GAGGCTGAGGGTTAAAGGCA | This paper |
| h-GAPDH-F: GCACCGTCAAGGCTGAGAAC | This paper |
| h-GAPDH-R: AGGGATCTCGCTCCTGGAA | This paper |
| h-HoxB4-F: GTGAGCACGGTAAACCCCAAT | This paper |
| h-HoxB4-R: CGAGCGGATCTTGGTGTTG | This paper |
| h-CD45-F: ACAGCCAGCACCTTTCCTAC | This paper |
| h-CD45-R: GTGCAGGTAAGGCAGCAGA | This paper |
| h-CD34-F: GCGCTTTGCTTGCTGAGTTT | This paper |
| h-CD34-R: GCCATGTTGAGACACAGGGT | This paper |
| h-CD31-F: AACAGTGTTGACATGAAGAGCC | This paper |
| h-CD31-R: TGTAAAACAGCACGTCATCCTT | This paper |
| h-SRSF1-left: AATGGCGCCTTTATCAGCTC | This paper |
| h-SRSF1-right: GTGCGTGTTGGGATCGAATC | This paper |
| h-SRSF2-left: GAAAGCGAACGAAGGCGAAG | This paper |
| h-SRSF2-right: GTGGACAACCTGACCTACCG | This paper |
| h-HNRNPF-left: TTTCAGATCGAGCTTGCACG | This paper |
| h-HNRNPF-right: CCCGTCCAACCTGTATCACT | This paper |
| h-HNRNPA2B1-left: AAGCTGTTTGTACGCTCAGG | This paper |
| h-HNRNPA2B1-right: GCAGGAAATCGGACCAATTCAG | This paper |
| h-B2M-left: TGCTGTCTCCATGTTTGATGTATCT | This paper |
| h-B2M-right: TCTCTGCTCCCCACCTCTAAGT | This paper |
| h-PCBP2 F: GTTGGCAATGCAACAGTCTC | This paper |
| h-PCBP2 SE probe: ACCGGATTCAGTGCAGGTTTGGAT | This paper |
| h-PCBP2 Inc probe: AGGTGAAAGGCTATTGGGCAGGTT | This paper |
| h-PCBP2 R: ATGGTGAGTTCATGAGAAGTAGTC | This paper |
| h-SENP1 F: CATGTTCCACAAGAAGTGCAG | This paper |
| h-SENP1 SE probe: ACTCAG﻿ATAATCCTTCCTCAGACAGT | This paper |
| h-SENP1 Inc probe: TGTGGCAGGATTCAGCTCCTTCAT | This paper |
| h-SENP1 R: AATCGCCTGAGCCAAGAAA | This paper |
| h-TPD52L2 F: AGCTGAAACAGAACCTGTCC | This paper |
| h-TPD52L2 SE probe: TCTAGCGCCTACAAGAAGACTCAGGA | This paper |
| h-TPD52L2 Inc probe: ﻿TGAGAAAGTGACCCAGTCAGACCTCT | This paper |
| h-TPD52L2 R: CTTCTGTCCTGCCTGTGAAA | This paper |
| h-TCF12 F: AGGGCAAGCACCTTCATC | This paper |
| h-TCF12 SE probe: AAACTCACTCCACTCCCTGCAGTC | This paper |
| h-TCF12 Inc probe: TCGAGTTGAGCAGCAACTTCACGA | This paper |
| h-TCF12 R: GTCTGTCTAAACGATCCTCCATT | This paper |
| h-HMG20A F: GGAATTGTCCTCTGTGGAAGG | This paper |
| HMG20A SE probe: CCCTGTCGTCAGCAGAGAGATGGA | This paper |
| h-HMG20A Inc probe: TGTCGTCAGCAGAGTCTCTACAAGGA | This paper |
| h-HMG20A R: GGTAGGGTGGAGCTAGTCAT | This paper |
| CD27 F: AGCCCACCCACTTACCTTATG | This paper |
| CD27 R: ACAAGGATGCGAATAAAATCGGA | This paper |
| IGKV3D-11 F: TAGGACCCAGACGGAACCAT | This paper |
| IGKV3D-11 R: ATCTGGGAGCCAGAGTAGCA | This paper |
| IGKV3D-20 F: GGGGCCAGTCAGAGTGTTAG | This paper |
| IGKV3D-20 R: GAAGTCTGTCCCAGACCCAC | This paper |
| IL1R2 F: ATGTTGCGCTTGTACGTGTTG | This paper |
| IL1R2 R: CCCGCTTGTAATGCCTCCC | This paper |
| IL4I1 F: GCCAAGACCCCTTCGAGAAAT | This paper |
| IL4I1 R: CCGATCCTGTTATCTGCCTCC | This paper |
| SNORD116-18 F: TCCTTGGAAAGCTGAACAAAATGA | This paper |
| SNORD116-18 R: ACCTCAGTTCGACGAGGATG | This paper |
| SNORD3A F: TCTTGGGTTTTCTCGGGGTG | This paper |
| SNORD3A R: GACAATAGGAGGTGCCACACA | This paper |
| SNORD3C F: TCTTGGGTTTTCTCGGGGTG | This paper |
| SNORD3C R: GACAATAGGAGGTGCCACACA | This paper |
| RT-FN1 skEX F: TACCATCATCCCAGCTGTTC | This paper |
| RT-FN1 skEX R: GCACCAGGAAGTTGGTTAAATC | This paper |
| RT-FN1 incEX F: TTGTGGACTCCTCAGTAGGATA | This paper |
| RT-FN1 incEX R: GAGGAGGAACAGCCGTTT | This paper |
| RT-DGKZ_V1 skEX F: CCCAGACACAGAAAGCCA | This paper |
| RT-DGKZ_V1 skEX R: TCCACTGTACTCCGGATCT | This paper |
| RT-DGKZ_V1 incEX F: AAGAGCCTCAGGCACCA | This paper |
| RT-DGKZ_V1 incEX R: TGATGGCTTTCGCGAGC | This paper |
| RT-DGKZ_V2 skEX F: TTCGGGCACAGGAAAGC | This paper |
| RT-DGKZ_V2 skEX R: GTCCACTGTACTCCGGATCT | This paper |
| RT-DGKZ_V2 incEX F: AAGAGCCTCAGGCACCA | This paper |
| RT-DGKZ_V2 incEX R: GTGATGGCTTTCGCGAGC | This paper |
| RT-HYAL2 skEX F: AAGTAGCCTAGCTGGAGAGG | This paper |
| RT- HYAL2 skEX R: CCAGCTCAGGAACTGTGTG | This paper |
| RT- HYAL2 incEX F: ACTCCCACACAGGGCTG | This paper |
| RT- HYAL2 incEX R: CTCTGAGCCGGGCTTTCTAT | This paper |
| THEMIS2_Prom F: CCCTCAGTCTGAGCCCAGA | This paper |
| THEMIS2_Prom R: CGCTCACCCTCGAAGTAGAC | This paper |
| THEMIS2_UpEx F: AATGAGTGCTGCCTCTCCAC | This paper |
| THEMIS2_UpEx R: TTCGGGTTCTCACAGACCAC | This paper |
| THEMIS2_DwnEx F: TATCAAACCGCTGCTGCTGA | This paper |
| THEMIS2_DwnEx R: CCCTCAGGAACCTCCAGGAT | This paper |
| ACSF3_Prom F: GAACTGGTCCGGCCCGAC | This paper |
| ACSF3_Prom R: CAACTCTTCCTCGTCCTCCG | This paper |
| ACSF3_DwnEx F: GGGCGCCATGATCATCTACA | This paper |
| ACSF3_DwnEx R: CAGCCCTGATGTTTTGGTGC | This paper |
| PIAS1_Prom F: GAAGTTCACTGCGCTTGCG | This paper |
| PIAS1_Prom R: GTCTCCGCGGCCGAATATTA | This paper |
| MTG2_Prom F: GGCGTGTTTTCTGCACCT | This paper |
| MTG2_Prom R: TCGGGAAAGCACGTCACT | This paper |
| PFKP_DwnEx F: GCTGGAGTGACTGATCGCTT | This paper |
| PFKP_DwnEx R: GCCCATGGTCTCGATGATGA | This paper |
| NAE1_Prom F: TCCTTGAGCAGCTTTCCCAG | This paper |
| NAE1_Prom R: GAGACGGTAGCGGCGAAG | This paper |
| RNF32_Prom F: GGGAGTTGCCTGAGGGAAAA | This paper |
| RNF32_Prom R: CACCCTTCTTTCCGTCCCTC | This paper |
| KLHDC4_Prom F: TTGCCCTTCTTGCCCATCTT | This paper |
| KLHDC4_Prom R: GTGACTCCATTTCTGTGCGC | This paper |
| KLHDC4_DwnEx F: CCGCCTTACCTGTTTCGAGT | This paper |
| KLHDC4_DwnEx R: GGACACCTTCACATGGAGCA | This paper |
| TFEB_DwnEx F: GCCCTACCTTCAACACCTCC | This paper |
| TFEB_DwnEx R: GCAACAGGCTGTCATGCATT | This paper |
| LGMN_UpEx F: GGAGTGCCAGTTGAAGCAGT | This paper |
| LGMN_UpEx R: AAGATCGTCTCCTTGCTGGC | This paper |
| ABHD11_Prom F: GCCCCTTACGCCATTACGTA | This paper |
| ABHD11_Prom R: TTCCCAGATTCTACGCACCG | This paper |
| ABHD11_UpEx F: AGCATGGCTGTCTTTCCTCC | This paper |
| ABHD11_UpEx R: CGAGATCATGAGCCAGGACC | This paper |
| CHD3_Prom F: TGGTGTCGGAGGAGGAAGAA | This paper |
| CHD3_Prom R: AGTACTCCCTCGTCGTCGTC | This paper |
| CHD3_UpEx F: AGAGCCCTACCTTCACCTCC | This paper |
| CHD3_UpEx R: CAGCTGCTCCTCAATCACCA | This paper |
| SRSF2_Prom F: AATGGCGCCTTTATCAGCTC | This paper |
| SRSF2_Prom R: GTGCGTGTTGGGATCGAATC | This paper |
| NANOGP8 F: TTTGTGGGCCTGAAGAAAACT | This paper |
| NANOGP8 R: AGGGCTGTCCTGAATAAGCAG | This paper |
| PSMB1 F: GATATGAGACTGTGGGTAAGT | This paper |
| PSMB1 R: GATCCTTTGAACACCCTAATG | This paper |
| PIGA F: CGGTGTCTTGCCCAGAATAA | This paper |
| PIGA R: CATCACCCCATCACCTGTC | This paper |
| CD9 F: TTCATAGAGAAGGGAGCCGT | This paper |
| CD9 R: GCCTCCACTAACTTTCCTCC | This paper |
