# Supplementary material for: Wiskott-Aldrich syndrome protein forms nuclear condensates and regulates alternative splicing
Source: Nat Commun. 2022 Jun 25;13:3646. doi: 10.1038/s41467-022-31220-8 (PMC9233711; doi:10.1038/s41467-022-31220-8)
Supplement: Supplementary file 8 — Supplementary Data 6 [file 41467_2022_31220_MOESM8_ESM.docx]

| **ANTIBODIES** | **SOURCE** | **IDENTIFIER** | **DILUTION** |
| --- | --- | --- | --- |
| Mouse monoclonal anti-beta Actin antibody | Abcam | ab8227 | WB: 1000x |
| Mouse monoclonal anti-WASP antibody (5A5) | BD Biosciences | Cat# 557773 | IP: 3 µg |
| Rabbit polyclonal anti-WASP antibody (H250) | Santa Cruz Biotechnology | Cat# sc-8353 | WB: 200x |
| Rabbit IgG, polyclonal - Isotype Control | Abcam | ab37415 | PLA:100x  ChIP: 5 µg |
| Mouse IgG1, Kappa Monoclonal | Abcam | ab91353 | ChIP: 10 µg |
| Anti-SC35 antibody | Abcam | ab204916 | PLA: 100x |
| Anti-SC35 antibody | GeneTex | GXT11826 | IF: 500x  WB:1000x |
| Anti-phospho-RNA polymerase II | Abcam | ab5095 | IF: 100x  PLA: 500X |
| Rabbit polyclonal anti-Histone H3 (acetyl K27) antibody | Active Motif | 39133 | ChIP: 5 µg |
| Mouse monoclonal anti-human CD43-APC | BD Biosciences | Cat# 560198 | FACS: 50x |
| Mouse monoclonal anti-CD45-APC | BD Biosciences | Cat# 555485 | FACS: 50x |
| Mouse monoclonal anti-CD34-PE | BD Biosciences | Cat# 555822 | FACS: 50x |
| Mouse monoclonal anti-human CD3 PE | BD Biosciences | Cat# 555333 | FACS: 50x |
| Mouse monoclonal anti-CD11c PE | BD Biosciences | Cat# 560999 | FACS: 50x |
| Mouse monoclonal anti-CD20 FITC | BD Biosciences | Cat# 555622 | FACS: 50x |
| Mouse monoclonal anti-CD8 FITC | BD Biosciences | Cat# 557085 | FACS: 50x |
| Mouse monoclonal anti-CD4 PE | BD Biosciences | Cat# 560649 | FACS: 50x |
| Mouse monoclonal anti-TRA-1-60 PerCP-Cy^TM^5.5 | BD Biosciences | Cat# 561573 | FACS: 100x |
| Mouse monoclonal anti-TRA-1-81 PerCP-Cy^TM^5.5 | BD Biosciences | Cat# 561575 | FACS: 50x |
| Mouse monoclonal isotype control APC | BD Biosciences | Cat# 555751 | FACS: 50x |
| Mouse monoclonal isotype control PE | BD Biosciences | Cat# 555749 | FACS: 50x |
| Mouse monoclonal isotype control FITC | BD Biosciences | Cat# 555742 | FACS: 50x |
| Mouse monoclonal anti-CD1a Alexa Fluor 700 | BioLegend | Cat# 300120 | FACS: 50x |
| Mouse monoclonal anti-CD14 PE | BioLegend | Cat# 301805 | FACS: 50x |
| Mouse monoclonal anti-human CD235a-PE | eBioscience | Cat# 12-9987-80 | FACS: 50x |
| Mouse monoclonal anti-human CD11b FITC | eBioscience | Cat# 11-0118-41 | FACS: 50x |
| Mouse monoclonal anti-human CD209 (DC-SIGN) | eBioscience | Cat# 17-2099-41 | FACS: 50x |
| Mouse monoclonal anti-human CD115 PE | eBioscience | Cat# 12-1159-41 | FACS: 50x |
| Mouse monoclonal anti-human CD38 eFluor 450 | eBioscience | Cat# 48-0388-42 | FACS: 50x |
| Mouse monoclonal anti-human CD34-APC | Miltenyi Biotec | Cat#130-090-954 | FACS: 50x |
| Mouse monoclonal anti-human CD34-PerCP Vio700 | Miltenyi Biotec | Cat# 130-097-915 | FACS: 50x |
| Mouse monoclonal anti-human CD163 FITC | Miltenyi Biotec | Cat# 130-099-969 | FACS: 50x |
| Mouse monoclonal anti-human HLA-DR APC | Miltenyi Biotec | Cat# 130-095-297 | FACS: 50x |
| Mouse monoclonal anti-human Tra-1-85 APC | R&D Systems | Cat# FAB3195A | FACS: 100x |
| Rabbit polyclonal anti-SF3B3 | Genetex | Cat# GTX106450 | PLA: 100x |
| Rabbit polyclonal anti-hnRNPA2B1 | Genetex | Cat# GTX127928 | PLA: 100x |
| Rabbit polyclonal anti-cortactin | Santa Cruz Biotechnology | Cat# sc-11408 | IF: 200x |
| Rabbit polyclonal anti-WASP | Abcam | ab74904 | IF: 200x  PLA: 200x |
| Goat polyclonal anti-Lamin B (M-20) | Santa Cruz Biotechnology | Cat# sc-6217 | IF: 200x |
| Rabbit monoclonal anti-N-WASP antibody | Cell Signaling Technology | Cat# 4848 | WB: 200x |
| Rabbit monoclonal anti-human anti-SF3B1 | Cell Signaling Technology | Cat# 14434 | IF: 300x |
| Rabbit polyclonal anti-Cyclophilin B antibody | Thermo Fisher | Cat# PA1027A | WB: 2000x |
| Mouse monoclonal anti-WASP antibody (D1) | Santa Cruz Biotechnology | **Cat**# sc-5300 | IF/PLA:100x  WB: 500x  ChIP:10µg |
| Mouse monoclonal anti-WASP antibody (B9) | Santa Cruz Biotechnology | Cat# sc-13139 | IF: 100x  PLA: 200x  WB: 500x |
| Rabbit polyclonal anti-FUS antibody | GeneTex | Cat# 101810 | IF: 200x |
| Anti-SC35 antibody | Abcam | ab11826 | IF/PLA: 100X |
| IgG (H+L) Cross-Adsorbed Donkey anti-Mouse, HRP | Thermo Fisher scientific | SA1100 | WB: 2000x |
| IgG (H+L) Cross-Adsorbed Donkey anti-Rabbit, HRP | Thermo Fisher scientific | SA1200 | WB: 2000x |
| IgG (H+L) Highly Cross-Adsorbed Donkey anti-Mouse, Alexa Fluor™ 488 | Thermo Fisher scientific | A21202 | IF: 500x |
| IgG (H+L) Highly Cross-Adsorbed Donkey anti-Rabbit, Alexa Fluor™ 488 | Thermo Fisher scientific | A21206 | IF: 500x |
| IgG (H+L) Highly Cross-Adsorbed Donkey anti-Mouse, Alexa Fluor™ 594 | Thermo Fisher scientific | A21203 | IF: 500x |
| IgG (H+L) Highly Cross-Adsorbed Donkey anti-Rabbit, Alexa Fluor™ 594 | Thermo Fisher scientific | A21207 | IF: 500x |
| IgG (H+L) Highly Cross-Adsorbed Donkey anti-Mouse, Alexa Fluor™ 647 | Thermo Fisher scientific | A31571 | IF: 500x |
| IgG (H+L) Highly Cross-Adsorbed Donkey anti-Rabbit, Alexa Fluor™ 647 | Thermo Fisher scientific | A31573 | IF: 500x |
| IgG (H+L) Cross-Adsorbed Donkey anti-Goat, Alexa Fluor™ 488 | Thermo Fisher scientific | A11055 | IF: 500x |
| IgG (H+L) Cross-Adsorbed Donkey anti-Goat, Alexa Fluor™ 568 | Thermo Fisher scientific | A11057 | IF: 500x |
| IgG (H+L) Cross-Adsorbed Donkey anti-Goat, Alexa Fluor™ 647 | Thermo Fisher scientific | A21447 | IF: 500x |
