# Supplementary figures and images for: Wiskott-Aldrich syndrome protein forms nuclear condensates and regulates alternative splicing
Source: Nat Commun. 2022 Jun 25;13:3646. doi: 10.1038/s41467-022-31220-8 (PMC9233711; doi:10.1038/s41467-022-31220-8)

Gating Strategy for FACS plots

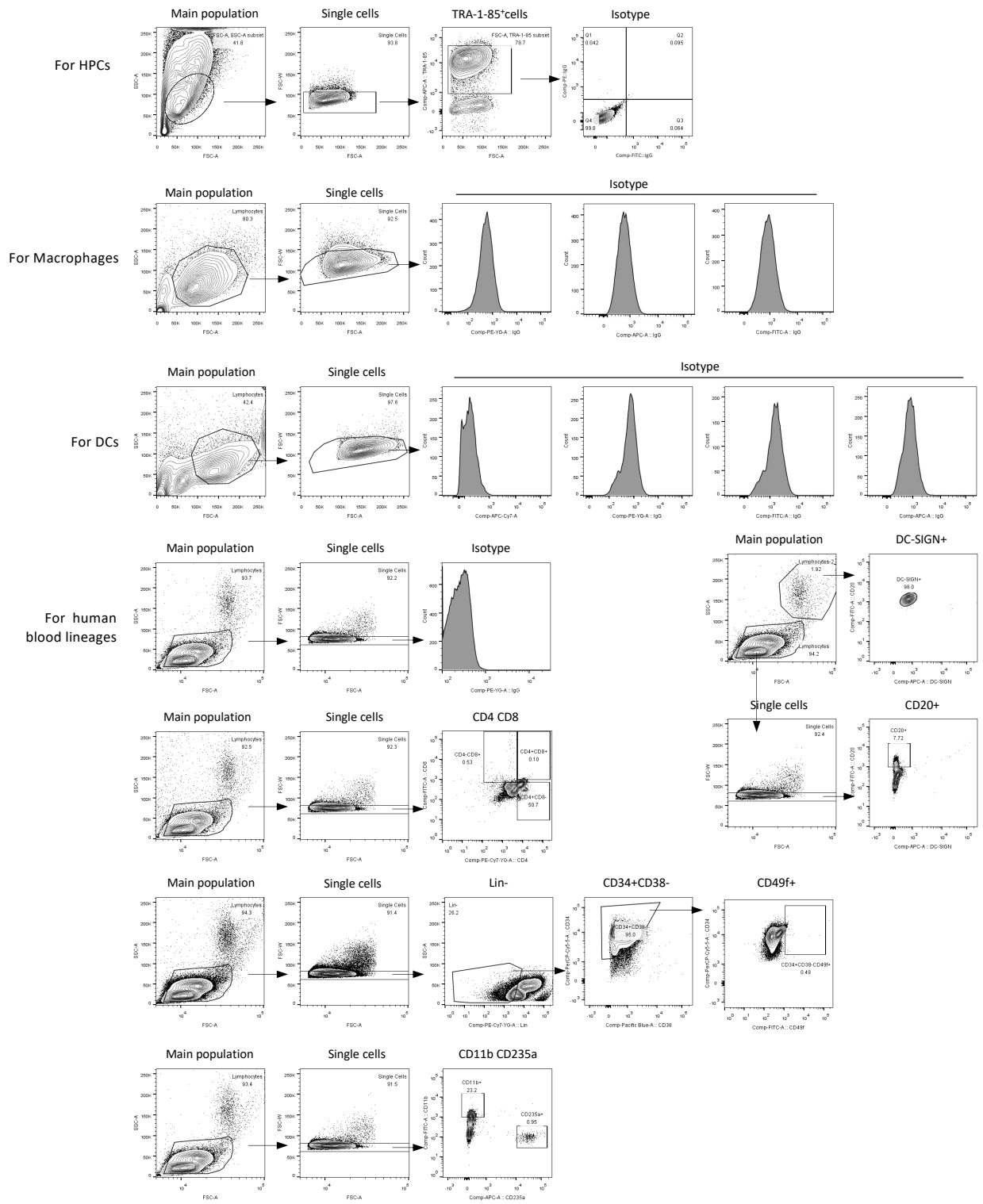

Supplement: Supplementary file 9 — Supplementary Data 7 [file 41467_2022_31220_MOESM9_ESM.pdf]
