# Supplementary material for: Wiskott-Aldrich syndrome protein forms nuclear condensates and regulates alternative splicing
Source: Nat Commun. 2022 Jun 25;13:3646. doi: 10.1038/s41467-022-31220-8 (PMC9233711; doi:10.1038/s41467-022-31220-8)
Supplement: Supplementary file 14 — Reporting Summary [file 41467_2022_31220_MOESM14_ESM.pdf]

nature research

Corresponding author(s): Juan Carlos Izpisua Belmonte, Mo Li

Last updated by author(s): Apr 21, 2022

Reporting Summary

Nature Research wishes to improve the reproducibility of the work that we publish. This form provides structure for consistency and transparency in reporting. For further information on Nature Research policies, see our Editorial Policies and the Editorial Policy Checklist.

Statistics

For all statistical analyses, confirm that the following items are present in the figure legend, table legend, main text, or Methods section.

n/a	Confirmed	
☐	☒	The exact sample size (*n*) for each experimental group/condition, given as a discrete number and unit of measurement
☐	☒	A statement on whether measurements were taken from distinct samples or whether the same sample was measured repeatedly
☐	☒	The statistical test(s) used AND whether they are one- or two-sided<br>*Only common tests should be described solely by name; describe more complex techniques in the Methods section.*
☒	☐	A description of all covariates tested
☐	☒	A description of any assumptions or corrections, such as tests of normality and adjustment for multiple comparisons
☐	☒	A full description of the statistical parameters including central tendency (e.g. means) or other basic estimates (e.g. regression coefficient) AND variation (e.g. standard deviation) or associated estimates of uncertainty (e.g. confidence intervals)
☐	☒	For null hypothesis testing, the test statistic (e.g. *F*, *t*, *r*) with confidence intervals, effect sizes, degrees of freedom and *P* value noted<br>*Give P values as exact values whenever suitable.*
☒	☐	For Bayesian analysis, information on the choice of priors and Markov chain Monte Carlo settings
☒	☐	For hierarchical and complex designs, identification of the appropriate level for tests and full reporting of outcomes
☒	☐	Estimates of effect sizes (e.g. Cohen's *d*, Pearson's *r*), indicating how they were calculated

Our web collection on statistics for biologists contains articles on many of the points above.

Software and code

Policy information about availability of computer code

Data collection	Leica LAS X (v3.5) and ZEISS ZEN blue 2.1 were used for confocal imaging. BioRad CFX Manager (v3.1) was used to perform RT-PCR. Gen5 (v3.02) was used for ELISA assay. BD FACSDiva (v8.02) was used to perform flow cytometry.
Data analysis	ImageJ (version 2.3.0/1.53q) was used for digital image analysis. Graphpad Prim (version 7 and 8) was used for generating plots and statistical analysis. FlowJo (Version 10.8) was used for flow cytometry data analysis. DNASTAR Lasergene (v17.3) and IGV (v2.7.2) were used for ChIPseq analysis and plotting. Avizo (2021.1) was used for analyzing cell 3D information. R (v3.6.3) was used for generating plots.

For manuscripts utilizing custom algorithms or software that are central to the research but not yet described in published literature, software must be made available to editors and reviewers. We strongly encourage code deposition in a community repository (e.g. GitHub). See the Nature Research guidelines for submitting code & software for further information.

Data

Policy information about availability of data

All manuscripts must include a data availability statement. This statement should provide the following information, where applicable:

- Accession codes, unique identifiers, or web links for publicly available datasets
- A list of figures that have associated raw data
- A description of any restrictions on data availability

The human GRCh38 genome (http://ftp.ensembl.org/pub/release-99/fasta/homo_sapiens/) was used as reference genome. The RNAseq data generated in this study including raw sequencing data and the processed gene expression tables have been deposited in the NCBI GEO database under the accession number GSE107963 (https://www.ncbi.nlm.nih.gov/geo/query/acc.cgi?acc=GSE107963) and GSE165382 (https://www.ncbi.nlm.nih.gov/geo/query/acc.cgi?acc=GSE165382), and in NCBI SRA database within the BioProject under the accession code PRJNA541823 (https://www.ncbi.nlm.nih.gov/bioproject/?term=PRJNA541823). Processed ChIP-sequencing data generated in this study have been deposited in the NCBI GEO under the accession number GSE126483

(https://www.ncbi.nlm.nih.gov/geo/query/acc.cgi?acc=GSE126483). Source data are provided with this paper. The mass spectrometry proteomics data generated in this study have been deposited to the ProteomeXchange Consortium via the PRIDE partner repository with the dataset identifier PXD033326 (https://www.ebi.ac.uk/pride/archive/projects/PXD033326). All data is available in the main text or the supplementary materials. The other data that support the findings of this study are available from the corresponding author upon reasonable request.

Field-specific reporting

Please select the one below that is the best fit for your research. If you are not sure, read the appropriate sections before making your selection.

☒ Life sciences ☐ Behavioural & social sciences ☐ Ecological, evolutionary & environmental sciences

For a reference copy of the document with all sections, see nature.com/documents/nr-reporting-summary-flat.pdf

Life sciences study design

All studies must disclose on these points even when the disclosure is negative.

Sample size	The sample sizes of each experiment are indicated throughout the "Methods" section and figure legends. No statistical methods used to predetermine sample size. The sample sizes was chosen to ensure statistical tests (e.g. Student's t-test) could be performed. Sample size was chosen at least 3 independent biological replicates, unless indicated otherwise in the figure legends. All quantitative assays requiring statistical analysis were performed with minimally 3 replicates.
Data exclusions	No data was excluded.
Replication	All replicates are reported in the manuscript. All the experiments are repeated at least 3 time independently, unless indicated otherwise in the figure legends. All attempts at replication were consistent with the reported data.
Randomization	Random fields of microscopy images were analyzed.
Blinding	Blinding was not performed. Investigators knew the genotypes of the cell lines. All experiments were done in vitro, and blinding is not required.

Reporting for specific materials, systems and methods

We require information from authors about some types of materials, experimental systems and methods used in many studies. Here, indicate whether each material, system or method listed is relevant to your study. If you are not sure if a list item applies to your research, read the appropriate section before selecting a response.

Materials & experimental systems

n/a	Involved in the study	
☐	☒	Antibodies
☐	☒	Eukaryotic cell lines
☒	☐	Palaeontology and archaeology
☒	☐	Animals and other organisms
☐	☒	Human research participants
☒	☐	Clinical data
☒	☐	Dual use research of concern

Methods

n/a	Involved in the study	
☐	☒	ChIP-seq
☐	☒	Flow cytometry
☒	☐	MRI-based neuroimaging

Antibodies

Antibodies used

Antibody (vendor, catalog #, dilution)
Immunofluorescence antibodies
anti-WASP (SCBT, #sc-5300, 100x), anti-WASP (Abcam, ab74904, 200x), anti-SC35 (GeneTex, #GTX11826, 500x), anti-SF3B1 (CST, #11434, 300x), anti-PolII (Abcam, ab5095, 100x), anti-cortactin(SCBT, #11408, 200x), anti-Lamin B (SCBT, #sc-8353, 200x), anti-FUS (GeneTex, #101810, 200x), Donkey anti-mouse Alexa Fluor488 (ThermoFisher, A21202), Donkey anti-rabbit Alexa Fluor488 (ThermoFisher, A21206), Donkey anti-goat Alexa Fluor488 (ThermoFisher, A21447), Donkey anti-mouse Alexa Fluor594 (ThermoFisher, A21203),Donkey anti-rabbit Alexa Fluor594 (ThermoFisher, A21207), Donkey anti-goat Alexa Fluor568 (ThermoFisher, A11057), Donkey anti-mouse Alexa Fluor647 (ThermoFisher, A31571), Donkey anti-rabbit Alexa Fluor647 (ThermoFisher, A31573), Donkey anti-goat Alexa Fluor647 (ThermoFisher, A21447),all fluorescent-dye conjugated secondary antibodies were diluted 500x.
Proximity ligation assay antibodies
anti-WASP (SCBT, #sc-5300, 100x) anti-SC35 (Abcam, #ab204916, 100x), anti-SF3B3 (GeneTex, #GTX106450, 100x), anti-hnRNPA2B1 (GeneTex, #GTX127928, 100x), Isotype control: Rabbit IgG, polyclonal (Abcam, #ab37415, 100x)
Western Blotting antibodies
anti-WASP (SCBT, #sc-5300, 500x),anti-WASP (SCBT, #sc-13139, 500x), anti-PPIB (ThermoFisher, #PA1027A, 2000x), anti-SC35 (GeneTex, #GTX11826, 1000x),anti-SC35 (Abcam, #ab204916, 1000x); anti-beta actin (Abcam, #ab8227, 1000x), Donkey anti-rabbit (ThermoFiser SA1-200, 2000x), Donkey anti-mouse HRP (ThermoFisher, #SA1100, 2000x), Donkey anti-rabbit HRP (ThermoFisher,

#SA1200, 2000x)
FACS antibodies
anit-TRA-1-60 PerCP-CyTM5.5 (BD, #561573, 50x), anit-TRA-1-81 PerCP-CyTM5.5 (BD, #561575, 50x), anti-CD11b FITC (eBioscience, #11-0118-41, 50x), anti-CD235a PE (eBioscience, #12-9987-80, 50x), anti-CD209 DC-SIGN (eBioscience, #17-2099-41, 50x), anti-CD14 PE (BioLegend, #301805, 50x), anti-CD43 APC (BD, #560198, 50x), anti-CD45 APC (BD, #555485, 50x), anti-CD34 PE (BD, #555822, 50x), anti-CD115 PE (eBioscience, #12-1159-41, 50x), anti-CD38 eFluor450 (eBioscience, #48-0388-42, 50x). anti-CD34 APC (Mitenyi Biotec, #130-090-954, 50x), anti-CD34-PerCP Vio700 (Miltenyi Biotec, #130-097-915, 50x), anti-CD163 FITC (Miltenyi Biotec, #130-099-969, 50x), anti-CD3 PE (BD, #555333, 50x), anti-CD11c PE (BD, #560999, 50x), anti-CD20 FITC (BD, #555622, 50x), anti-CD8 FITC (BD, #557085, 50x), anti-CD4 PE (BD, #560649, 50x), anti-CD1a AlexaFuor700 (BD, #300120, 50x), mouse monoclonal isotype control APC (BD, #555751, 50x), mouse monoclonal isotype control PE (BD, #555749, 50x), mouse monoclonal isotype control FITC (BD, #555742, 50x).
ChIP antibody:
anti-WASP (anti-WASP (SCBT, #sc-5300, 10 µg/sample), IgG1, Kappa Monoclonal (Abcam, ab91353, 10µg/sample), anti-H3K27ac (Active Motif, #39133, 5 µg/sample), IgG (Abcam, ab37415, 5 µg/sample)

Validation

All the antibodies are commercially available. The dilution of each antibody was tested according to the manufactures' recommendation.
Mouse monoclonal anti-beta Actin antibody (Abcam, ab8227, https://www.abcam.com/beta-actin-antibody-ab8227.html)validated by Western blotting;
Mouse monoclonal anti-WASP antibody (5A5) (BD Biosciences, 557773, https://www.bdbiosciences.com/en-us/products/reagents/flow-cytometry-reagents/research-reagents/single-color-antibodies-ruo/purified-mouse-anti-human-wiskott-aldrich-syndrome-protein.557773), validated by immunoprecipitation;
Rabbit polyclonal anti-WASP antibody (H250) (Santa Cruz Biotechnology, sc-8353, https://www.scbt.com/p/wasp-antibody-h-250?requestFrom=search), validated by Western blotting;
Rabbit IgG (Abcam, ab37415, https://www.abcam.com/rabbit-igg-polyclonal-isotype-control-ab37415.html), validated by immunofluorescence;
Mouse IgG1, Kappa Monoclonal (Abcam, ab91353, https://www.abcam.com/mouse-igg1-kappa-monoclonal-b116-isotype-control-ab91353.html);
Anti-SC35 antibody (Abcam, ab204916, https://www.abcam.com/sc35-antibody-epr12238-ab204916.html), validated by immunofluorescence;
Anti-SC35 antibody (GeneTex, GTX11826, https://www.genetex.com/Product/Detail/SC35-antibody-SC-35/GTX11826), validated by immunofluorescence and Western blotting;
Anti-phospho-RNA polymerase II antibody (Abcam, ab5095, https://www.abcam.com/rna-polymerase-ii-ctd-repeat-ysptsps-phospho-s2-antibody-ab5095.html), validated by immunofluorescence;
Histone H3K27ac antibody (Active Motif, 39133, https://www.activemotif.com/catalog/details/39133), validated by ChIP;
Mouse monoclonal anti-human CD43-APC (BD Biosciences, 560198, https://www.bdbiosciences.com/en-us/products/reagents/flow-cytometry-reagents/research-reagents/single-color-antibodies-ruo/apc-mouse-anti-human-cd43.560198) , validated by Flow cytometry;
Mouse monoclonal anti-CD45-APC (BD Biosciences, 555485, https://www.bdbiosciences.com/en-us/products/reagents/flow-cytometry-reagents/research-reagents/single-color-antibodies-ruo/apc-mouse-anti-human-cd45.555485), validated by Flow cytometry;
Mouse monoclonal anti-CD34-PE (BD Biosciences, 555822, https://www.bdbiosciences.com/en-us/products/reagents/flow-cytometry-reagents/research-reagents/single-color-antibodies-ruo/pe-mouse-anti-human-cd34.555822), validated by Flow cytometry;
Mouse monoclonal anti-human CD3 PE (BD Biosciences, 555333, https://www.bdbiosciences.com/en-us/products/reagents/flow-cytometry-reagents/research-reagents/single-color-antibodies-ruo/pe-mouse-anti-human-cd3.555333), validated by Flow cytometry;
Mouse monoclonal anti-CD11c PE (BD Biosciences, 560999, https://www.bdbiosciences.com/en-us/products/reagents/flow-cytometry-reagents/research-reagents/single-color-antibodies-ruo/pe-mouse-anti-human-cd11c.560999), validated by Flow cytometry;
Mouse monoclonal anti-CD20 FITC (BD Biosciences, 555622, https://www.bdbiosciences.com/en-us/products/reagents/flow-cytometry-reagents/research-reagents/single-color-antibodies-ruo/fitc-mouse-anti-human-cd20.555622), validated by Flow cytometry;
Mouse monoclonal anti-CD8 FITC (BD Biosciences, 557085, https://www.bdbiosciences.com/en-us/products/reagents/flow-cytometry-reagents/research-reagents/single-color-antibodies-ruo/fitc-mouse-anti-human-cd8.557085), validated by Flow cytometry;
Mouse monoclonal anti-CD4 PE (BD Biosciences, 560649, https://www.bdbiosciences.com/en-us/products/reagents/flow-cytometry-reagents/research-reagents/single-color-antibodies-ruo/pe-cy-7-mouse-anti-human-cd4.560649), validated by Flow cytometry;
Mouse monoclonal anti-TRA-1-60 PerCP-CyTM5.5 (BD Biosciences, 561573, https://www.bdbiosciences.com/en-us/products/reagents/flow-cytometry-reagents/research-reagents/single-color-antibodies-ruo/percp-cy-5-5-mouse-anti-human-tra-1-60-antigen.561573), validated by Flow cytometry;
Mouse monoclonal anti-TRA-1-81 PerCP-CyTM5.5 (BD Biosciences, 561575, https://www.bdbiosciences.com/en-us/products/reagents/flow-cytometry-reagents/research-reagents/single-color-antibodies-ruo/percp-cy-5-5-mouse-anti-human-tra-1-81-antigen.561575), validated by Flow cytometry;
Mouse monoclonal isotype control APC (BD Biosciences, 555751, https://www.bdbiosciences.com/en-us/products/reagents/flow-cytometry-reagents/research-reagents/flow-cytometry-controls-and-lysates/apc-mouse-igg1-isotype-control.555751), validated by Flow cytometry;
Mouse monoclonal isotype control PE (BD Biosciences, 555749, https://www.bdbiosciences.com/en-us/products/reagents/flow-cytometry-reagents/research-reagents/flow-cytometry-controls-and-lysates/pe-mouse-igg1-isotype-control.555749), validated by Flow cytometry;
Mouse monoclonal isotype control FITC (BD Biosciences, 555742, https://www.bdbiosciences.com/en-us/products/reagents/flow-cytometry-reagents/research-reagents/flow-cytometry-controls-and-lysates/fitc-mouse-igg2b-isotype-control.555742), validated by Flow cytometry;
Mouse monoclonal anti-CD1a Alexa Fluor 700 (BioLegend, 300120, https://www.biolegend.com/en-us/products/alexa-fluor-700-anti-human-cd1a-antibody-3431), validated by Flow cytometry;
Mouse monoclonal anti-CD14 PE (Biolegend, 301805, https://www.biolegend.com/en-us/products/pe-anti-human-cd14-antibody-796), validated by Flow cytometry;

Mouse monoclonal anti-human CD235a-PE (eBioscience, 12-9987-80, https://www.thermofisher.com/antibody/product/CD235a-Glycophorin-A-Antibody-clone-HIR2-GA-R2-Monoclonal/12-9987-80), validated by Flow cytometry;
Mouse monoclonal anti-human CD11b FITC (eBioscience, 11-0118-41, https://www.thermofisher.com/antibody/product/CD11b-Antibody-clone-ICRF44-Monoclonal/11-0118-41), validated by Flow cytometry;
Mouse monoclonal anti-human CD209 (DC-SIGN) (eBioscience, 17-2099-41, https://www.thermofisher.com/antibody/product/CD209-DC-SIGN-Antibody-clone-eB-h209-Monoclonal/17-2099-41), validated by Flow cytometry;
Mouse monoclonal anti-human CD115 PE (eBioscience, 12-1159-41, https://www.thermofisher.com/antibody/product/CD115-c-fms-Antibody-clone-12-3A3-1B10-Monoclonal/12-1159-41), validated by Flow cytometry;
Mouse monoclonal anti-human CD38 eFluor 450 (eBioscience, 48-0388-42, https://www.thermofisher.com/antibody/product/CD38-Antibody-clone-HB7-Monoclonal/48-0388-42), validated by Flow cytometry;
Mouse monoclonal anti-human CD34-APC (Miltenyi Biotec, 130-090-954, https://www.miltenyibiotec.com/US-en/search.html?activetab=globalSearchFamilies&search=130-090-954#globalSearchFamilies=%5B%5D),validated by Flow cytometry;
Mouse monoclonal anti-human CD34-PerCP Vio700 (Miltenyi Biotec, 130-097-915, https://www.miltenyibiotec.com/US-en/search.html?search=130-097-915&options=on#globalSearchFamilies=%5B%5D), validated by Flow cytometry;
Mouse monoclonal anti-human CD163 FITC (Miltenyi Biotec, 130-099-969, https://www.miltenyibiotec.com/US-en/products/cd163-antibody-anti-human-ghi-61-1.html#fitc:30-tests-in-300-ul), validated by Flow cytometry;
Mouse monoclonal anti-human HLA-DR APC (Miltenyi Biotec, 130-095-297, https://www.miltenyibiotec.com/_Resources/Persistent/1615c123a964672d09dab9ef720f52dff32c506b/DS_HLA-DR_Antibody_anti-human_APC_AC122_130-095-297.pdf), validated by Flow cytometry;
Mouse monoclonal anti-human Tra-1-85 APC (R&D Systems, FAB3195A, https://www.rndsystems.com/products/human-tra-1-85-cd147-apc-conjugated-antibody-tra-1-85_fab3195a), validated by Flow cytometry;
Rabbit polyclonal anti-SF3B3 (Genetex, GTX106450, https://www.genetex.com/Product/Detail/SAP130-antibody-C3-C-term/GTX106450#datasheet), validated by immunofluorescence;
Rabbit polyclonal anti-hnRNPA2B1 (Genetex, GTX127928, https://www.genetex.com/Product/Detail/hnRNP-A2B1-antibody/GTX127928), validated by immunofluorescence;
Rabbit polyclonal anti-cortactin (Santa Cruz Biotechnology, sc-11408, https://www.scbt.com/p/cortactin-antibody-h-191?requestFrom=search), validated by immunofluorescence;
Goat polyclonal anti-Lamin B (M-20) (Santa Cruz Biotechnology, sc-6217, https://www.scbt.com/p/lamin-b-antibody-m-20?requestFrom=search), validated by immunofluorescence;
Rabbit monoclonal anti-N-WASP antibody (Cell Signaling Technology, 4848, https://www.cellsignal.com/products/primary-antibodies/n-wasp-30d10-rabbit-mab/4848), validated by Western blotting;
Rabbit monoclonal anti-human anti-SF3B1 (Cell Signaling Technology, 14434, https://www.cellsignal.com/products/primary-antibodies/sf3b1-d7l5t-rabbit-mab/14434);
Rabbit polyclonal anti-Cyclophilin B antibody (Thermo Fisher, PA1027A, https://www.fishersci.com/shop/products/anti-cyclophilin-b-polyclonal-pa1027a/PA1027A#?keyword=PA1027A), validated by Western blotting;
Mouse monoclonal anti-WASP antibody (D1) (Santa Cruz Biotechnology, sc-5300, https://www.scbt.com/p/wasp-antibody-d-1), validated by Western blotting, immunoprecipitation, and immunofluorescence. This antibody has been used in published paper for ChIP experiment (PMID: 20574068);
Mouse monoclonal anti-WASP antibody (B9) (Santa Cruz Biotechnology, sc-13139, https://www.scbt.com/p/wasp-antibody-b-9?requestFrom=search), validated by Western blotting, immunoprecipitation, and immunofluorescence;
Rabbit polyclonal anti-FUS antibody (GeneTex, 101810 https://www.genetex.com/Product/Detail/FUS-antibody-C1C3/GTX101810), validated by immunofluorescence;
Anti-WASP/Wiskott-Aldrich syndrome protein antibody (Abcam, ab74904, https://www.abcam.com/waspwiskott-aldrich-syndrome-protein-antibody-ab74904.html), validated by immunofluorescence;
Anti-SC35 antibody (Abcam, ab11826, https://www.abcam.com/sc35-antibody-sc-35-nuclear-speckle-marker-ab11826.html), validated by immunofluorescence;
IgG (H+L) Cross-Adsorbed Donkey anti-Mouse, HRP (Thermo Fisher scientific, SA1100, https://www.fishersci.com/shop/products/donkey-anti-mouse-igg-h-l-hrp-polyclonal/SA1100#?keyword=SA1100), validated by Western blotting;
IgG (H+L) Cross-Adsorbed Donkey anti-Rabbit, HRP (Thermo Fisher scientific, SA1200, https://www.fishersci.com/shop/products/donkey-anti-rabbit-igg-h-l-hrp-polyclonal-sa1200/SA1200?searchHijack=true&searchTerm=SA1200&searchType=RAPID&matchedCatNo=SA1200), validated by Western blotting;
IgG (H+L) Highly Cross-Adsorbed Donkey anti-Mouse, Alexa Fluor™ 488 (Thermo Fisher scientific, A21206, https://www.fishersci.com/shop/products/anti-mouse-igg-h-l-alexa-fluor-488-conjugated-polyclonal-thermo-scientific-novex-3/A21202#?keyword=A21202), validated by immunofluorescence;
IgG (H+L) Highly Cross-Adsorbed Donkey anti-Rabbit, Alexa Fluor™ 488 (Thermo Fisher scientific, A21206, https://www.fishersci.com/shop/products/anti-rabbit-igg-h-l-alexa-fluor-488-conjugated-polyclonal-thermo-scientific-novex-1/A21206?searchHijack=true&searchTerm=A21206&searchType=RAPID&matchedCatNo=A21206), validated by immunofluorescence;
IgG (H+L) Highly Cross-Adsorbed Donkey anti-Mouse, Alexa Fluor™ 594 (Thermo Fisher scientific, A21203, https://www.fishersci.com/shop/products/anti-mouse-igg-h-l-alexa-fluor-594-conjugated-polyclonal-thermo-scientific-novex-5/A21203?searchHijack=true&searchTerm=anti-mouse-igg-h-l-alexa-fluor-594-conjugated-polyclonal-thermo-scientific-novex-5&searchType=Rapid&matchedCatNo=A21203), validated by immunofluorescence;
IgG (H+L) Highly Cross-Adsorbed Donkey anti-Rabbit, Alexa Fluor™ 594 (Thermo Fisher scientific, A21207, https://www.fishersci.com/shop/products/anti-rabbit-igg-h-l-alexa-fluor-594-conjugated-polyclonal-thermo-scientific-novex-2/A21207?searchHijack=true&searchTerm=anti-rabbit-igg-h-l-alexa-fluor-594-conjugated-polyclonal-thermo-scientific-novex-2&searchType=Rapid&matchedCatNo=A21207), validated by immunofluorescence;
IgG (H+L) Highly Cross-Adsorbed Donkey anti-Mouse, Alexa Fluor™ 647 (Thermo Fisher scientific, A31571, https://www.fishersci.com/shop/products/anti-mouse-igg-h-l-alexa-fluor-647-conjugated-polyclonal-thermo-scientific-novex-3/A31571#?keyword=A31571), validated by immunofluorescence;
IgG (H+L) Highly Cross-Adsorbed Donkey anti-Rabbit, Alexa Fluor™ 647 (Thermo Fisher scientific, A31573, https://www.fishersci.com/shop/products/anti-rabbit-igg-h-l-alexa-fluor-647-conjugated-polyclonal-thermo-scientific-novex-3/A31573?searchHijack=true&searchTerm=A31573&searchType=RAPID&matchedCatNo=A31573), validated by immunofluorescence;
IgG (H+L) Cross-Adsorbed Donkey anti-Goat, Alexa Fluor™ 488 (Thermo Fisher scientific, A11055, https://www.fishersci.com/shop/products/anti-goat-igg-h-l-alexa-fluor-488-conjugated-polyclonal-thermo-scientific-novex/A11055#?keyword=A11055), validated by immunofluorescence;
IgG (H+L) Cross-Adsorbed Donkey anti-Goat, Alexa Fluor™ 568 (Thermo Fisher scientific, A11057, https://www.fishersci.com/shop/products/anti-goat-igg-h-l-alexa-fluor-568-conjugated-polyclonal-thermo-scientific-novex-1/A11057?

searchHijack=true&searchTerm=anti-goat-igg-h-l-alexa-fluor-568-conjugated-polyclonal-thermo-scientific-novex-1&searchType=Rapid&matchedCatNo=A11057), validated by immunofluorescence;
IgG (H+L) Cross-Adsorbed Donkey anti-Goat, Alexa Fluor™ 647 (Thermo Fisher scientific, A21447, https://www.fishersci.com/shop/products/anti-goat-igg-h-l-alexa-fluor-647-conjugated-polyclonal-thermo-scientific-novex-1/A21447?searchHijack=true&searchTerm=anti-goat-igg-h-l-alexa-fluor-647-conjugated-polyclonal-thermo-scientific-novex-1&searchType=Rapid&matchedCatNo=A21447), validated by immunofluorescence.

Eukaryotic cell lines

Policy information about cell lines

Cell line source(s)	All the cell line sources are disclosed in "Materials and Methods" section. HEK293T cells were purchased from ATCC. ID00003 and GM11518 were purchased from the Coriell institute (Camden, NJ).
Authentication	Sanger sequencing was used to authenticate the mutation in each cell line.
Mycoplasma contamination	Mycoplasma was checked every 2-3 months and was found to be negative in all cell lines used.
Commonly misidentified lines (See ICLAC register)	No cell line is listed in the database of commonly misidentified cell lines.

Human research participants

Policy information about studies involving human research participants

Population characteristics	*Describe the covariate-relevant population characteristics of the human research participants (e.g. age, gender, genotypic information, past and current diagnosis and treatment categories). If you filled out the behavioural & social sciences study design questions and have nothing to add here, write "See above."*
Recruitment	*Describe how participants were recruited. Outline any potential self-selection bias or other biases that may be present and how these are likely to impact results.*
Ethics oversight	*Identify the organization(s) that approved the study protocol.*

Note that full information on the approval of the study protocol must also be provided in the manuscript.

ChIP-seq

Data deposition

☒ Confirm that both raw and final processed data have been deposited in a public database such as GEO.

☒ Confirm that you have deposited or provided access to graph files (e.g. BED files) for the called peaks.

Data access links *May remain private before publication.*	GEO number is GSE126483 (https://www.ncbi.nlm.nih.gov/geo/query/acc.cgi?acc=GSE126483).
Files in database submission	Raw fastq files and BED files
Genome browser session (e.g. UCSC)	n/a

Methodology

Replicates	Two replicates in two lymphoblastoid cell lines. One was done using wild-type cell line and the other using WAS gene mutant (431G>A)] cell line rescued with wild-type WASP (WASP overexpressed)
Sequencing depth	Paired-end 150bp sequencing with 20 million reads per ChIP sample and 10 million reads per input sample.
Antibodies	WASP D-1 antibody (Cat# SC-5300, Santa Cruz Biotechnology)
Peak calling parameters	MACS was used to call ChIP peaks.
Data quality	fastqc was used to examine the fastq quality. We detect 80,711 significant peaks for wild-type lymphoblastoid cell line and 43,138 significant peaks for WASP overexpressed lymphoblastoid cell line.
Software	DNASTAR SeqMan NGen v2.5.1

Flow Cytometry

Plots

Confirm that:

☒ The axis labels state the marker and fluorochrome used (e.g. CD4-FITC).

☒ The axis scales are clearly visible. Include numbers along axes only for bottom left plot of group (a 'group' is an analysis of identical markers).

☒ All plots are contour plots with outliers or pseudocolor plots.

☒ A numerical value for number of cells or percentage (with statistics) is provided.

Methodology

Sample preparation	The cells were stained with antibodies against indicated cell surface markers and DAPI for live/dead cell separation. The stained cells were analyzed on a BD LSRFortessa cytometer or FACSAria Fusion. Cell sorting was performed by the Flow cytometry core facilities of the Salk Institute, UCSD and KAUST on a FACS Aria III cytometer or a FACSAria Fusion cytometer.
Instrument	FACS Fortessa, FACSAria III, FACSAria Fusion
Software	FACSDiva and FlowJo were used to analyze FACS data
Cell population abundance	The purity of post-sort population was checked to be at least 90% by analyzing a few microliters of the sorted cells. At least 1000 events were analyzed.
Gating strategy	Live cells were gated using a viability dye, and then by FSC/SSC for size and granularity. Doublets were excluded based on FSC-W/FSC-A. Isotype controls were used to set the gates for positive cells.

☒ Tick this box to confirm that a figure exemplifying the gating strategy is provided in the Supplementary Information.
